# Supplementary material for: Prognostic value of procalcitonin in acute exacerbation of chronic obstructive pulmonary disease: A systematic review and meta-analysis
Source: PLoS One. 2024 Dec 30;19(12):e0312099. doi: 10.1371/journal.pone.0312099 (PMC11684632; doi:10.1371/journal.pone.0312099)
Supplement: S1 Table — (DOC) [file pone.0312099.s003.doc]

S1 Table. Detailed search history

MEDLINE

Results:247

Database: Pubmed ALL <1949 to July 5, 2022>

1. ((((copd[Title/Abstract]) OR (chronic obstructive pulmonary disease[Title/Abstract])) OR (chronic airflow obstruction[Title/Abstract])) OR (chronic obstructive airway disease[Title/Abstract])) OR (chronic obstructive lung disease[Title/Abstract]) 80046
2. "procalcitonin"[Title/Abstract] OR "pct"[Title/Abstract] OR (("calcitonin"[MeSH Terms] OR "calcitonin"[All Fields] OR "calcitonins"[All Fields] OR "calcitonine"[All Fields]) AND "precursor polyprotein"[Title/Abstract]) OR "calcitonin-1"[Title/Abstract] OR "pro-calcitonin"[Title/Abstract] OR "calcitonin related polypeptide alpha"[Title/Abstract] 14105
3. #1 AND #2 247

EMBASE

Results:1093

Database: Embase Classic+Embase <1966 to July 5, 2022>

1. 'copd'/exp OR copd OR (chronic AND obstructive AND pulmonary AND disease) OR (chronic AND airflow AND obstruction) OR (chronic AND obstructive AND airway AND disease) OR (chronic AND obstructive AND lung AND disease).mp

[mp=title, abstract, heading word, drug trade name, original title, device manufacturer, drug manufacturer, device trade name, keyword, floating

subheading word, candidate term word] (191447)

1. (pct OR procalcitonin OR (calcitonin AND precursor AND polyprotein) OR (calcitonin AND 1) OR 'pro calcitonin' OR (calcitonin AND related AND polypeptide AND alpha)).mp

[mp=title, abstract, heading word, drug trade name, original title, device manufacturer, drug manufacturer, device trade name, keyword, floating

subheading word, candidate term word] (81400)

#1 AND #2 (1093)

[Cochrane Central Register of Controlled Trials](http://www-cochranelibrary-com-s.njykdx.booktsg.com:8118/)

Results:107

Database: Embase Classic+Embase <1976 to July 5, 2022>

1. (copd):ti,ab,kw OR (chronic obstructive pulmonary disease):ti,ab,kw OR (chronic airflow obstruction):ti,ab,kw OR (chronic obstructive airway disease):ti,ab,kw OR (chronic obstructive lung disease):ti,ab,kw (23218)
2. (PCT):ti,ab,kw OR (procalcitonin):ti,ab,kw OR (calcitonin1):ti,ab,kw OR (procalcitonin):ti,ab,kw OR (calcitonin precursor polyprotein):ti,ab,kw (3675)
3. #1 AND #2 (107)
