# Supplementary material for: Prognostic value of procalcitonin in acute exacerbation of chronic obstructive pulmonary disease: A systematic review and meta-analysis
Source: PLoS One. 2024 Dec 30;19(12):e0312099. doi: 10.1371/journal.pone.0312099 (PMC11684632; doi:10.1371/journal.pone.0312099)
Supplement: S2 Table — (DOCX) [file pone.0312099.s004.docx]

S2 Table. Studies identified after excluding duplications (n = 1225)

| **Studies excluded in title and abstract screening with reasons (n = 1189)** | | |
| --- | --- | --- |
| Reasons | Citations | |
| Irrelevant studies (n = 1091) | 1. Abderrahim EK, Manal M, Ghizlane EA, et al. Predictive factors of mortality related to COVID-19: A retrospective cohort study of 600 cases in the intensive care unit of the university hospital of Oujda. Article. *Annals of Medicine and Surgery*. 2021;69doi:10.1016/j.amsu.2021.102711  2. Abers MS, Sandvall BP, Sampath R, et al. Postobstructive Pneumonia: An Underdescribed Syndrome. Article. *Clinical Infectious Diseases*. 2016;62(8):957-961. doi:10.1093/cid/civ1212  3. Acet Öztürk NA, Görek Dilektaşli A, Demirdöğen E, et al. Is serum iron responsive protein-2 level associated with pulmonary functions and frequent exacerbator phenotype in copd? Article. *Tuberkuloz ve Toraks*. 2020;68(3):252-259. doi:10.5578/tt.69934  4. Actrn. Oral resveratrol supplementation in bronchiectasis. *<https://trialsearchwhoint/Trial2aspx?TrialID=ACTRN12620000721909>*. 2020;  5. Actrn. Nasal high flow therapy in bronchiectasis. *<https://trialsearchwhoint/Trial2aspx?TrialID=ACTRN12621001122842>*. 2021;  6. Adepoju VA. Can we use a biomarker to guide antibiotic treatment in severe COPD exacerbations? Article. *Breathe*. 2019;15(4):353-355. doi:10.1183/20734735.0257-2019  7. Adomfeh EC, Francois J, Priyan H, et al. Cardiac injury and mortality among covid-19 patients in a predominantly black community of east flatbush brooklyn. Conference Abstract. *Circulation*. 2021;144(SUPPL 1)doi:10.1161/circ.144.suppl-1.9141  8. Aggarwal D, Mohapatra PR, Aggarwal P. Serum procalcitonin level: Clinical significance. Letter. *European Respiratory Journal*. 2011;37(3):723. doi:10.1183/09031936.00132310  9. Agrafiotis AC, Lardinois I. Pleural empyema caused by Actinomyces turicensis. Article. *New Microbes and New Infections*. 2021;41doi:10.1016/j.nmni.2021.100892  10. Aguado JM, Torres A, Muñoz P, et al. Severe, non-bacteremic infections in ICU patients. Article. *Enfermedades Infecciosas y Microbiologia Clinica*. 2011;29(SUPPL. 4):1-9. doi:10.1016/j.eimc.2010.09.003  11. Agustí A, Sobradillo P, Celli B. Addressing the complexity of chronic obstructive pulmonary disease: From phenotypes and biomarkers to scale-free networks, systems biology, and P4 medicine. Review. *American Journal of Respiratory and Critical Care Medicine*. 2011;183(9):1129-1137. doi:10.1164/rccm.201009-1414PP  12. Ahmed DS, Isnard S, Berini C, Lin J, Routy JP, Royston L. Coping With Stress: The Mitokine GDF-15 as a Biomarker of COVID-19 Severity. Review. *Frontiers in Immunology*. 2022;13doi:10.3389/fimmu.2022.820350  13. Ahsan M, Ng T, Sreekantan Nair A, Fless K. MAC CALLING THE COP: ORGANIZING PNEUMONIA SECONDARY TO MYCOBACTERIUM AVIUM COMPLEX INFECTION. Conference Abstract. *Chest*. 2021;160(4):A336. doi:10.1016/j.chest.2021.07.338  14. Ak Ç, Sayar S, Polat ZP, Kılıç ET, Özdil K. Clinical and laboratory factors associated with severe disease course in Turkish patients with COVID-19 infection. Article. *Iranian Red Crescent Medical Journal*. 2021;23(2)doi:10.32592/ircmj.2021.23.2.283  15. Akcay I, Okoh AK, Yalav O, et al. The prognostic value of pro-calcitonin, CRP and thyroid hormones in secondary peritonitis: A single-center prospective study. Article. *Ulusal Travma ve Acil Cerrahi Dergisi*. 2014;20(5):343-352. doi:10.5505/tjtes.2014.98354  16. Akdeniz S, Sen A. Effect of tocilizumab treatment for COVID-19-induced acute respiratory distress syndrome (ARDS) on renal function of patients. Article. *Tropical Journal of Pharmaceutical Research*. 2022;21(3):629-634. doi:10.4314/tjpr.v21i3.24  17. Akıllı IK, Bilge M. Prognostic Performance of the CALL Score in Hospitalized Patients with COVID-19 Pneumonia. Article. *Medical Journal of Bakirkoy*. 2021;17(4):359-366. doi:10.4274/BMJ.galenos.2021.59354  18. Aksoy A, Al-Kassou B, Zaidi MA, et al. Circulating chaperones in patients with aortic valve stenosis undergoing TAVR: impact of concomitant chronic kidney disease. Article. *Translational Research*. 2021;233:117-126. doi:10.1016/j.trsl.2021.03.003  19. Al Sulaiman K, Aljuhani O, Al Aamer K, et al. The Role of Inhaled Corticosteroids (ICS) in Critically Ill Patients With COVID-19: A Multicenter, Cohort Study. Article. *Journal of Intensive Care Medicine*. 2022;37(2):248-257. doi:10.1177/08850666211053548  20. AlBahrani S, AlAhmadi N, Hamdan S, et al. Clinical Presentation and Outcome of Hospitalized Patients With COVID-19 in the First and Second Waves in Saudi Arabia. Article. *International Journal of Infectious Diseases*. 2022;118:104-108. doi:10.1016/j.ijid.2022.02.048  21. Albrich WC, Dusemund F, Bucher B, et al. Effectiveness and safety of procalcitonin-guided antibiotic therapy in lower respiratory tract infections in "real life": an international, multicenter poststudy survey (ProREAL). Journal: Article. *Archives of internal medicine*. 2012;172(9):715‐722. doi:10.1001/archinternmed.2012.770  22. Albrich WC, Dusemund F, Bucher B, et al. Effectiveness and safety of procalcitonin-guided antibiotic therapy in lower respiratory tract infections in "real life": An international, multicenter poststudy survey (ProREAL). Article. *Archives of Internal Medicine*. 2012;172(9):715-722. doi:10.1001/archinternmed.2012.770  23. Albrich WC, Dusemund F, Bucher B, et al. Procalcitonin-guided antibiotic stewardship in lower respiratory tract infections. A real-life international multicentre quality surveillance (ProREAL). Conference Abstract. *Clinical Microbiology and Infection*. 2011;17:S9. doi:10.1111/j.1469-0691.2011.03557.x  24. Albrich WC, Müller B, Harbarth S. Antibiotic therapy in patients hospitalized with acute chronic obstructive pulmonary disease [3]. Letter. *JAMA - Journal of the American Medical Association*. 2010;304(12):1326. doi:10.1001/jama.2010.1339  25. Al-Darzi W, Aurora L, Michaels A, et al. Heart transplant recipients with confirmed 2019 novel coronavirus infection: The Detroit experience. Article. *Clinical Transplantation*. 2020;34(12)doi:10.1111/ctr.14091  26. Alianza MC, Laya A, Cheng M. Abdominal catastrophe? A case of pneumatosis intestinalis. Conference Abstract. *Journal of Gastroenterology and Hepatology*. 2017;32:144-145. doi:10.1111/jgh.13877  27. Alkhamis A, Alshamali Y, Alyaqout K, et al. Prevalence, predictors and outcomes of bleeding events in patients with COVID-19 infection on anticoagulation: Retrospective cohort study. Article. *Annals of Medicine and Surgery*. 2021;68doi:10.1016/j.amsu.2021.102567  28. Alnabulsi R, Tsushima T, Shaniuk PM. A 60-Year-Old Man With Acute Chest Pain: A Common Presentation, But Unusual Diagnosis. Article. *Chest*. 2021;160(3):e269-e272. doi:10.1016/j.chest.2021.04.017  29. Alonso R, Camon AM, Cardozo C, et al. Clinical Presentation and Outcome of COVID-19 in a Latin American Versus Spanish Population: Matched Case-Control Study. Article. *Infectious Diseases and Therapy*. 2022;11(3):1243-1251. doi:10.1007/s40121-022-00623-x  30. Alsayer RM, Alsharif HM, Al Baadani AM, Kalam KA. Clinical and epidemiological characteristics of COVID-19 mortality in Saudi Arabia. Article. *Saudi Medical Journal*. 2021;42(10):1083-1094. doi:10.15537/SMJ.2021.42.10.20210396  31. Alvarez A, Torcuato R, Salgado M, Alonso A, Fernandez I, Ubeda A. COVID 19: Predictors of mortality in the Intensive Care Unit. Conference Abstract. *Intensive Care Medicine Experimental*. 2021;9(SUPPL 1)doi:10.1186/s40635-021-00415-6  32. Álvarez-Arroyo L, Carrera-Hueso FJ, El-Qutob D, et al. Descriptive study of a cohort of covid-19 hospitalized patients in Spain. Article. *Gaceta Medica de Mexico*. 2021;157(1):76-83. doi:10.24875/GMM.M21000525  33. Andrade D, Kondo AT, Kerbauy L, et al. Treatment of COVID-19 patients: Insights into the use of bone marrow-derived mesenchymal stem cells. Conference Abstract. *Blood*. 2021;138(SUPPL 1):4302. doi:10.1182/blood-2021-154291  34. Andrade HD, Kondo AT, Kerbauy L, et al. Treatment of COVID-19 Patients: Insights into the Use of Bone Marrow-Derived Mesenchymal Stem Cells. Conference Abstract. *Blood*. 2021;138:4302. doi:10.1182/blood-2021-154291  35. Antonio Trigueros Carrero J. How should we define and classify exacerbations in chronic obstructive pulmonary disease? Article. *Expert Review of Respiratory Medicine*. 2013;7(2 SUPPL.):33-41. doi:10.1586/ers.13.16  36. Antonov V, Ignatova G, Belsner M. The impact of vaccination on the dynamics of bronchial and systemic inflammation in patients with COPD and CHD. Conference Abstract. *European Respiratory Journal*. 2017;50doi:10.1183/1393003.congress-2017.PA1063  37. Ara Somohano C, Schwebel C, Vesin A, et al. Use of biomarkers for the diagnosis of infection in case of severe acute dyspnea. Conference Abstract. *Intensive Care Medicine*. 2009;35:S84.  38. Ara Somohano C, Vesi A, Bard C, et al. Impact of biomarkers to predict short-term mortality in patients with severe acute dyspnea. Conference Abstract. *Intensive Care Medicine*. 2011;37:S288.  39. Araújo R, Bento LFN, Fonseca TAH, Von Rekowski CP, da Cunha BR, Calado CRC. Infection Biomarkers Based on Metabolomics. Review. *Metabolites*. 2022;12(2)doi:10.3390/metabo12020092  40. Arbane G, Douiri A, Enright L, Haggis L, Poulter T, Garrod R. Physical activity top up-does it affect exercise tolerance for people with chronic obstructive pulmonary disease (COPD)? *Physiotherapy*. 2011;97:eS395. doi:10.1016/j.physio.2011.04.002  41. Arnold E, Bruton A, Donovan-Hall M, Fenwick A, Dibb B, Walker E. Ambulatory oxygen: Why do COPD patients not use their portable systems as prescribed? A qualitative study. Article. *BMC Pulmonary Medicine*. 2011;11doi:10.1186/1471-2466-11-9  42. Arshad H, Alfonso JCL, Franke R, et al. Decreased plasma phospholipid concentrations and increased acid sphingomyelinase activity are accurate biomarkers for community-acquired pneumonia. Article. *Journal of Translational Medicine*. 2019;17(1)doi:10.1186/s12967-019-2112-z  43. Ashktorab H, Pizuorno A, Adeleye F, et al. Symptomatic, clinical and biomarker associations for mortality in hospitalized COVID-19 patients enriched for African Americans. Article. *BMC Infectious Diseases*. 2022;22(1)doi:10.1186/s12879-022-07520-1  44. Aso S, Matsui H, Fushimi K, Yasunaga H. Dexmedetomidine and Mortality From Sepsis Requiring Mechanical Ventilation: A Japanese Nationwide Retrospective Cohort Study. Article. *Journal of Intensive Care Medicine*. 2021;36(9):1036-1043. doi:10.1177/0885066620942154  45. Aversano MG, Schroeder J, Citterio A, et al. Levofloxacin induced Stevens-Johnson syndrome/ toxic epidermal necrolysis overlap syndrome: Case reports. Conference Abstract. *Clinical and Translational Allergy*. 2014;4:31.  46. Avriel A, Wiessman MP, Almog Y, et al. Admission cell free DNA levels predict 28-day mortality in patients with severe sepsis in intensive care. Article. *PLoS ONE*. 2014;9(6)doi:10.1371/journal.pone.0100514  47. Aydin M, Şaylan B, Ekiz İşcanlı İG. Factors associated with mortality in younger and older (≥75 years) hospitalized patients with community-acquired pneumonia. Article. *Annals of Saudi Medicine*. 2022;42(1):45-51. doi:10.5144/0256-4947.2022.45  48. Aykal G, Esen H, Seyman D, Çaliskan T. Could IL-6 predict the clinical severity of COVID-19? Article. *Turkish Journal of Biochemistry*. 2021;46(5):499-507. doi:10.1515/tjb-2021-0020  49. Az A, Sogut O, Akdemir T, Ergenc H, Dogan Y, Cakirca M. Impacts of demographic and clinical characteristics on disease severity and mortality in patients with confirmed covid-19. Article. *International Journal of General Medicine*. 2021;14:2989-3000. doi:10.2147/IJGM.S317350  50. Azzini AM, Dorizzi RM, Sette P, et al. A 2020 review on the role of procalcitonin in different clinical settings: An update conducted with the tools of the Evidence Based Laboratory Medicine. Review. *Annals of translational medicine*. 2020;8(9)doi:10.21037/atm-20-1855  51. Badawi A, Vasileva D. Comparative profile for COVID-19 cases from China and North America: Clinical symptoms, comorbidities and disease biomarkers. Article. *World Journal of Clinical Cases*. 2021;9(1):118-132. doi:10.12998/wjcc.v9.i1.118  52. Badrick E, Hull S, Mathur R, et al. Health equity audits in general practice: a strategy to reduce health inequalities. Article. *Primary health care research & development*. 2014;15(1):80-95. doi:10.1017/S1463423612000606  53. Bafadhel M, Brightling C. Response. Letter. *Chest*. 2011;140(5):1390-1391. doi:10.1378/chest.10-2979  54. Bafadhel M, Brightling CE. Response. Letter. *Chest*. 2011;140(6):1668. doi:10.1378/chest.11-2492  55. Bafadhel M, Terry S, McKenna S, et al. Biological, physiological and subjective biomarkers in COPD subjects at stable state and exacerbation: An observation. Conference Abstract. *Thorax*. 2009;64:A117-A118. doi:10.1136/thx.2009.127159y  56. Bai M, Wu Y, Ji Z, et al. Prognostic value of C-reactive protein/albumin ratio in neurocritically ill patients. Article. *Minerva Anestesiologica*. 2019;85(12):1299-1307. doi:10.23736/S0375-9393.19.13625-5  57. Balbi B, Di Stefano A, Carone M, Carbone M, Gnemmi I. Systemic biomarkers in acute exacerbations of chronic obstructive pulmonary disease. Conference Paper. *Rassegna di Patologia dell'Apparato Respiratorio*. 2008;23(6):310-313.  58. Bals R, Gillissen A, Lorenz J, et al. COPD and infection: Results of an Expert Meeting, December 4-5, 2009 (Sponsor: Boehringer Ingelheim Pharma GmbH & Co KG). Conference Paper. *Pneumologie*. 2010;64(8):504-520. doi:10.1055/s-0029-1244200  59. Bals R, Gillissen A, Lorenz J, et al. [COPD and infection]. *Pneumologie*. Aug 2010;64(8):504-20. COPD und Infektion. doi:10.1055/s-0029-1244200  60. Bao Z, Chen H, Zhou M, Shi G, Li Q, Wan H. Invasive pulmonary aspergillosis in patients with chronic obstructive pulmonary disease: A case report and review of the literature. Article. *Oncotarget*. 2017;8(23):38069-38074. doi:10.18632/oncotarget.16971  61. Baqdunes MW, Leap J, Young M, Kaura A, Cheema T. Acute Exacerbation of Chronic Obstructive Pulmonary Disease. Article. *Critical Care Nursing Quarterly*. 2021;44(1):74-90. doi:10.1097/CNQ.0000000000000341  62. Baranov D, Trofimov V. PROCALCITONIN AS A MARKER OF INFECTIOUS INFLAMMATION IN PATIENTS WITH BRONCHIAL ASTHMA, COPD, AND ASTHMA-COPD OVERLAP. Conference Abstract. *Chest*. 2022;161(1):A367. doi:10.1016/j.chest.2021.12.397  63. Barton AK, Pelli A, Rieger M, Gehlen H. Procalcitonin as a biomarker in equine chronic pneumopathies. Article. *BMC veterinary research*. 2016;12(1):281.  64. Başgöz BB, Acar R, Aykan MB, et al. Predictive value of thiol–disulfide homeostasis and ischemia-modified albumin on survival in patients with sepsis. Article. *Erciyes Medical Journal*. 2021;43(1):83-89. doi:10.14744/etd.2020.05902  65. Bateman RM, Sharpe MD, Jagger JE, et al. 36th International Symposium on Intensive Care and Emergency Medicine : Brussels, Belgium. 15-18 March 2016. *Critical care (London, England)*. Apr 20 2016;20(Suppl 2):94. doi:10.1186/s13054-016-1208-6  66. Batirel A, Tekin S, Batirel H, Akalan Kuyumcu Ç, Bakan N. The effectiveness of hydroxychloroquine versus vydroxychloroquine plus Lopinavir/ritonavir therapy in SARSCoV- 2 Pneumonia. Article. *Mediterranean Journal of Infection, Microbes and Antimicrobials*. 2021;10doi:10.4274/mjima.galenos.2021.2020.8  67. Bauer TT, Nilius G, Grüning W, Rasche K. Diagnosis and therapy of COPD exacerbation. Review. *Medizinische Klinik - Intensivmedizin und Notfallmedizin*. 2012;107(3):172-178.  68. Baumbach H, Rustenbach CJ, Ahad S, et al. Minimally Invasive Extracorporeal Bypass in Minimally Invasive Heart Valve Operations: A Prospective Randomized Trial. Article. *Annals of Thoracic Surgery*. 2016;102(1):93-100. doi:10.1016/j.athoracsur.2016.01.043  69. Baverstock M, Woodhall N, Maarman V. Do healthcare professionals have sufficient knowledge of inhaler techniques in order to educate their patients effectively in their use? Conference Abstract. *Thorax*. 2010;65:A118. doi:10.1136/thx.2010.150979.45  70. Beasley V, Joshi PV, Singanayagam A, Molyneaux PL, Johnston SL, Mallia P. Lung microbiology and exacerbations in COPD. Review. *International Journal of COPD*. 2012;7:555-569. doi:10.2147/COPD.S28286  71. Beg M, Loucks S, Arif H, Dumont T. A unique case of legionella and streptococcus pneumoniae coinfection. Conference Abstract. *Chest*. 2017;152(4):A112. doi:10.1016/j.chest.2017.08.143  72. Bellamy D, Smith J. Role of primary care in early diagnosis and effective management of COPD. Review. *International Journal of Clinical Practice*. 2007;61(8):1380-1389. doi:10.1111/j.1742-1241.2007.01447.x  73. Bellmann-Weiler R, Ausserwinkler M, Schroecksnadel K, Lass-Floerl C, Weiss G. Usefulness of C-reactive protein and procalcitonin determination for the differential diagnosis and guidance of the clinical management in pneumococcal and Legionella pneumonia. Conference Abstract. *Clinical Microbiology and Infection*. 2009;15:S610-S611. doi:10.1111/j.1469-0691.2009.02858.x  74. Bellou V, Tzoulaki I, Van Smeden M, Moons KGM, Evangelou E, Belbasis L. Prognostic factors for adverse outcomes in patients with COVID-19: A field-wide systematic review and meta-analysis. Review. *European Respiratory Journal*. 2022;59(2)doi:10.1183/13993003.02964-2020  75. Belongia EA, King JP, Kieke BA, et al. Clinical features, severity, and incidence of RSV illness during 12 consecutive seasons in a community cohort of adults ≥60 years old. Article. *Open Forum Infectious Diseases*. 2018;5(12)doi:10.1093/ofid/ofy316  76. Bensaid A, Melhaoui I, Oujidi Y, et al. Acute limb ischemia in patients with COVID-19 pneumonia. Article. *Annals of Medicine and Surgery*. 2021;69doi:10.1016/j.amsu.2021.102747  77. Berg P, Lindhardt B. The role of procalcitonin in adult patients with community-acquired pneumonia--a systematic review. *Dan Med J*. Mar 2012;59(3):A4357.  78. Berg P, Lindhardt BØ. The role of procalcitonin in adult patients with community-acquired pneumonia - A systematic review. Review. *Danish Medical Journal*. 2012;59(3)  79. Bhakta S, Sanghavi DK, Johnson PW, et al. Clinical and laboratory profiles of the SARS-CoV-2 Delta variant compared with pre-Delta variants. Article. *International Journal of Infectious Diseases*. 2022;120:88-95. doi:10.1016/j.ijid.2022.04.050  80. Bhandari S, Bhargava A, Sharma S, Keshwani P, Sharma R, Banerjee S. Clinical Profile of Covid-19 Infected Patients Admitted in a Tertiary Care Hospital in North India. Article. *The Journal of the Association of Physicians of India*. 2020;68(5):13-17.  81. Bhatia HS, Bui QM, King K, DeMaria A, Daniels LB. Subclinical left ventricular dysfunction in COVID-19. Article. *IJC Heart and Vasculature*. 2021;34doi:10.1016/j.ijcha.2021.100770  82. Bhatla A, Mayer MM, Adusumalli S, et al. COVID-19 and cardiac arrhythmias. Article. *Heart Rhythm*. 2020;17(9):1439-1444. doi:10.1016/j.hrthm.2020.06.016  83. Bian W, Chen W, Gu X, et al. [Analysis of related factors of carbapenem resistant Klebsiella pneumoniae infection in patients with artificial airway]. *Zhonghua Wei Zhong Bing Ji Jiu Yi Xue*. Nov 2020;32(11):1324-1330. doi:10.3760/cma.j.cn121430-20200601-00431  84. Bignami E, Bellini V, Maspero G, et al. COVID-19 respiratory support outside the icu’s doors. An observational study for a new operative strategy. Article. *Acta Biomedica*. 2021;92(5)doi:10.23750/abm.v92i5.11417  85. Birajdar AR, Thatte UM, Gogtay NJ. Procalcitonin-guided antibiotic usage-addressing heterogeneity in meta-analysis. Letter. *Indian Journal of Medical Research*. 2018;148(3):348-349. doi:10.4103/ijmr.IJMR_1235_18  86. Bitar M, Fuentes F, Wiltse D. An uncommon cause of drug induced lung injury. Conference Abstract. *American Journal of Respiratory and Critical Care Medicine*. 2019;199(9)  87. Bitar MZ, Al-Ahwel YA. A peculiar cause of atypical pneumonia. Conference Abstract. *American Journal of Respiratory and Critical Care Medicine*. 2019;199(9)  88. Blacharska-Krzanowska KA, Strzepek KA, Woron J, Weber-Lipiec T. How to manage exacerbation of respiratory failure in a patient diagnosed with amyotrophic lateral sclerosis. Article. *Palliative Medicine in Practice*. 2020;14(1):44-57. doi:10.5603/PMPI.2020.0006  89. Black PN. Role of antibiotics in the management of chronic obstructive pulmonary disease. Review. *Expert Review of Respiratory Medicine*. 2008;2(2):235-243. doi:10.1586/17476348.2.2.235  90. Blakemore SP, Gopalan PD. Pandemic influenza A (H1N1) 2009: A case series from intensive care units in port Shepstone, South Africa. Article. *Southern African Journal of Anaesthesia and Analgesia*. 2010;16(3):17-22. doi:10.1080/22201173.2010.10872676  91. Blasi F, Bocchino M, Di Marco F, Richeldi L, Aliberti S. The role of biomarkers in low respiratory tract infections. Review. *European Journal of Internal Medicine*. 2012;23(5):429-435. doi:10.1016/j.ejim.2012.05.002  92. Blasi F, Cosentini R, Migliori GB, Boersma W. Steps forward in LRTI and tuberculosis: Update from the ERS respiratory infections assembly. Review. *European Respiratory Journal*. 2009;33(6):1448-1453. doi:10.1183/09031936.00166908  93. Blasi F, Stolz D, Piffer F. Biomarkers in lower respiratory tract infections. Article. *Pulmonary Pharmacology and Therapeutics*. 2010;23(6):501-507. doi:10.1016/j.pupt.2010.04.007  94. Boachie NA, Johnston JP, Philip SA, Sullivan KV, Heaney M. Characteristics and Incidence of Candidemia in Hospitalized Coronavirus 2019 (COVID-19) Patients. Article. *American Journal of Infectious Diseases*. 2022;18(1)doi:10.3844/ajidsp.2022.1.8  95. Boari GEM, Chiarini G, Bonetti S, et al. Prognostic factors and predictors of outcome in patients with COVID-19 and related pneumonia: A retrospective cohort study. Article. *Bioscience Reports*. 2020;40(12)doi:10.1042/BSR20203455  96. Bobat N, Cubbin I, Lowe DA, Vickers S. A review of the prescribed medicines wastage audit for the NHS community pharmacies of Western Cheshire. Conference Abstract. *International Journal of Pharmacy Practice*. 2011;19:57-58. doi:10.1111/j.2042-7174.2011.00147_1.x  97. Boersma WG. Antibiotics in acute exacerbations of COPD: The good, the bad and the ugly. Editorial. *European Respiratory Journal*. 2012;40(1):1-3. doi:10.1183/09031936.00211911  98. Bogdanovic J, Babic I, Adzic Vukicevic T, Lalic K, Popovic D, Lalic N. IDF21-0532 Hyponatremia severity affects patient outcome in COVID-19 infection. Conference Abstract. *Diabetes Research and Clinical Practice*. 2022;186doi:10.1016/j.diabres.2022.109373  99. Böhning W, Ringe JD, Welzel D, Bode V. Intranasal salmon calcitonin for the prophylaxis of bone mineral loss in steroid-treated chronic obstructive lung diseases. Clinical Trial; Journal Article; Randomized Controlled Trial. *Arzneimittel-Forschung*. 1990;40(9):1000‐1003.  100. Bollu M, Marte-Grau AC, Bobba RK. Procalcitenin-guided antibiotic use in acute respiratory tract infections. Letter. *Archives of Internal Medicine*. 2009;169(7):716. doi:10.1001/archinternmed.2009.27  101. Bongiovanni M, De Lauretis A, Manes G, et al. Clinical characteristics and outcome of COVID-19 pneumonia in elderly subjects. Letter. *Journal of Infection*. 2021;82(2):e33-e34. doi:10.1016/j.jinf.2020.08.023  102. Bouldoires B, Gil H, Soumagne T, Humbert S, Meaux Ruault N, Magy Bertrand N. A predictive bacterial infection score according to eosinophil level: An observational study. Article. *Revue de Medecine Interne*. 2018;39(1):10-16. doi:10.1016/j.revmed.2017.10.425  103. Bouros D, Anevlavis S. Scoring systems in community acquired pneumonia. Editorial. *Pneumon*. 2009;22(4)  104. Boutou AK. Eosinophil Count During Severe Acute COPD Exacerbations: A New Biomarker on the Rise? Letter. *Chest*. 2019;156(6):1276-1277. doi:10.1016/j.chest.2019.08.2172  105. Bowman C, Covington EW. Determination of the Optimal Procalcitonin Threshold for Infection in Patients With Impaired Renal Function at a Community Hospital. Article. *Journal of Pharmacy Technology*. 2020;36(4):157-163. doi:10.1177/8755122520924803  106. Bozan Ö, Atiş ŞE, Çekmen B, et al. Clinical findings and prognosis of hospitalized elderly COVID-19 patients. Article. *Turk Geriatri Dergisi*. 2021;24(1)doi:10.31086/tjgeri.2021.194  107. Bozinovski S, Hutchinson A, Thompson M, et al. Serum amyloid A is a biomarker of acute exacerbations of chronic obstructive pulmonary disease. Article. *American Journal of Respiratory and Critical Care Medicine*. 2008;177(3):269-278. doi:10.1164/rccm.200705-678OC  108. Bozkurt Yilmaz HE, Ünsal ZE, Habeşoğlu MA, Kara S, Şen N. Factors affecting mortality in geriatric patients diagnosed with community-acquired pneumonia treated in intensive care units. Article. *Turk Geriatri Dergisi*. 2021;24(2):212-219. doi:10.31086/TJGERI.2021.217  109. Bremmer D, DiSilvio B, Hammer C, et al. Impact of procalcitonin guidance on management of adults hospitalized with COPD exacerbations. Conference Abstract. *Open Forum Infectious Diseases*. 2017;4:S574. doi:10.1093/ofid/ofxl62.043  110. Bremmer DN, DiSilvio BE, Hammer C, et al. Impact of Procalcitonin Guidance on Management of Adults Hospitalized with Chronic Obstructive Pulmonary Disease Exacerbations. Article. *Journal of General Internal Medicine*. 2018;33(5):692-697. doi:10.1007/s11606-018-4312-2  111. Brightling CE. Biomarkers that predict and guide therapy for exacerbations of chronic obstructive pulmonary disease. Review. *Annals of the American Thoracic Society*. 2013;10(SUPPL):S214-S219. doi:10.1513/AnnalsATS.201302-023AW  112. Brines R, Thorne M. Clinical consensus on COPD. Conference Paper. *Respiratory Medicine: COPD Update*. 2007;3(2):42-48. doi:10.1016/j.rmedu.2007.03.001  113. Bryce A, Foley L, Phillipson J, et al. Clinical features of patients hospitalised with COVID-19 from February to October 2020, during the early waves of the pandemic in New Zealand. Article. *New Zealand Medical Journal*. 2022;135(1552):120-130.  114. Bu XL, Cao GQ, Shen LL, et al. Serum Amyloid-Beta Levels are Increased in Patients with Chronic Obstructive Pulmonary Disease. Article. *Neurotoxicity Research*. 2015;28(4):346-351. doi:10.1007/s12640-015-9552-x  115. Buess M, Schneider T, Maurer M, et al. Treatment of COPD exacerbation in Switzerland - Results and recommendations of the European COPD audit. Conference Abstract. *Respiration*. 2017;94(1):76. doi:10.1159/000474251  116. Busca A, De Rosa FG. Current approach to treatment of febrile neutropenia in hematologic patients. Conference Abstract. *Haematologica*. 2015;100:187-189.  117. Buxton M, Prigmore S, Barnes K, Coombes A, Williams S, Restrick L. Unwarranted variation in chronic obstructive pulmonary disease care: Provision of pulmonary rehabilitation for londoners. Conference Abstract. *Thorax*. 2011;66:A124-A125. doi:10.1136/thoraxjnl-2011-201054c.141  118. Cai R, Li H, Tao Z. Heparin-binding protein and procalcitonin in the diagnosis of pathogens causing community-acquired pneumonia in adult patients: A retrospective study. Article. *PeerJ*. 2021;9doi:10.7717/peerj.11056  119. Calderón-Larrañaga A, Carney L, Soljak M, et al. Association of population and primary healthcare factors with hospital admission rates for chronic obstructive pulmonary disease in England: National cross-sectional study. Article. *Thorax*. 2011;66(3):191-196. doi:10.1136/thx.2010.147058  120. Calonge Contreras M, Billett J, Restrick L, Sennett K, Cooper C, Stern M. Increasing the quality of COPD case finding, diagnosis and management through a primary care financial incentive scheme in inner London. Conference Abstract. *Thorax*. 2011;66:A159. doi:10.1136/thoraxjnl-2011-201054c.223a  121. Candemir I, Turk S, Ergun P, Kaymaz D. Influenza and pneumonia vaccination rates in patients hospitalized with acute respiratory failure. Article. *Human Vaccines and Immunotherapeutics*. 2019;15(11):2606-2611. doi:10.1080/21645515.2019.1613128  122. Çap M, Işik F, Bilge Ö, et al. Serum ionized calcium levels may be more closely related to the admission qtc interval than total calcium levels in patients hospitalized with covid-19. Article. *Eastern Journal of Medicine*. 2021;26(3):433-441. doi:10.5505/ejm.2021.69345  123. Carbonell R, Moreno G, Gómez J, Bodí M, Rodríguez A. Prognostic value of reactive C protein (RCP) and procalcitonin (PCT) in critically ill patients with community-acquired respiratory sepsis. Conference Abstract. *Intensive Care Medicine Experimental*. 2019;7doi:10.1186/s40635-019-0265-y  124. Cardoso J, Ferreira AJ, Guimarães M, Oliveira AS, Simão P, Sucena M. Treatable traits in copd – a proposed approach. Article. *International Journal of COPD*. 2021;16:3167-3182. doi:10.2147/COPD.S330817  125. Carmona-Bayonas A, Jimenez-Fonseca P, de Castro EM, et al. SEOM clinical practice guideline: management and prevention of febrile neutropenia in adults with solid tumors (2018). Article. *Clinical and Translational Oncology*. 2019;21(1):75-86. doi:10.1007/s12094-018-1983-4  126. Carpagnano GE, Pelaia G. VIP: A New Promising Marker for AECOPD-A Fashionable Marker Soon Forgotten? Note. *Respiration*. 2015;90(5):353-354. doi:10.1159/000441307  127. Casas‐aparicio G, Alvarado‐de la Barrera C, Escamilla‐illescas D, et al. Role of Urinary Kidney Stress Biomarkers for Early Recognition of Subclinical Acute Kidney Injury in Critically Ill COVID‐19 Patients. Article. *Biomolecules*. 2022;12(2)doi:10.3390/biom12020275  128. Castro-Castro MJ, García-Tejada L, Arbiol-Roca A, et al. Dynamic profiles and predictive values of some biochemical and haematological quantities in COVID-19 inpatients. Article. *Biochemia Medica*. 2022;32(1)doi:10.11613/BM.2022.010706  129. Caubel A. Acute respiratory failure: diagnosis and treatment in emergency department. Short Survey. *Reanimation*. 2006;15(7-8):523-532. doi:10.1016/j.reaurg.2006.10.007  130. Cazzola M, Rogliani P, Aliberti S, Blasi F, Matera MG. An update on the pharmacotherapeutic management of lower respiratory tract infections. Review. *Expert Opinion on Pharmacotherapy*. 2017;18(10):973-988. doi:10.1080/14656566.2017.1328497  131. Çekiç D, Emir Arman M, Cihad Genç A, et al. Predictive role of FAR ratio in COVID-19 patients. Article. *International Journal of Clinical Practice*. 2021;75(12)doi:10.1111/ijcp.14931  132. Cen Y, Chen X, Shen Y, et al. Risk factors for disease progression in patients with mild to moderate coronavirus disease 2019—a multi-centre observational study. Article. *Clinical Microbiology and Infection*. 2020;26(9):1242-1247. doi:10.1016/j.cmi.2020.05.041  133. Cen Y, Chen X, Shen Y, et al. Risk factors for disease progression in patients with mild to moderate coronavirus disease 2019-a multi-centre observational study. *Clinical microbiology and infection : the official publication of the European Society of Clinical Microbiology and Infectious Diseases*. Sep 2020;26(9):1242-1247. doi:10.1016/j.cmi.2020.05.041  134. Certain L, Schuetz P. The role of procalcitonin in respiratory infections. Article. *Current Infectious Disease Reports*. 2012;14(3):308-316. doi:10.1007/s11908-012-0249-5  135. Cetinkaya A, Uysal MA, Niksarlioglu EY, Durna AS, Ozer NO, Camsari G. Can Neutrophil/lymphocyte Ratio, C-reactive protein (CRP) and Procalcitonin predict the hospitalization time in patients with lower tract respiratory infections ? Conference Abstract. *European Respiratory Journal*. 2019;54doi:10.1183/13993003.congress-2019.PA3849  136. Chakravorty I, Fasakin C, Austin G, Timbury C, Basu C. Impact of mucociliary clearance with high frequency chest wall oscillation in acute exacerbation of COPD. Conference Abstract. *American Journal of Respiratory and Critical Care Medicine*. 2011;183(1)  137. Challans E. How can users be involved in service improvement in health and social care, and why is this important? Article. *Journal of Integrated Care Pathways*. 2006;10(2):49-58. doi:10.1177/205343540601000202  138. Chambers J, Sehring M, Smith NA. Acute pulmonary blastomycosis with ards in an immunocompetent patient. Conference Abstract. *American Journal of Respiratory and Critical Care Medicine*. 2017;195doi:10.1164/ajrccm-conference.2017.A63  139. Chan HP, Lim TK. Procalcitonin and antibiotics in moderate-severe acute exacerbation of chronic obstructive pulmonary disease: To use or not to use. Review. *Current Opinion in Pulmonary Medicine*. 2019;25(2):150-157. doi:10.1097/MCP.0000000000000548  140. Chang C, Yao WZ, Chen YH, Liu ZY, Zhang XW. Value of serum procalcitonin in diagnosing bacterial lower respiratory tract infections in people with exacerbation of Chronic Obstructive Pulmonary Disease. Article. *Beijing da xue xue bao Yi xue ban = Journal of Peking University Health sciences*. 2006;38(4):389-392.  141. Charles MVP, Kali A, Easow JM, et al. Ventilator-associated pneumonia. Review. *Australasian Medical Journal*. 2014;7(8):334-344. doi:10.4066/AMJ.2014.2105  142. Charlotte O, Wegelius H, Landelle C, Schrenzel J, Kaiser L, Pugin J. Outcome of L. pneumophila pneumonia and risk factors for ICU admission. Conference Abstract. *Annals of Intensive Care*. 2016;6doi:10.1186/s13613-016-0114-z  143. Chastain DB, Cluck DB, Stover KR, et al. A baker's dozen of top antimicrobial stewardship intervention publications in 2017. Review. *Open Forum Infectious Diseases*. 2019;6(4)doi:10.1093/ofid/ofz133  144. Chastre J, Blasi F, Masterton RG, Rello J, Torres A, Welte T. European perspective and update on the management of nosocomial pneumonia due to methicillin-resistant Staphylococcus aureus after more than 10 years of experience with linezolid. Review. *Clinical Microbiology and Infection*. 2014;20(S4):19-36. doi:10.1111/1469-0691.12450  145. Chaudhury AR, Pulai S, Gupta S, et al. COVID-19 in hemodialysis: A prospective, observational, single-center data from Eastern India. Letter. *Indian Journal of Nephrology*. 2022;32(1):90-91. doi:10.4103/ijn.IJN_457_20  146. Chauhan A, Monterroso C, Gerardi DA. A diagnostic dilemma: Metastatic pancreatic adenocarcinoma mimicking organizing pneumonia. Conference Abstract. *American Journal of Respiratory and Critical Care Medicine*. 2017;195doi:10.1164/ajrccmconference.2017.C80G  147. Chen B, Liu W, Chen Y, et al. Effect of Poor Nutritional Status and Comorbidities on the Occurrence and Outcome of Pneumonia in Elderly Adults. Article. *Frontiers in medicine*. 2021;8doi:10.3389/fmed.2021.719530  148. Chen K, Pleasants KA, Pleasants RA, et al. Procalcitonin for Antibiotic Prescription in Chronic Obstructive Pulmonary Disease Exacerbations: Systematic Review, Meta-Analysis, and Clinical Perspective. Review. *Pulmonary Therapy*. 2020;6(2):201-214. doi:10.1007/s41030-020-00123-8  149. Chen L, Han XD, Li YL, Zhang CX, Xing XQ. [Incidence and risk factors for cardiovascular events in patients hospitalized with community-acquired pneumonia]. *Zhonghua Xin Xue Guan Bing Za Zhi*. Mar 24 2020;48(3):228-235. doi:10.3760/cma.j.cn112148-20190617-00342  150. Chen L, Luo Q, Shang Y, He X, Xu Y, Gao Z. Predictive and prognostic utility of the serum level of resistin-like molecule beta for risk stratification in patients with community-acquired pneumonia. Article. *Pathogens*. 2021;10(2):1-13. doi:10.3390/pathogens10020122  151. Chen L, Zheng Y, Zhao L, et al. Lipid profiles and differential lipids in serum related to severity of community-acquired pneumonia: A pilot study. Article. *PLoS ONE*. 2021;16(3 March)doi:10.1371/journal.pone.0245770  152. Chen M, Wang T, Chen L, Xu D, Wen F. Elevated BALF PEDF levels are related to disease severity, inflammation and small airway obstruction in COPD patientes. Conference Abstract. *European Respiratory Journal*. 2017;50doi:10.1183/1393003.congress-2017.A3604  153. Chen N, Li H, Zhao LY, et al. Therapy for clearing heat and resolving phlegm in treatment of systemic inflammatory response syndrome in acute deterioration stage of chronic obstructive pulmonary disease: a randomized controlled trial. Journal: Article. *Zhong xi yi jie he xue bao [Journal of Chinese integrative medicine]*. 2009;7(2):105‐109. doi:10.3736/jcim20090202  154. Chen QY, He YS, Liu K, Cao J, Chen YX. Bronchoscopy for diagnosis of COVID-19 with respiratory failure: A case report. Article. *World Journal of Clinical Cases*. 2021;9(5):1132-1138. doi:10.12998/wjcc.v9.i5.1132  155. Chen X, Pan L, Deng H, et al. Risk Assessment in Chinese Hospitalized Patients Comparing the Padua and Caprini Scoring Algorithms. Article. *Clinical and Applied Thrombosis/Hemostasis*. 2018;24(9_suppl):127S-135S. doi:10.1177/1076029618797465  156. Chen Y, Li LIQ, Ge YL, et al. Procalcitonin (PCT) Improves the Accuracy and Sensitivity of Dyspnea, Eosinopenia, Consolidation, Acidemia and Atrial Fibrillation (DECAF) Score in Predicting AECOPD Patients Admission to ICU. *Clin Lab*. Mar 1 2020;66(3)doi:10.7754/Clin.Lab.2019.190612  157. Chen YX, Li CS. Evaluation of community-acquired sepsis by PIRO system in the emergency department. *Intern Emerg Med*. Sep 2013;8(6):521-7. doi:10.1007/s11739-013-0969-z  158. Chen Z, Chen J, Zhou J, et al. A risk score based on baseline risk factors for predicting mortality in COVID-19 patients. Article. *Current Medical Research and Opinion*. 2021;37(6):917-927. doi:10.1080/03007995.2021.1904862  159. Chhetry M, Bhatt R, Tehrani NH, et al. Delayed treatment of acute myocardial infarction with ventricular septal rupture due to patient fear during the covid-19 pandemic. Article. *Texas Heart Institute Journal*. 2021;48(3)doi:10.14503/THIJ-20-7356  160. Chi CI. Positive end-expiratory pressure device combined with ultrasonic nebulization on the sputum treatment of chronic obstructive pulmonary disease (COPD). *<https://trialsearchwhoint/Trial2aspx?TrialID=ChiCTR-IPR-15006319>*. 2015;  161. Chi CI. Efficacy and safety of inhaled N-acetylcysteine on exacerbation of chronic obstructive pulmonary disease (AECOPD), a randomized controlled trial. *<https://trialsearchwhoint/Trial2aspx?TrialID=ChiCTR-IPR-15007616>*. 2015;  162. Chi CI. A random, double-blind, well-compared study of safety and efficacy of Corbrin capsule vs placebo in the treatment of patient with COPD. *<https://trialsearchwhoint/Trial2aspx?TrialID=ChiCTR-IOR-16009282>*. 2016;  163. ChiCtr. A randomized controlled trial for Qingjinhuazhuo formula for shortening course of antibiotic treatment of infectious acute exacerbation of chronic obstructive pulmonary disease (AECOPD). *<https://trialsearchwhoint/Trial2aspx?TrialID=ChiCTR1800019723>*. 2018;  164. ChiCtr. A randomized controlled trial for the effect of Yiqi Huayu Jiedu Recipe, with ultra-low frequency electromagnetic therapy water and infrared spectrum irradiation as adjuvant in the treatment of AECOPD. *<https://trialsearchwhoint/Trial2aspx?TrialID=ChiCTR1900022207>*. 2019;  165. ChiCtr. Qingkepingchuan granules for acute exacerbation of COPD patients with syndrome of phlegm heat obstructing lung: a randomized, double-blind, placebo-controlled trial. *<https://trialsearchwhoint/Trial2aspx?TrialID=ChiCTR2100043009>*. 2021;  166. ChiCtr. A prospective, randomized, double-blind, placebo-controlled and multicenter trial for Tanreqing capsule in the treatment of patients with AECOPD with upper respiratory tract infection. *<https://trialsearchwhoint/Trial2aspx?TrialID=ChiCTR2100050978>*. 2021;  167. Cho J, Lee S, Uh Y, Lee JH. Usefulness of mean platelet volume to platelet count ratio for predicting the risk of mortality in community-acquired pneumonia. Article. *Archives of Medical Science*. 2020;16(6):1327-13335. doi:10.5114/AOMS.2020.92404  168. Choi JJ, McCarthy MW. Novel applications for serum procalcitonin testing in clinical practice. Review. *Expert Review of Molecular Diagnostics*. 2018;18(1):27-34. doi:10.1080/14737159.2018.1407244  169. Choreño-Parra JA, Jiménez-Álvarez LA, Ramírez-Martínez G, et al. Expression of Surfactant Protein D Distinguishes Severe Pandemic Influenza A(H1N1) from Coronavirus Disease 2019. Article. *Journal of Infectious Diseases*. 2021;224(1):21-30. doi:10.1093/infdis/jiab113  170. Choubey A, Sagar D, Cawley P, Miller K. Retrospective review analysis of COVID-19 patients co-infected with Mycoplasma pneumoniae. Review. *Lung India*. 2021;38(7):S22-S26. doi:10.4103/lungindia.lungindia-607-20  171. Chow L, Parulekar AD, Hanania NA. Hospital management of acute exacerbations of chronic obstructive pulmonary disease. Review. *Journal of Hospital Medicine*. 2015;10(5):328-339. doi:10.1002/jhm.2334  172. Christ-Crain M, Jaccard-Stolz D, Bingisser R, et al. Effect of procalcitonin-guided treatment on antibiotic use and outcome in lower respiratory tract infections: cluster-randomised, single-blinded intervention trial. Clinical Trial; Comparative Study; Journal Article; Randomized Controlled Trial; Research Support, Non‐U.S. Gov't. *Lancet (london, england)*. 2004;363(9409):600‐607. doi:10.1016/S0140-6736(04)15591-8  173. Christ-Crain M, Müller B. Procalcitonin in bacterial infections - Hype, hope, more or less? Review. *Swiss Medical Weekly*. 2005;135(31-32):451-460.  174. Christ-Crain M, Müller B. Biomarkers in respiratory tract infections: Diagnostic guides to antibiotic prescription, prognostic markers and mediators. Review. *European Respiratory Journal*. 2007;30(3):556-573. doi:10.1183/09031936.00166106  175. Christ-Crain M, Schuetz P, Huber AR, Müller B. Procalcitonin - Importance for the diagnosis of bacterial infections. Review. *Therapeutische Umschau*. 2008;65(9):559-568. doi:10.1024/0040-930.65.9.559  176. Christiansen AL, Brock A, Bygum A, Rasmussen LM, Jepsen P. Increased mortality in patients with porphyria cutanea tarda—A nationwide cohort study. Article. *Journal of the American Academy of Dermatology*. 2020;83(3):817-823. doi:10.1016/j.jaad.2019.07.082  177. Christiansen AL, Brock A, Bygum A, Rasmussen LM, Jepsen P. Increased mortality in patients with porphyria cutanea tarda-A nationwide cohort study. *J Am Acad Dermatol*. Sep 2020;83(3):817-823. doi:10.1016/j.jaad.2019.07.082  178. Çiçek V, Cinar T, Hayiroglu MI, et al. Preoperative cardiac risk factors associated with in-hospital mortality in elderly patients without heart failure undergoing hip fracture surgery: A single-centre study. Article. *Postgraduate Medical Journal*. 2021;97(1153):701-705. doi:10.1136/postgradmedj-2020-138679  179. Cicero MN, Memmo AI, Piccionello IR, Seminara G, Benfante A, Scichilone N. A79-year-old-man with SARS-CoV-2 pneumonia and unusual pulmonary co-infection. Article. *Minerva Respiratory Medicine*. 2022;61(2):86-91. doi:10.23736/S2784-8477.21.01993-8  180. Cigdem UK, Gamze T. The relationship between different types of nutritional supportive treatment and infection in patients with diagnosis of Chronic Obstructive Pulmonary Disease. Article. *Kuwait Medical Journal*. 2021;53(1):13-17.  181. Cinar O, Cevik E, Acar A, et al. Evaluation of mid-regional pro-adrenomedullin, mid-regional pro-atrial natriuretic peptide, and procalcitonin for the diagnosis and risk stratification of emergency department patients with dyspnea. Conference Abstract. *Annals of Emergency Medicine*. 2011;58(4):S178. doi:10.1016/j.annemergmed.2011.06.027  182. Cingolani A, Tummolo AM, Montemurro G, et al. Baricitinib as rescue therapy in a patient with COVID-19 with no complete response to sarilumab. Article. *Infection*. 2020;48(5):767-771. doi:10.1007/s15010-020-01476-7  183. Clancy K. Experiences of a novice researcher. Review. *Nurse researcher*. 2007;14(4):27-38. doi:10.7748/nr2007.07.14.4.27.c6041  184. Cleland JGF, Zhang J, Hobkirk J, et al. Prognostic significance of plasma concentrations of pro-calcitonin in stable patients with suspected heart failure. Conference Abstract. *European Journal of Heart Failure, Supplement*. 2012;11:S233. doi:10.1093/eurjhf/hss016  185. Clementi A, Brocca A, Virzì GM, et al. Procalcitonin and Interleukin-6 Levels: Are They Useful Biomarkers in Cardiac Surgery Patients? Article. *Blood Purification*. 2017;43(4):290-297. doi:10.1159/000454672  186. Clementi A, Virzì GM, Muciño-Bermejo MJ, et al. Presepsin and Procalcitonin Levels as Markers of Adverse Postoperative Complications and Mortality in Cardiac Surgery Patients. Article. *Blood Purification*. 2019;47(1-3):140-148. doi:10.1159/000494207  187. Coffman KL, Goldenberg R. Septic shock secondary to capnocytophaga gingivales bacteremia in an immunocompetent host. Conference Abstract. *Journal of General Internal Medicine*. 2016;31(2):S725-S726.  188. Çolak A, Yılmaz C, Toprak B, Aktoğu S. Procalcitonin and CRP as Biomarkers in Discrimination of Community-acquired Pneumonia and Exacerbation of COPD. *J Med Biochem*. Apr 2017;36(2):122-126. doi:10.1515/jomb-2017-0011  189. Çolak A, Yllmaz C, Toprak B, Aktoǧu S. Procalcitonin and CRP as Biomarkers in Discrimination of Community-Acquired Pneumonia and Exacerbation of COPD. Article. *Journal of Medical Biochemistry*. 2017;36(2):122-126. doi:10.1515/jomb-2017-0011  190. Conte L, Rossi A, Picariello C, et al. Silent large vegetative mitral-aortic enterococcal endocarditis. Article. *Journal of Cardiovascular Medicine*. 2016;17:e199-e204. doi:10.2459/JCM.0000000000000445  191. Corradi F, Isirdi A, Malacarne P, et al. Low diaphragm muscle mass predicts adverse outcome in patients hospitalized for COVID-19 pneumonia: An exploratory pilot study. Article. *Minerva Anestesiologica*. 2021;87(4):432-438. doi:10.23736/S0375-9393.21.15129-6  192. Corradi M, Acampa O, Goldoni M, et al. Metallic elements in exhaled breath condensate and serum of patients with exacerbation of chronic obstructive pulmonary disease. Article. *Metallomics*. 2009;1(4):339-345. doi:10.1039/b907635b  193. Corrao S, Gervasi F, Bernardo FD, Argano C. Immune response failure in paucisymptomatic long-standing sars-cov-2 spreaders. Article. *Clinics and Practice*. 2021;11(1):151-161. doi:10.3390/clinpract11010021  194. Corti C, Fally M, Fabricius-Bjerre A, et al. Point-of-care procalcitonin test to reduce antibiotic exposure in patients hospitalized with acute exacerbation of COPD. Journal Article; Randomized Controlled Trial. *International journal of chronic obstructive pulmonary disease*. 2016;11:1381‐1389. doi:10.2147/COPD.S104051  195. Cosmi D, Mariottoni B, Angori P, D'Orazio S, Cosmi F. Dexamethasone in acute cardiopulmonary syndrome with hyperinflammatory state. Journal: Conference Abstract. *European heart journal, supplement*. 2021;23(SUPPL C):C30‐C31. doi:10.1093/eurheartj/suab063  196. Cosmi D, Mariottoni B, D'Orazio S, Angori P, Cosmi F. Dexamethasone in acute cardiopulmonary syndrome with hyperinflammatory state. Journal: Conference Abstract. *European heart journal*. 2021;42(SUPPL 1):1049‐. doi:10.1093/eurheartj/ehab724.1049  197. Costa IBSDS, Bittar CS, Rizk SI, et al. The heart and COVID-19: What cardiologists need to know. Review. *Arquivos Brasileiros de Cardiologia*. 2020;114(5):805-816. doi:10.36660/abc.20200279  198. Costales-Cantrell JK, Chung J, Nomura JH, Ironside KR, Salama PW, Sim JJ. Antibiotic Practice Patterns With Procalcitonin Levels in Patients With Acute Lower Respiratory Tract Infection. Article. *American Journal of Managed Care*. 2022;28(2):E35-E41. doi:10.37765/ajmc.2022.88825  199. Covino M, Fransvea P, Rosa F, et al. Early Procalcitonin Assessment in the Emergency Department in Patients with Intra-Abdominal Infection: An Excess or a Need? Article. *Surgical Infections*. 2021;22(8):787-796. doi:10.1089/sur.2020.373  200. Cozzi E, Faccioli E, Marinello S, et al. COVID-19 pneumonia in lung transplant recipients: Report of 2 cases. Article. *American Journal of Transplantation*. 2020;20(10):2933-2937. doi:10.1111/ajt.15993  201. Creamer AW, Kent AE, Albur M. Procalcitonin in respiratory disease: Use as a biomarker for diagnosis and guiding antibiotic therapy. Review. *Breathe*. 2019;15(4):296-304. doi:10.1183/20734735.0258-2019  202. Crisafulli E, Guerrero M, Menéndez R, et al. Inhaled corticosteroids do not influence the early inflammatory response and clinical presentation of hospitalized subjects with COPD exacerbation. Article. *Respiratory Care*. 2014;59(10):1550-1559. doi:10.4187/respcare.03036  203. Crisafulli E, Menéndez R, Huerta A, et al. Systemic inflammatory pattern of patients with community-acquired pneumonia with and without COPD. Article. *Chest*. 2013;143(4):1009-1017. doi:10.1378/chest.12-1684  204. Crown A, Clanton J, Irani S, Rocha F, Alseidi A. Endoscopic transduodenal drainage of the gallbladder. Conference Abstract. *HPB*. 2016;18:e592-e593.  205. Cruz Montesinos S, Del Pozo G, Llano M, et al. Descriptive Study of Hemoperfusion in patients with severe COVID 19. Conference Abstract. *Intensive Care Medicine Experimental*. 2021;9(SUPPL 1)doi:10.1186/s40635-021-00413-8  206. Crystal RG. Airway basal cells: The "smoking gun" of chronic obstructive pulmonary disease. Review. *American Journal of Respiratory and Critical Care Medicine*. 2014;190(12):1355-1362. doi:10.1164/rccm.201408-1492PP  207. Cubbin I, McAlavey A, Wong B. Further investigations on GP prescribing and patient compliance of steroid inhalers for asthma and chronic obstructive pulmonary disease patients within a PCT in North West England. Conference Abstract. *International Journal of Pharmacy Practice*. 2010;18:21-22. doi:10.1111/j.2042-7174.2010.tb00509.x  208. Cui N, Yan R, Qin C, Zhao J. Clinical Characteristics and Immune Responses of 137 Deceased Patients With COVID-19: A Retrospective Study. Article. *Frontiers in cellular and infection microbiology*. 2020;10doi:10.3389/fcimb.2020.595333  209. Culy A, Roberts D, Liew J, Yeoman T. The role of rescue medications in the management of COPD exacerbations. Conference Abstract. *Clinical Pharmacist*. 2010;2(9):S5-S6.  210. Cunha BA. Editorial: Empiric antimicrobial therapy of community-acquired pneumonia: Clinical diagnosis versus procalcitonin levels. Editorial. *Scandinavian Journal of Infectious Diseases*. 2009;41(10):782-784. doi:10.1080/00365540903147035  211. Cunha BA, Chawla K, Jimada I. HIV adult with fever and shortness of breath: Influenza B misdiagnosed as Pneumocystis (carinii) jiroveci pneumonia (PCP). Article. *IDCases*. 2019;17doi:10.1016/j.idcr.2019.e00543  212. da Costa RL, Lamas CDC, Simvoulidis LFN, et al. Secondary infections in a cohort of patients with COVID-19 admitted to an intensive care unit: impact of gram-negative bacterial resistance. Article. *Revista do Instituto de Medicina Tropical de Sao Paulo*. 2022;64doi:10.1590/S1678-9946202264006  213. Dai YS, Zhuang JH, Zheng SM, Wu XY, Chen SC. The effect of enteral nutrition combined with microecologics on patients with chronic obstructive pulmonary disease undergoing mechanical ventilation. *Zhongguo weishengtaxixue zazhi / chinese journal of microecology*. 2018;30:1188‐1192.  214. Dal Negro R, Caputo M, Conti A, Micheletto C. Procalcitonin guided assessment of bacterial etiological role in patients admitted for severe COPD exacerbations a double blind study. *European respiratory journal*. 2007;30(Suppl 51):224s [E1371].  215. Damoiseaux RAMJ. Determination of procalcitonin: Value for reducing ineffective use of antibiotic is questionable. Note. *Nederlands Tijdschrift voor Geneeskunde*. 2009;153(7):268-269.  216. Daniel Markley J, Bernard S, Bearman G, Stevens MP. De-escalating Antibiotic Use in the Inpatient Setting: Strategies, Controversies, and Challenges. Review. *Current Infectious Disease Reports*. 2017;19(4)doi:10.1007/s11908-017-0575-8  217. Daniels JMA, Boersma WG. From the authors. Letter. *American Journal of Respiratory and Critical Care Medicine*. 2010;182(9):1207-1208. doi:10.1164/ajrccm.182.9.1207a  218. Daniels JMA, Schoorl M, Snijders D, et al. Procalcitonin vs C-reactive protein as predictive markers of response to antibiotic therapy in acute exacerbations of COPD. Article. *Chest*. 2010;138(5):1108-1115. doi:10.1378/chest.09-2927  219. Daubin C, Fournel F, Allouche S, et al. Procalcitonin fail to distinguish between bacterial and viral infection in severe AECOPD. Journal: Conference Abstract. *Annals of intensive care*. 2019;9doi:10.1186/s13613-018-0474-7  220. Daubin C, Valette X, Thiollière F, et al. Procalcitonin algorithm to guide initial antibiotic therapy in acute exacerbations of COPD admitted to the ICU: a randomized multicenter study. *Intensive Care Med*. Apr 2018;44(4):428-437. doi:10.1007/s00134-018-5141-9  221. Daubin C, Valette X, Thiollière F, et al. Procalcitonin algorithm to guide initial antibiotic therapy in acute exacerbations of COPD admitted to the ICU: a randomized multicenter study. Article. *Intensive Care Medicine*. 2018;44(4):428-437. doi:10.1007/s00134-018-5141-9  222. Daubin C, Valette X, Thiolliere F, et al. Procalcitonin algorithm to guide initial antibiotic therapy in acute exacerbations of COPD admitted to the ICU: a randomized multicenter study. Article In Press. *Intensive care medicine*. 2018:1‐10. doi:10.1007/s00134-018-5141-9  223. Daubin C, Valette X, Thiollière F, et al. Procalcitonin algorithm to guide initial antibiotic therapy in acute exacerbations of COPD admitted to the ICU: a randomized multicenter study. Comparative Study; Journal Article; Multicenter Study; Randomized Controlled Trial. *Intensive care medicine*. 2018;44(4):428‐437. doi:10.1007/s00134-018-5141-9  224. Davison AG, Jongepier L, Flowers JR, Smith K. A unified ranking system for key COPD outcome measures showing variation between PCTS and hospitals-helping to drive change. Conference Abstract. *Thorax*. 2012;67:A191. doi:10.1136/thoraxjnl-2012-202678.380  225. Davison AG, Jongepier L, Paddison L, Smith K, Flowers JR. Relationship between the ranking of COPD outcome measures (re-admissions and emergency bed days) and respiratory service provision across the 13 PCTS in the east of England (EOE). Conference Abstract. *Thorax*. 2012;67:A189. doi:10.1136/thoraxjnl-2012-202678.375  226. Davison AG, Paddison E, Hanna C, Taylor S. The south east essex (SEE) model of integrated copd care and quip (quality innovation and productivity) improvements. Conference Abstract. *Thorax*. 2010;65:A8. doi:10.1136/thx.2010.150912.11  227. De Wolf HK, Gunnewiek JK, Berk Y, Van Den Ouweland J, De Metz M. Comparison of a new procalcitonin assay from Roche with the established method on the Brahms Kryptor. Letter. *Clinical Chemistry*. 2009;55(5):1043-1044. doi:10.1373/clinchem.2008.117655  228. Del Sole F, Farcomeni A, Loffredo L, et al. Features of severe COVID-19: A systematic review and meta-analysis. Article. *European Journal of Clinical Investigation*. 2020;50(10)doi:10.1111/eci.13378  229. Delerme S, Chenevier-Gobeaux C, Doumenc B, Ray P. Usefulness of B natriuretic peptides and procalcitonin in emergency medicine. Review. *Biomarker Insights*. 2008;2008(3):203-217. doi:10.4137/bmi.s499  230. Delerme S, Chenevier-Gobeaux C, Doumenc B, Ray P. Useulness of B Natriuretic Peptides and Procalcitonin in Emergency Medicine. *Biomark Insights*. Mar 27 2008;3:203-217. doi:10.4137/bmi.s499  231. Delerme S, Ray P. Acute respiratory failure in the elderly: Diagnosis and prognosis. Review. *Age and Ageing*. 2008;37(3):251-257. doi:10.1093/ageing/afn060  232. Deng ML, Liu YW, Chen H, et al. COVID-19 combined with liver injury: Current challenges and management. Article. *World Journal of Clinical Cases*. 2021;9(15):3487-3497. doi:10.12998/wjcc.v9.i15.3487  233. Deng Q, Zhang Y, Wang H, et al. Semiquantitative lung ultrasound scores in the evaluation and follow-up of critically ill patients with COVID-19: a single-center study. Article. *Academic Radiology*. 2020;27(10):1363-1372. doi:10.1016/j.acra.2020.07.002  234. Deng Y, Qiu T, Patel N, Zhou S, Xue T, Zhang H. Clinical management of risk of radiation pneumonia with serum markers during the radiotherapy for patients with thoracic malignant tumors. Article. *Cancer Management and Research*. 2019;11:10249-10256. doi:10.2147/CMAR.S231995  235. Denisova TS, Govorun VM, Bochkarev EG. Possible use of polymerase chain test (DNA diagnosis) in detection of mycobacterium tuberculosis. Article. *Problemy tuberkuleza*. 1999;(6):54-55.  236. Di Domenico G, Leonardi GM, Mottola M, Nocera C. Analysis of the change of the hemochrome parameters in a population of smokers donors. Conference Abstract. *Vox Sanguinis*. 2011;101:113. doi:10.1111/j.1423-0410.2011.01498-2.x  237. Di J, Li X, Xie Y, Yang S, Yu X. Procalcitonin-guided antibiotic therapy in AECOPD patients: Overview of systematic reviews. Article. *Clinical Respiratory Journal*. 2021;15(6):579-594. doi:10.1111/crj.13345  238. Di Pasquale M, Aliberti S, Mantero M, Gramegna A, Blasi F. Pharmacotherapeutic management of bronchial infections in adults: non-cystic fibrosis bronchiectasis and chronic obstructive pulmonary disease. Review. *Expert Opinion on Pharmacotherapy*. 2020;21(16):1975-1990. doi:10.1080/14656566.2020.1793958  239. Diar Bakerly N, Roberts JA. Consultant-led specialist community COPD clinic: Does it work? Conference Abstract. *Thorax*. 2009;64:A164. doi:10.1136/thx.2009.127191v  240. Diar Bakerly N, Thompson A, Roberts JA. Effect of telephone weather alert system on emergency COPD admissions and healthcare utilisation in patients with mild to moderate airflow obstruction. Conference Abstract. *Thorax*. 2009;64:A114-A115. doi:10.1136/thx.2009.127159q  241. Dicken BJ, Zhang J, Hobkirk J, et al. Prognostic significance of plasma concentrations of procalcitonin in patients with suspected heart failure. Conference Abstract. *Heart*. 2013;99:A14. doi:10.1136/heartjnl-2013-304019.15  242. Dickenson J. An exploratory study of patient interventions and nutritional advice for patients with chronic obstructive pulmonary disease, living in the community. Article. *International Journal on Disability and Human Development*. 2009;8(1):43-49.  243. Dieplinger B, Egger M, Gabriel C, et al. Analytical characterization and clinical evaluation of an enzyme-linked immunosorbent assay for measurement of afamin in human plasma. *Clinica chimica acta; international journal of clinical chemistry*. Oct 21 2013;425:236-41. doi:10.1016/j.cca.2013.08.016  244. Ding T, Zhang N. Clinical value analysis of IgM and IgG antibodies detected by nucleic acid in patients with COVID-19. Article. *American Journal of Translational Research*. 2021;13(6):7089-7103.  245. Dixit D, Bridgeman MB, Madduri RP, Kumar ST, Cawley MJ. Pharmacological management and prevention of exacerbations of chronic obstructive pulmonary disease in hospitalized patients. Article. *P and T*. 2016;41(11):703-712.  246. Dobler CC. Biomarkers in respiratory diseases. Editorial. *Breathe*. 2019;15(4):265-266. doi:10.1183/20734735.0329-2019  247. Dodd JW, Hogg L, Nolan J, et al. The COPD assessment test score (CAT): A multicentre, prospective study of response to pulmonary rehabilitation. Conference Abstract. *Thorax*. 2010;65:A36. doi:10.1136/thx.2010.150938.27  248. Doğan HO, Bolat S, Büyüktuna SA, et al. The use of laboratory parameters and computed tomography score to determine intensive care unit requirement in COVID-19. Article. *Turkish Journal of Biochemistry*. 2021;46(2):157-166. doi:10.1515/tjb-2020-0359/html  249. Dogan N, Varol Y, Köktürk N, et al. 2021 Guideline for the Management of COPD Exacerbations: Emergency Medicine Association of Turkey (EMAT) / Turkish Thoracic Society (TTS) Clinical Practice Guideline Task Force. Review. *Turkish Journal of Emergency Medicine*. 2021;21(4):137-176. doi:10.4103/2452-2473.329630  250. Doğanay GE, Cırık MÖ. Is red blood cell distribution width an indicator of prognosis and mortality in respiratory intensive care unit? Article. *European Research Journal*. 2021;7(6):610-616. doi:10.18621/eurj.878028  251. Donadello K, Gottin L, Zanatta P, et al. The veronese paradigm facing COVID-19. Conference Abstract. *Intensive Care Medicine Experimental*. 2020;8(SUPPL 2)doi:10.1186/s40635-020-00354-8  252. Donadello K, Gottin L, Zanatta P, et al. Luck, knowledge and strength facing SARS-CoV-2. the Veronese experience. Conference Abstract. *Critical Care*. 2020;24(SUPPL 2)doi:10.1186/s13054-020-03187-9  253. Dong J. The significance of the inflammatory response in chronic obstructive pulmonary disease-related pulmonary hypertension. Conference Abstract. *Chest*. 2016;149(4):A361. doi:10.1016/j.chest.2016.02.376  254. Doukas SG, Kavali L, Menon RS, Izotov BN, Bukhari A. E-cigarette or vaping induced lung injury: A case series and literature review. Article. *Toxicology Reports*. 2020;7:1381-1386. doi:10.1016/j.toxrep.2020.09.010  255. Douthit NT, Cobia BS. Diagnostic utility of procalcitonin in inpatients at a community hospital. Article. *Clinical Case Reports*. 2019;7(7):1358-1361. doi:10.1002/ccr3.2230  256. Downing A, Rudge G, Cheng Y, Tu YK, Keen J, Gilthorpe MS. Do the UK government's new Quality and Outcomes Framework (QOF) scores adequately measure primary care performance? A cross-sectional survey of routine healthcare data. Article. *BMC Health Services Research*. 2007;7doi:10.1186/1472-6963-7-166  257. Drozdov D, Dusemund F, Müller B, Albrich WC. Efficacy and safety of procalcitonin-guided antibiotic therapy in lower respiratory tract infections. Article. *Antibiotics*. 2013;2(1):1-10. doi:10.3390/antibiotics2010001  258. Du Y, Tu L, Zhu P, et al. Clinical Features of 85 Fatal Cases of COVID-19 from Wuhan: A Retrospective Observational Study. Article. *American Journal of Respiratory and Critical Care Medicine*. 2020;201(11):1372-1379. doi:10.1164/RCCM.202003-0543OC  259. Duan S, Gu X, Fan G, et al. The clinical value of a new molecular point-of-care testing in hospitalized lower respiratory tract infection patients: A randomized controlled study. Conference Abstract. *American Journal of Respiratory and Critical Care Medicine*. 2019;199(9)  260. Duan S, Gu X, Fan G, et al. The clinical value of a new molecular point-of-care testing in hospitalized lower respiratory tract infection patients: a randomized controlled study. Journal: Conference Abstract. *American journal of respiratory and critical care medicine*. 2019;199(9)  261. Dueñas-Castell C, Borre-Naranjo D, Rodelo D, et al. Changes in Oxygenation and Clinical Outcomes with Awake Prone Positioning in Patients with Suspected COVID-19 In Low-Resource Settings: A Retrospective Cohort Study. Article. *Journal of Intensive Care Medicine*. 2021;36(11):1347-1353. doi:10.1177/08850666211049333  262. Durak K, Zayat R, Grottke O, et al. Extracorporeal membrane oxygenation in patients with COVID-19: 1-year experience. Article. *Journal of Thoracic Disease*. 2021;13(10):5911-5924. doi:10.21037/jtd-21-971  263. Dusemund F, Bucher B, Meyer S, et al. Influence of procalcitonin on decision to start antibiotic treatment in patients with a lower respiratory tract infection: Insight from the observational multicentric ProREAL surveillance. Article. *European Journal of Clinical Microbiology and Infectious Diseases*. 2013;32(1):51-60. doi:10.1007/s10096-012-1713-8  264. Duzgun U, Sonkaya A, Öztürk B, et al. The effect of risk factors on the clinical course and treatment of older patients with coronavirus disease 2019. Article. *Neurological Sciences and Neurophysiology*. 2022;39(1):40-47. doi:10.4103/nsn.nsn-114-21  265. Dyer CA, Singh SJ, Stockley RA, Sinclair AJ, Hill SL. The incremental shuttle walking test in elderly people with chronic airflow limitation. Clinical Trial; Controlled Clinical Trial; Journal Article. *Thorax*. 2002;57(1):34‐38. doi:10.1136/thorax.57.1.34  266. Dyer CAE, Singh SJ, Stockley RA, Sinclair AJ, Hill SL. The incremental shuttle walking test in elderly people with chronic airflow limitation. Article. *Thorax*. 2002;57(1):34-38. doi:10.1136/thorax.57.1.34  267. Eastin C, Eastin T. Characteristics and Outcomes of 21 Critically Ill Patients with COVID-19 in Washington State: Arentz M, Yim E, Klaff L, et al. JAMA. Published online March 19, 2020. doi:10.1001/jama.2020.4326. Conference Abstract. *Journal of Emergency Medicine*. 2020;58(4):710. doi:10.1016/j.jemermed.2020.04.002  268. Ebell M. Procalcitonin-guided treatment of respiratory tract infections. Short Survey. *American Family Physician*. 2008;78(6):756-757.  269. Ehsan M, Metersky ML. Management of community-acquired pneumonia. Review. *Current Respiratory Care Reports*. 2013;2(4):218-225. doi:10.1007/s13665-013-0062-0  270. Ekinci I, Uzun H, Utku IK, et al. Prognostic nutritional index as indicator of immune nutritional status of patients with COVID-19. Article. *International Journal for Vitamin and Nutrition Research*. 2022;92(1):4-12. doi:10.1024/0300-9831/a000730  271. El-Chami MF, Shinn TS, Bansal S, et al. LEADLESS PACEMAKER IMPLANT WITH CONCOMITANT ATRIOVENTRICULAR NODE ABLATION: EXPERIENCE WITH THE MICRA TRANSCATHETER PACEMAKER. Conference Abstract. *Heart Rhythm*. 2019;16(5):346-347. doi:10.1016/j.hrthm.2019.04.017  272. Elfaki H, Parsons H, Cawthron K, Holborn A, Pirzada O. Procalcitonin can reduce antibiotic usage in patients with suspected respiratory infections in an acute respiratory service. Conference Abstract. *Thorax*. 2017;72:A147. doi:10.1136/thoraxjnl-2017-210983.260  273. Elikowski W, Fertała N, Zawodna-Marszałek M, et al. Concomitance of COVID-19 and legionnaires' disease - a case series. *Pol Merkur Lekarski*. Feb 22 2022;50(295):30-36.  274. Ende VJ, Singh G, Babatsikos I, et al. Survival of COVID-19 Patients With Respiratory Failure is Related to Temporal Changes in Gas Exchange and Mechanical Ventilation. Article. *Journal of Intensive Care Medicine*. 2021;36(10):1209-1216. doi:10.1177/08850666211033836  275. Endo S, Sato N, Suzuki Y, et al. Application of procalcitonin for pulmonary disease. Review. *Respiration and Circulation*. 2008;56(6):597-600.  276. Enikeev O, Enikeeva S, Enikeev D. T regulatory cells in exacerbation control of chronic obstructive pulmonary disease of mild and moderate stage. Conference Abstract. *European Respiratory Journal*. 2012;40  277. Erdoğan A, Bora ES, Kokkoz Ç, Erdoğan MA, Elmalı F. The importance of procalcitonin in the diagnosis and prognosis of patients with dyspnea in the emergency department. Article. *Annals of Clinical and Analytical Medicine*. 2021;12(8):871-874. doi:10.4328/ACAM.20478  278. Ergan Arsava B, Bilekli S, Alayvaz Aslan N, Er E, Topeli A. Does procalcitonin response to infections change with age? Conference Abstract. *Intensive Care Medicine*. 2010;36:S134. doi:10.1007/s00134-010-1999-x  279. Ergan B, Topeli A. Antibiotic decision in severe chronic obstructive pulmonary disease exacerbations. Letter. *Respiration*. 2017;94(1):75. doi:10.1159/000472719  280. Ergün R, Ergün D, Shalabi HS, et al. COVID-19 and Renal Artery Thrombosis: A Case Report. Article. *Respiratory Case Reports*. 2022;11(1):25-29. doi:10.5505/respircase.2022.40374  281. Ermann DA, Parekh JD, Noble VV, Alghoula F, Anwar MF. Adenocarcinoma of the lung masquerading as aspiration pneumonia. Conference Abstract. *American Journal of Respiratory and Critical Care Medicine*. 2018;197(MeetingAbstracts)  282. Ernst E, Girndt M, Pliquett RU. A case of granulomatosis with polyangiitis complicated by cyclophosphamide toxicity and opportunistic infections: Choosing between Scylla and Charybdis. Article. *BMC Nephrology*. 2014;15(1)doi:10.1186/1471-2369-15-28  283. Eroglu O, Badem ND, Baccioglu A. The diagnostic value of proteoglycan 4 in the differentiation of stable and exacerbation periods in chronic obstructive pulmonary disease: A preliminary study. Article. *Biomedical Research (India)*. 2018;29(19):3561-3567. doi:10.4066/biomedicalresearch.29-17-3970  284. Erturk Sengel B, Ozel S, Gul F, et al. Impact of tocilizumab on clinical outcomes in severe covid-19 patients and risk of secondary infection: A case-control study. Article. *Marmara Medical Journal*. 2021;34(2):112-119. doi:10.5472/marumj.942700  285. Eryavuz D, Abuşoǧlu S, Sivrikaya A, Unlü A. Investigation of procalcitonin levels in patientswith chronic obstructive pulmonary disease. Conference Abstract. *Turkish Journal of Biochemistry*. 2017;42:38.  286. Eshwara VK, Mukhopadhyay C, Rello J. Community-acquired bacterial pneumonia in adults: An update. Review. *Indian Journal of Medical Research*. 2020;151(4):287-302. doi:10.4103/ijmr.IJMR-1678-19  287. Esmaeel HM, Ahmed HA. The refined ABCD assessment and non-costly laboratory parameters are outcome predictors in acute exacerbation of COPD. Article. *Egyptian Journal of Chest Diseases and Tuberculosis*. 2017;66(4):599-603. doi:10.1016/j.ejcdt.2017.06.004  288. Espersen C, Platz E, Skaarup KG, et al. Lung Ultrasound Findings Associated With COVID-19 ARDS, ICU Admission, and All-Cause Mortality. Article. *Respiratory Care*. 2022;67(1):66-75. doi:10.4187/respcare.09108  289. Esposito S, Caramelli F, Principi N. What are the risk factors for admission to the pediatric intensive unit among pediatric patients with COVID-19? Note. *Italian Journal of Pediatrics*. 2021;47(1)doi:10.1186/s13052-021-01057-w  290. Estella A. Cytokine levels in bronchoalveolar lavage and serum in 3 patients with 2009 influenza A(H1N1)v severe pneumonia. Article. *Journal of Infection in Developing Countries*. 2011;5(7):540-543. doi:10.3855/jidc.1618  291. Euctr ES. Clinical trial phase II to evaluate the efficacy of 3 types of treatment in patients with pneumonia by COVID-19. *<https://trialsearchwhoint/Trial2aspx?TrialID=EUCTR2020-001321-31-ES>*. 2020;  292. Evans CE, Peng Y, Zhu MM, Dai Z, Zhang X, Zhao YY. Rabeprazole Promotes Vascular Repair and Resolution of Sepsis-Induced Inflammatory Lung Injury through HIF-1α. Article. *Cells*. 2022;11(9)doi:10.3390/cells11091425  293. Ewig S. Pneumonia alerts. Short Survey. *Pneumologie*. 2007;61(9):610-612. doi:10.1055/s-2007-980068  294. Fally M, Corti C, Fabricius-Bjerre A, Mortensen K, Jensen BN, Andreassen H. Point-of-care procalcitonin test to reduce antibiotics in COPD exacerbation: a quasi-randomised control trial. *European respiratory journal*. 2015;46  295. Fally M, Corti C, Fabricius-Bjerre A, et al. Point-of-care procalcitonin test to reduce antibiotics in COPD exacerbation: A quasi-randomised control trial. Conference Abstract. *European Respiratory Journal*. 2015;46doi:10.1183/13993003.congress2015.OA4752  296. Falsey AR, Becker KL, Swinburne AJ, et al. Bacterial complications of respiratory tract viral illness: A comprehensive evaluation. Article. *Journal of Infectious Diseases*. 2013;208(3):432-441. doi:10.1093/infdis/jit190  297. Falsey AR, Walsh EE. Reply to Musher et al. Letter. *Journal of Infectious Diseases*. 2014;209(4):633-634. doi:10.1093/infdis/jit580  298. Falzon C, Soljak M, Elkin SL, Blake ID, Hopkinson NS. Finding the missing millions-the impact of a locally enhanced service for COPD on current and projected rates of diagnosis. Conference Abstract. *Thorax*. 2012;67:A191-A192. doi:10.1136/thoraxjnl-2012-202678.381  299. Falzon C, Soljak M, Elkin SL, Blake ID, Hopkinson NS. Finding the missing millions - The impact of a locally enhanced service for COPD on current and projected rates of diagnosis: A population-based prevalence study using interrupted time series analysis. Article. *Primary Care Respiratory Journal*. 2013;22(1):59-63. doi:10.4104/pcrj.2013.00008  300. Fan Y, Guo T, Yan F, et al. Association of Statin Use With the In-Hospital Outcomes of 2019-Coronavirus Disease Patients: A Retrospective Study. Article. *Frontiers in medicine*. 2020;7doi:10.3389/fmed.2020.584870  301. Farré N, Mojón D, Llagostera M, et al. Prolonged qt interval in sars-cov-2 infection: Prevalence and prognosis. Article. *Journal of Clinical Medicine*. 2020;9(9):1-15. doi:10.3390/jcm9092712  302. Fazili T, Endy T, Javaid W, Maskey M. Role of procalcitonin in guiding antibiotic therapy. Article. *American Journal of Health-System Pharmacy*. 2012;69(23):2057-2061. doi:10.2146/ajhp110736  303. Feng C, Xu M, Kang J, et al. Atypical pathogen distribution in chinese hospitalized aecopd patients: A multicenter cross-sectional study. Article. *International Journal of COPD*. 2021;16:1699-1708. doi:10.2147/COPD.S300779  304. Feng J, Luo FM, Yan H. Procalcitonin guided antibiotics therapy in patients with acute exacerbation chronic obstructive pulmonary disease: A meta-analysis. Review. *Chinese Journal of Evidence-Based Medicine*. 2016;16(10):1162-1168. doi:10.7507/1672-2531.20160177  305. Feng Z, Li J, Yao S, et al. Clinical factors associated with progression and prolonged viral shedding in COVID-19 patients: A multicenter study. Article. *Aging and Disease*. 2020;11(5):1069-1081. doi:10.14336/AD.2020.0630  306. Ferré A. COPD. Article. *Revue des Maladies Respiratoires Actualites*. 2012;4(1):9-14. doi:10.1016/S1877-1203(12)70175-1  307. Ferreira JP, Metra M, Anker SD, et al. Clinical correlates and outcome associated with changes in 6-minute walking distance in patients with heart failure: findings from the BIOSTAT-CHF study. Article. *European Journal of Heart Failure*. 2019;21(2):218-226. doi:10.1002/ejhf.1380  308. Ferrer M, Torres A, Martínez R, et al. Inhaled corticosteroids and systemic inflammatory response in community-acquired pneumonia: A prospective clinical study. Article. *Respirology*. 2014;19(6):929-935. doi:10.1111/resp.12324  309. Ferrer M, Torres A, Reyes S, et al. Inhaled corticosteroids (ICS), systemic inflammatory response and mortality in community-acquired pneumonia (CAP). Conference Abstract. *European Respiratory Journal*. 2011;38  310. Fiaschè F, Adriani B, Mancinelli I, Taranto A. Treatment of catatonia with asenapine in a patient with schizotypal personality disorder, psychotic depression and septic shock from sars-cov-2-a case report. Article. *CNS and Neurological Disorders - Drug Targets*. 2021;20(5):473-477. doi:10.2174/1871527320666210226114238  311. Flatley M. Procalcitonin-guided antibiotic use for respiratory tract infections. Article. *Thorax*. 2009;64(4):367. doi:10.1136/thx.2008.102996  312. Flowers JR, Smith K, Davison AG. Is your COPD service improving or deteriorating? Trend analysis of mortality, bed-days and readmissions shows great variability across England during the last decade. Conference Abstract. *Thorax*. 2012;67:A190-A191. doi:10.1136/thoraxjnl-2012-202678.379  313. Fordham M, Sullivan L, Hawkins P, Jongepier L. Sustained cost reductions from a new home oxygen service during the first operating year. Conference Abstract. *COPD: Journal of Chronic Obstructive Pulmonary Disease*. 2011;8(1):39. doi:10.3109/15412555.2011.541141  314. Forini G, Torsani F, Aspidistria L, Bellini F, Contoli M. Procalcitonin-guided treatment on antibiotic use in COPD exacerbations. Note. *Rassegna di Patologia dell'Apparato Respiratorio*. 2016;31(6):295-298.  315. Frantzeskaki F, Orfanos SE. Treating nosocomial pneumonia: What’s new. Note. *ERJ Open Research*. 2018;4(2)doi:10.1183/23120541.00058-2018  316. Froiio C, Bernardi DT, Asti E, Bonavina L. Retroperitoneoscopic drainage of cryptogenic psoas abscess. Article. *BMJ Case Reports*. 2020;13(7)doi:10.1136/bcr-2020-235579  317. Fu Z, Lin Y, Liu Z, et al. The effect of airway suction nursing care for copd patients with airway mucus hypersecretion. Journal: Article. *International Journal of Clinical and Experimental Medicine*. 2020;13(11):8627‐8635.  318. Gacche RN, Gacche RA, Chen J, Li H, Li G. Predictors of morbidity and mortality in COVID-19. Article. *European Review for Medical and Pharmacological Sciences*. 2021;25(3):1684-1707. doi:10.26355/eurrev_202102_24880  319. Gani R, Griffin J, Kelly S, Mölken MRV. Economic analyses comparing tiotropium with ipratropium or salmeterol in UK patients with COPD. Article. *Primary Care Respiratory Journal*. 2010;19(1):68-74. doi:10.4104/pcrj.2010.00001  320. Gani R, Griffin J, Kelly S, Rutten-van Mölken M. Economic analyses comparing tiotropium with ipratropium or salmeterol in UK patients with COPD. *Primary care respiratory journal : journal of the General Practice Airways Group*. Mar 2010;19(1):68-74. doi:10.4104/pcrj.2010.00001  321. Gao D, Chen X, Wu H, Wei H, Wu J. The levels of serum pro-calcitonin and high-sensitivity c-reactive protein in the early diagnosis of chronic obstructive pulmonary disease during acute exacerbation. Article. *Experimental and Therapeutic Medicine*. 2017;14(1):193-198. doi:10.3892/etm.2017.4496  322. Gao SL, Wang YH, Li CY, et al. A highly significant association between Cathepsin S gene polymorphisms rs12068264 and chronic obstructive pulmonary disease susceptibility in Han Chinese population. Article. *Bioscience Reports*. 2018;38(4)doi:10.1042/BSR20180410  323. Gao YD, Ding M, Dong X, et al. Risk factors for severe and critically ill COVID-19 patients: A review. Review. *Allergy: European Journal of Allergy and Clinical Immunology*. 2021;76(2):428-455. doi:10.1111/all.14657  324. Gauthier A, Jaubert J, Traversier N, et al. Trametes polyzona, an emerging filamentous basidiomycete in Réunion Island. Article. *Mycoses*. 2017;60(6):412-415. doi:10.1111/myc.12609  325. Geissler HJ. Invited commentary. Note. *Annals of Thoracic Surgery*. 2015;99(4):1313. doi:10.1016/j.athoracsur.2014.12.001  326. Genç AB, Yaylaci S, Dheır H, et al. The predictive and diagnostic accuracy of long pentraxin-3 in COVID-19 pneumonia. Article. *Turkish Journal of Medical Sciences*. 2021;51(2):448-453. doi:10.3906/sag-2011-32  327. Geng L, He C, Kan H, et al. The association between blood pressure levels and mortality in critically ill patients with COVID-19 in Wuhan, China: a case-series report. Letter. *Hypertension Research*. 2021;44(3):368-370. doi:10.1038/s41440-020-00594-x  328. Genova VP, Manolov V, Georgiev O. Copeptin - a marker for differential diagnosis between acute exacerbation of COPD and heart failure in patients with acute dyspnea. Conference Abstract. *European Respiratory Journal*. 2021;58(SUPPL 65)doi:10.1183/13993003.congress-2021.PA1147  329. Gernone G, Detomaso F, Partipilo F, Montemurro M, Procino F, Diele C. POS-853 AKI due to COVID-19 disease requiring Renal Replacement Therapy: role of Expanded HaemoDialisys (HDx) on inflammation and outcome. Conference Abstract. *Kidney International Reports*. 2022;7(2):S368-S369. doi:10.1016/j.ekir.2022.01.891  330. Gerontitis C, Pencu ED, De Saint Hubert G. Paraneoplasic hyperleukocytosis reaction in a case of Lynch syndrome. Conference Abstract. *Acta Clinica Belgica: International Journal of Clinical and Laboratory Medicine*. 2018;73:52. doi:10.1080/17843286.2018.1542267  331. Gerotziafas GT, Catalano M, Colgan MP, et al. Guidance for the Management of Patients with Vascular Disease or Cardiovascular Risk Factors and COVID-19: Position Paper from VAS-European Independent Foundation in Angiology/Vascular Medicine. Review. *Thrombosis and Haemostasis*. 2020;120(12):1597-1628. doi:10.1055/s-0040-1715798  332. Gessner C, Hammerschmidt S, Kuhn H, et al. Exhaled breath condensate nitrite and its relation to tidal volume in acute lung injury. Article. *Chest*. 2003;124(3):1046-1052. doi:10.1378/chest.124.3.1046  333. Gessner C, Hammerschmidt S, Kuhn H, et al. Nitrite in exhaled breath condensate - A marker for mechanical stress in mechanical ventilation. Article. *Intensivmedizin und Notfallmedizin*. 2006;43(4):270-278. doi:10.1007/s00390-006-0713-2  334. Gholami B, Gholami S, Loghman AH, et al. Clinical and Laboratory Predictors of Severity, Criticality, and Mortality in COVID-19: A Multisystem Disease. 2021. p. 369-402.  335. Gholami B, Gholami S, Loghman AH, et al. Clinical and Laboratory Predictors of Severity, Criticality, and Mortality in COVID-19: A Multisystem Disease. *Advances in experimental medicine and biology*. 2021;1318:369-402. doi:10.1007/978-3-030-63761-3_22  336. Giacomelli A, Bogaart LVD, Corbellino M, et al. Is there a role for procalcitonin determination in avoiding unnecessary exposure to antibiotics in a non-intensive care setting? Article. *Infezioni in Medicina*. 2019;27(2):128-133.  337. Gillespie D, Francis NA, Carrol ED, Thomas-Jones E, Butler CC, Hood K. Use of co-primary outcomes for trials of antimicrobial stewardship interventions. Note. *The Lancet Infectious Diseases*. 2018;18(6):595-597. doi:10.1016/S1473-3099(18)30289-5  338. Gillissen A, Wiechmann V, Jürgens UR. Biomarker in pulmonary diseases. Review. *Pneumologie*. 2009;63(8):439-450. doi:10.1055/s-0029-1214799  339. Giorgi-Pierfranceschi M, Cravo J, Dentali F, Esquinas AM. Is Procalcitonin Really Useful for Diagnosis and Prognosis of COPD Exacerbations Requiring Mechanical Ventilation? Letter. *Respiration*. 2017;93(2):151-152. doi:10.1159/000454839  340. Girault C. Nosocomial tracheobronchitis and acute exacerbations of COPD. Conference Paper. *Revue des Maladies Respiratoires*. 2004;21(5 II):7S187-7S188.  341. Goel M, Aggarwal A, Chawla R, et al. Utilization of procalcitonin to guide antibiotic discontinuation in respiratory tract infections in a community hospital. Conference Abstract. *American Journal of Respiratory and Critical Care Medicine*. 2021;203(9)doi:10.1164/ajrccm-conference.2021.203.1_MeetingAbstracts.A3907  342. Goel M, Aggarwal A, Chawla R, et al. Utilization of procalcitonin to guide antibiotic discontinuation in respiratory tract infections in a community hospital. Conference Abstract. *Journal of General Internal Medicine*. 2021;36(SUPPL 1):S140-S141. doi:10.1007/s11606-021-06830-5  343. Goh SZF, Tan BH, Stacey S. Association between non-typhoidal Salmonella urinary tract infection and Klebsiella pneumoniae lung empyema. Article. *Proceedings of Singapore Healthcare*. 2021;30(3):206-208. doi:10.1177/2010105820964527  344. Gökbulut Bektaş Ş, İzdeş S. The effect of the vitamin d level on the clinical, laboratory, and outcome in patients over 65 years of age with covid-19: Comprehensive tertiary center experience. Article. *Turk Geriatri Dergisi*. 2021;24(4):567-577. doi:10.31086/tjgeri.2021.254  345. Goncalves Mendes Neto A, Lo KB, Wattoo A, et al. Bacterial infections and patterns of antibiotic use in patients with COVID-19. Article. *Journal of Medical Virology*. 2021;93(3):1489-1495. doi:10.1002/jmv.26441  346. Gonzalez JLB, Gámez MG, Enciso EAM, et al. Efficacy and Safety of Ivermectin and Hydroxychloroquine in Patients with Severe COVID-19: A Randomized Controlled Trial. Article. *Infectious Disease Reports*. 2022;14(2):160-168. doi:10.3390/idr14020020  347. Gonzalez-Rey E, Delgado-Maroto V, Souza-Moreira L, Delgado M. Neuropeptides as therapeutic approach to autoimmune diseases. Review. *Current Pharmaceutical Design*. 2010;16(28):3158-3172. doi:10.2174/138161210793292465  348. Gonzalez-Rey E, Ganea D, Delgado M. Neuropeptides: Keeping the balance between pathogen immunity and immune tolerance. Review. *Current Opinion in Pharmacology*. 2010;10(4):473-481. doi:10.1016/j.coph.2010.03.003  349. Görgülü Ö, Duyan M. Effects of Comorbid Factors on Prognosis of Three Different Geriatric Groups with COVID-19 Diagnosis. Article. *SN Comprehensive Clinical Medicine*. 2020;2(12):2583-2594. doi:10.1007/s42399-020-00645-x  350. Gouse BM, Spears WE, Nieves Archibald A, Montalvo C. Catatonia in a hospitalized patient with COVID-19 and proposed immune-mediated mechanism. Letter. *Brain, Behavior, and Immunity*. 2020;89:529-530. doi:10.1016/j.bbi.2020.08.007  351. Grace E, Turner RM. Use of procalcitonin in patients with various degrees of chronic kidney disease including renal replacement therapy. *Clinical infectious diseases : an official publication of the Infectious Diseases Society of America*. Dec 15 2014;59(12):1761-7. doi:10.1093/cid/ciu732  352. Green D, Hampson O, Agarwal S, Church S. Cost effectiveness of overnight face-to-face nurse led COPD service. Conference Abstract. *Thorax*. 2011;66:A106. doi:10.1136/thoraxjnl-2011-201054c.95  353. Gregory Elliott C, Parsons PE. Update in pulmonary and critical care medicine: Evidence published in 2011. Article. *Annals of Internal Medicine*. 2012;156(10):736-742. doi:10.7326/0003-4819-156-10-201205150-00414  354. Greulich T, Koczulla AR, Vogelmeier C. Chronic obstructive pulmonary disease. New pharmacotherapeutic options. Article. *Internist*. 2012;53(11):1364-1375. doi:10.1007/s00108-012-3119-1  355. Griffith DM, Vale ME, Campbell C, Lewis S, Walsh TS. Persistent inflammation and recovery after intensive care: A systematic review. Article. *Journal of Critical Care*. 2016;33:192-199. doi:10.1016/j.jcrc.2016.01.011  356. Griffiths C, Pass S, Yarbrough W. Original article: Assessment of the intensive care unit treatment of pneumonia at a veterans affairs facility. Article. *Hospital Pharmacy*. 2014;49(10):950-955. doi:10.1310/hpj4910-950  357. Guartazaca-Guerrero S, Rodríguez-Morales J, Rizo-Téllez SA, et al. High levels of IL-8 and MCP-1 in cerebrospinal fluid of COVID-19 patients with cerebrovascular disease. Article. *Experimental Neurobiology*. 2021;30(3):256-261. doi:10.5607/en21009  358. Guglielmetti L, Kontsevaya I, Leoni MC, et al. Severe COVID-19 pneumonia in Piacenza, Italy — A cohort study of the first pandemic wave. Article. *Journal of Infection and Public Health*. 2021;14(2):263-270. doi:10.1016/j.jiph.2020.11.012  359. Gulrajani R. Do people with mild COPD benefit from early pulmonary rehabilitation programmes? Article. *Nursing times*. 2010;106(17):16-17.  360. Gültekin Y, Biri İ, Gojayev A, Yilmaz Işikhan S, Portakal Akçin O, Kiliç YA. Can omentin-1 be a prognostic marker in surgical intensive care patients? Article. *Turkish Journal of Medical Sciences*. 2021;51(5):2485-2493. doi:10.3906/SAG-2009-158  361. Gundogan K, Emur Gunay Y, Ozer NT, et al. Serum trace elements levels in patients transferred from the intensive care unit to wards. Article. *Clinical Nutrition ESPEN*. 2021;44:218-223. doi:10.1016/j.clnesp.2021.06.014  362. Güner R, Hasanoğlu İ, Kayaaslan B, et al. COVID-19 experience of the major pandemic response center in the capital: Results of the pandemic’s first month in Turkey. Article. *Turkish Journal of Medical Sciences*. 2020;50(8):1801-1809. doi:10.3906/sag-2006-164  363. Guo HJ, Jiang F, Chen C, Shi JY, Zhao YW. Plasma brain natriuretic peptide, platelet parameters, and cardiopulmonary function in chronic obstructive pulmonary disease. Article. *World Journal of Clinical Cases*. 2021;9(36):11165-11172. doi:10.12998/wjcc.v9.i36.11165  364. Gupta AK, Nethan ST, Mehrotra R. Tobacco use as a well-recognized cause of severe COVID-19 manifestations. Review. *Respiratory Medicine*. 2021;176doi:10.1016/j.rmed.2020.106233  365. Gupta D, Agarwal R, Aggarwal AN, et al. Guidelines for diagnosis and management of chronic obstructive pulmonary disease: Joint ICS/NCCP (I) recommendations. Article. *Lung India*. 2013;30(3):228-267. doi:10.4103/0970-2113.116248  366. Gupta R, Agrawal R, Bukhari Z, et al. Higher comorbidities and early death in hospitalized African-American patients with Covid-19. Article. *BMC Infectious Diseases*. 2021;21(1)doi:10.1186/s12879-021-05782-9  367. Güven SC, Apaydln H, Özdemir B, et al. Antineutrophil cytoplasmic antibody positivity and clinical implications in COVID-19. Article. *Future Virology*. 2022;17(4):239-245. doi:10.3233/epl-210113  368. Häberle H, Magunia H, Lang P, et al. Mesenchymal Stem Cell Therapy for Severe COVID-19 ARDS. Article. *Journal of Intensive Care Medicine*. 2021;36(6):681-688. doi:10.1177/0885066621997365  369. Hafeez MM, Khan MT, Shakoori TA, et al. Assessment of Inflammatory Biomarkers as Predictor of Mortality in COVID-19 Patients. Article. *Pakistan Journal of Medical and Health Sciences*. 2022;16(4):343-345. doi:10.53350/pjmhs22164343  370. Haibel H, Angermair S, Schumann M, Vahldiek J, Poddubnyy D, Schneider T. Successful treatment of severe COVID-19 pneumonia with simultaneous tocilizumab and anakinra-a case series. Conference Abstract. *Arthritis and Rheumatology*. 2021;73(SUPPL 9):392-393. doi:10.1002/art.41966  371. Haibel H, Vahldiek JL, Angermair S, et al. Successful treatment of severe COVID-19 pneumonia and cytokine release with simultaneous tocilizumab and anakinra with one-month follow-up. Conference Abstract. *Annals of the Rheumatic Diseases*. 2021;80(SUPPL 1):887-888. doi:10.1136/annrheumdis-2021-eular.2289  372. Haji Datoo ASMA, Williams J, puthillath A. A RARE CASE OF OXALIPLATIN-INDUCED PULMONARY FIBROSIS. Conference Abstract. *Chest*. 2021;160(4):A759. doi:10.1016/j.chest.2021.07.717  373. Halıcı A, Hür İ, Abatay K, Çetin E, Halıcı F, Özkan S. The role of presepsin in the diagnosis of chronic obstructive pulmonary disease acute exacerbation with pneumonia. *Biomark Med*. Jan 2020;14(1):31-41. doi:10.2217/bmm-2019-0183  374. Hallcl A, Hür I, Abatay K, Çetin E, Hallcl F, Özkan S. The role of presepsin in the diagnosis of chronic obstructive pulmonary disease acute exacerbation with pneumonia. Article. *Biomarkers in Medicine*. 2019;14(1):31-41. doi:10.2217/bmm-2019-0183  375. Hamade B, Huang DT. Procalcitonin: Where Are We Now? Review. *Critical Care Clinics*. 2020;36(1):23-40. doi:10.1016/j.ccc.2019.08.003  376. Hankey B, Riley B. Towards evidence based emergency medicine: Best BETs from the manchester royal infirmary. Article. *Emergency Medicine Journal*. 2015;32(6):493-495. doi:10.1136/emermed-2015-204865.1  377. Hankey B, Riley B. Towards evidence based emergency medicine: best BETs from the Manchester Royal Infirmary. BET 1: use of a procalcitonin algorithm to guide antimicrobial therapy in COPD exacerbations can reduce antibiotic consumption with no increase in rates of treatment failure or mortality. *Emergency medicine journal : EMJ*. Jun 2015;32(6):493-5. doi:10.1136/emermed-2015-204865.1  378. Hasani H, Mardi S, Shakerian S, Taherzadeh-Ghahfarokhi N, Mardi P. The Novel Coronavirus Disease (COVID-19): A PRISMA Systematic Review and Meta-Analysis of Clinical and Paraclinical Characteristics. Review. *BioMed Research International*. 2020;2020doi:10.1155/2020/3149020  379. Hassan AE. An observational cohort study to assess N-acetylglucosamine for COVID-19 treatment in the inpatient setting. Article. *Annals of Medicine and Surgery*. 2021;68doi:10.1016/j.amsu.2021.102574  380. Haubitz S, Mueller B, Schuetz P. Streamlining antibiotic therapy with procalcitonin protocols: Consensus and controversies. Review. *Expert Review of Respiratory Medicine*. 2013;7(2):145-157. doi:10.1586/ers.13.6  381. He BC, Liu LL, Chen BL, Zhang F, Su X. The application of next-generation sequencing in diagnosing invasive pulmonary aspergillosis: Three case reports. Article. *American Journal of Translational Research*. 2019;11(4):2532-2539.  382. He J, Zhang B, Zhou Q, et al. The prognostic value of myocardial injury in COVID-19 patients and associated characteristics. Article. *Immunity, inflammation and disease*. 2021;9(4):1358-1369. doi:10.1002/iid3.484  383. He J, Zhang B, Zhou Q, et al. The Prognostic Value of Myocardial Injury in COVID-19 Patients and Associated Characteristics. *Research square*. Feb 19 2021;doi:10.21203/rs.3.rs-251810/v1  384. He Y, Xie M, Zhao J, Liu X. Clinical characteristics and outcomes of patients with severe COVID-19 and chronic obstructive pulmonary disease (COPD). Article. *Medical Science Monitor*. 2020;26doi:10.12659/MSM.927212  385. Hehar J, Todter E, Lahiri SW. Association of Patient Characteristics, Diabetes, BMI, and Obesity With Severe COVID-19 in Metropolitan Detroit, MI. Article. *Clinical Diabetes*. 2022;40(2):141-152. doi:10.2337/cd21-0065  386. Heilmann E, Gregoriano C, Annane D, et al. Duration of antibiotic treatment using procalcitonin-guided treatment algorithms in older patients: A patient-level meta-analysis from randomized controlled trials. Review. *Age and Ageing*. 2021;50(5):1546-1556. doi:10.1093/ageing/afab078  387. Hellwig B. Respiratory tract infection: Procalcitonin as biomarker in practice guidelines. Short Survey. *Deutsche Apotheker Zeitung*. 2009;149(45):50.  388. Hermel DJ, Spierling Bagsic SR, Costantini CL, Mason JR, Gahvari ZJ, Saven A. ABO phenotype and clinical correlates of COVID-19 severity in hospitalized patients. Article. *Future Science OA*. 2021;7(8)doi:10.2144/fsoa-2021-0045  389. Hey J, Thompson-Leduc P, Kirson NY, et al. Procalcitonin guidance in patients with lower respiratory tract infections: A systematic review and meta-analysis. Review. *Clinical Chemistry and Laboratory Medicine*. 2018;56(8):1200-1209. doi:10.1515/cclm-2018-0126  390. Hirst T. Procalcitonin to guide antimicrobial stewardship in the ICU: Are we consistent in our approach? Conference Abstract. *Journal of the Intensive Care Society*. 2020;21(2 SUPPL):147-148. doi:10.1177/1751143720915029  391. Hlapčić I, Somborac-Bačura A, Popović-Grle S, et al. Platelet indices in stable chronic obstructive pulmonary disease – Association with inflammatory markers, comorbidities and therapy. Article. *Biochemia Medica*. 2020;30(1)doi:10.11613/BM.2020.010701  392. Hlapčić I, Somborac-Bačura A, Popović-Grle S, et al. Platelet indices in stable chronic obstructive pulmonary disease - association with inflammatory markers, comorbidities and therapy. *Biochem Med (Zagreb)*. Feb 15 2020;30(1):010701. doi:10.11613/bm.2020.010701  393. Hnamte L, Saroha A, Gogoi P, Diwaker P. Citrullinated Histone H3 as a Marker of Neutrophil Extracellular Trap (NET) Formationin Stable and Acute Exacerbation of Chronic Obstructive Pulmonary Disease (COPD). Conference Abstract. *Indian Journal of Hematology and Blood Transfusion*. 2021;37(SUPPL 1):S83-S84. doi:10.1007/s12288-021-01510-0  394. Ho KS, Pattupara A, Herrera Y, Poon J, Poon J, Steiger D. THE MOUNT SINAI COVID-19 EXPERIENCE: 4,300 HOSPITALIZED PATIENTS IN NEW YORK CITY. Conference Abstract. *Chest*. 2020;158(4):A321. doi:10.1016/j.chest.2020.08.318  395. Ho YN, D'Souza JM, Seneviratna A, Lew SJW, Arputhan AJ, Puah SH. Single-centre retrospective study of admissions to the medical intensive care unit (MICU) and the utility of lactate procalcitonin product in predicting mortality. Conference Abstract. *Respirology*. 2018;23:146-147. doi:10.1111/resp.13420_155  396. Holt A, Sinclair S. NONTYPEABLE HAEMOPHILUS INFLUENZAE VENTRICULITIS COMPLICATED BY HYDROCEPHALUS IN A PATIENT WITH SICKLE CELL DISEASE. Conference Abstract. *Chest*. 2020;158(4):A740. doi:10.1016/j.chest.2020.08.693  397. Hongrui Z, Songping L, Lei L, Desen D, Baomin D. Clinical study on optimal switching mode in sequential noninvasive-invasive mechanical ventilation for acute exacerbation of chronic obstructive pulmonary disease. Article. *Zhonghua Wei Zhong Bing Ji Jiu Yi Xue*. 2020;32(2):161-165. doi:10.3760/cma.j.cn121430-20191224-00030  398. Horodinschi RN, Bratu OG, Dediu GN, Pantea Stoian A, Motofei I, Diaconu CC. Heart failure and chronic obstructive pulmonary disease: a review. Review. *Acta Cardiologica*. 2020;75(2):97-104. doi:10.1080/00015385.2018.1559485  399. Hoult G, Gillespie D, Wilkinson TMA, Thomas M, Francis NA. Biomarkers to guide the use of antibiotics for acute exacerbations of COPD (AECOPD): a systematic review and meta-analysis. Article. *BMC Pulmonary Medicine*. 2022;22(1)doi:10.1186/s12890-022-01958-4  400. Housset B. Rising to the challenge of resistance: a case study-based discussion. Article. *International Journal of Antimicrobial Agents*. 2007;29(SUPPL. 1):S11-S16. doi:10.1016/S0924-8579(07)70005-5  401. Hricz C. Procalcitonin test: New tool to guide antibiotics for lower respiratory infections and sepsis. Article. *Consultant*. 2018;58(5)  402. Hsu TYT, D'Silva KM, Patel NJ, et al. Laboratory trends, hyperinflammation, and clinical outcomes for patients with a systemic rheumatic disease admitted to hospital for COVID-19: a retrospective, comparative cohort study. Article. *The Lancet Rheumatology*. 2021;3(9):e638-e647. doi:10.1016/S2665-9913(21)00140-5  403. Hu C, Zeng JP, Peng K, et al. Clinical features and temporal lung radiographic changes in 25 patients recovering from COVID-19 Pneumonia: A retrospective case-control study. Article. *Medical Science Monitor*. 2021;27doi:10.12659/MSM.933381  404. Hu XS, Hu CH, Zhong P, Wen YJ, Chen XY. Risk factors associated with acute respiratory distress syndrome in COVID-19 patients outside Wuhan: A double-center retrospective cohort study of 197 cases in Hunan, China. Article. *World Journal of Clinical Cases*. 2021;9(2):344-356. doi:10.12998/wjcc.v9.i2.344  405. Hu Y, Deng H, Huang L, Xia L, Zhou X. Analysis of Characteristics in Death Patients with COVID-19 Pneumonia without Underlying Diseases. Letter. *Academic Radiology*. 2020;27(5):752. doi:10.1016/j.acra.2020.03.023  406. Hua M, Liu J, Du P, et al. The novel outer membrane protein from OprD/Occ family is associated with hypervirulence of carbapenem resistant Acinetobacter baumannii ST2/KL22. Article. *Virulence*. 2021;12(1):1-11. doi:10.1080/21505594.2020.1856560  407. Huang D, Mu B, Mu W. Expression and correlation of serum strem-1, CTRP5 and SST2 in chronic obstructive pulmonary disease. Article. *Acta Medica Mediterranea*. 2021;37(6):3165-3170. doi:10.19193/0393-6384_2021_6_498  408. Huang DT, Yealy DM, Filbin MR, et al. Procalcitonin-guided use of antibiotics for lower respiratory tract infection. Article. *New England Journal of Medicine*. 2018;379(3):236-249. doi:10.1056/NEJMoa1802670  409. Huang H, Li H, Chen S, et al. Prevalence and Characteristics of Hypoxic Hepatitis in COVID-19 Patients in the Intensive Care Unit: A First Retrospective Study. Article. *Frontiers in medicine*. 2020;7doi:10.3389/fmed.2020.607206  410. Huang L, Wang J, Gu X, Sheng W, Wang Y, Cao B. Procalcitonin-guided initiation of antibiotics in AECOPD inpatients: Study protocol for a multicenter randomised controlled trial. Journal: Article. *BMJ Open*. 2021;11(8)doi:10.1136/bmjopen-2021-049515  411. Huang X, Zhao M. High expression of long non-coding RNA MALAT1 correlates with raised acute respiratory distress syndrome risk, disease severity, and increased mortality in sepstic patients. *International journal of clinical and experimental pathology*. 2019;12(5):1877-1887.  412. Huang Y, Tu M, Wang S, et al. Clinical characteristics of laboratory confirmed positive cases of SARS-CoV-2 infection in Wuhan, China: A retrospective single center analysis. Article. *Travel Medicine and Infectious Disease*. 2020;36doi:10.1016/j.tmaid.2020.101606  413. Huerta A, Crisafulli E, Menendez R, et al. Acute exacerbations of COPD and COPD patients with community-acquired pneumonia: Characteristics, systemic inflammatory response and outcomes. Conference Abstract. *American Journal of Respiratory and Critical Care Medicine*. 2013;187  414. Huerta A, Crisafulli E, Menéndez R, et al. Pneumonic and nonpneumonic exacerbations of COPD: Inflammatory response and clinical characteristics. Article. *Chest*. 2013;144(4):1134-1142. doi:10.1378/chest.13-0488  415. Huerta A, Domingo R, Soler N. Chronic obstructive pulmonary disease and pneumonia. Article. *Archivos de Bronconeumologia*. 2010;46(SUPPL. 3):28-31. doi:10.1016/S0300-2896(10)70024-7  416. Hunold KM, Schwaderer AL, Exline M, et al. Diagnosing Dyspneic Older Adult Emergency Department Patients: A Pilot Study. Letter. *Academic Emergency Medicine*. 2021;28(6):675-678. doi:10.1111/acem.14183  417. Hurst JR. Bronchial and systemic inflammation in chronic obstructive pulmonary disease. 2010. p. 19-22.  418. Hurst JR. Exacerbation phenotyping in chronic obstructive pulmonary disease. Editorial. *American Journal of Respiratory and Critical Care Medicine*. 2011;184(6):625-626. doi:10.1164/rccm.201106-1136ED  419. Hurst JR, Wedzicha JA. Management and prevention of chronic obstructive pulmonary disease exacerbations: A state of the art review. Review. *BMC Medicine*. 2009;7doi:10.1186/1741-7015-7-40  420. Hwang HJ, Kang SW. Coronary Sinus Endocarditis in a Hemodialysis Patient: A Case Report and Review of Literature. Article. *World Journal of Clinical Cases*. 2021;9(17):4348-4356. doi:10.12998/wjcc.v9.i17.4348  421. Iglesias J, Vassallo AV, Liesenfeld O, et al. A 33-mrna classifier is able to produce inflammopathic, adaptive, and coagulopathic endotypes with prognostic significance: the outcomes of metabolic resuscitation using ascorbic acid, thiamine, and glucocorticoids in the early treatment of sepsis (Oranges) trial. Article. *Journal of Personalized Medicine*. 2021;11(1):1-14. doi:10.3390/jpm11010009  422. Ignatova GL, Antonov VN. [Impact of vaccination on the course of bronchial and systemic inflammation in patients with COPD and CHD]. *Ter Arkh*. 2017;89(3):29-33. Vliyanie vaktsinatsii na dinamiku bronkhial'nogo i sistemnogo vospaleniya u patsientov s khronicheskoi obstruktivnoi bolezn'yu legkikh i ishemicheskoi bolezn'yu serdtsa. doi:10.17116/terarkh201789329-33  423. Iliaz R, Iliaz S. The neutrophil–lymphocyte ratio as an inflammatory marker in acute exacerbation of COPD. Letter. *Wiener Klinische Wochenschrift*. 2016;128(17-18):672-673. doi:10.1007/s00508-016-1022-9  424. Ilonzo N, Rao A, Safir S, et al. Acute thrombotic manifestations of coronavirus disease 2019 infection: Experience at a large New York City health care system. Article. *Journal of Vascular Surgery*. 2021;73(3):789-796. doi:10.1016/j.jvs.2020.08.038  425. Irct138904144312N. Effect of Atrovastatin on lung inflammation in bronchitis. *<https://trialsearchwhoint/Trial2aspx?TrialID=IRCT138904144312N1>*. 2014;  426. Irct2017010331737N. Use of Procalcitonin in therapy of Pneumonia. *<https://trialsearchwhoint/Trial2aspx?TrialID=IRCT2017010331737N1>*. 2017;  427. Ishiguro T, Kojima A, Shimizu T, Mita N, Kuroiwa S, Takayanagi N. Combined hemophagocytic syndrome and thrombotic microangiopathy due to mixed infection with influenza virus and pneumococcal pneumonia. Article. *Clinical Case Reports*. 2019;7(1):131-134. doi:10.1002/ccr3.1842  428. Ismail NA, Abdallah AL, Esawy MM, Shabana MA. Infection index: a promising additive tool in detecting bacterial colonization. Article. *Egyptian Journal of Chest Diseases and Tuberculosis*. 2021;70(4):474-480. doi:10.4103/ejcdt.ejcdt-31-20  429. Isrctn. Procalcitonin-guided antibiotic therapy in acute exacerbations of chronic obstructive pulmonary disease (COPD) (AECOPD): a randomised trial - The ProCOLD Study. *<https://trialsearchwhoint/Trial2aspx?TrialID=ISRCTN77261143>*. 2005;  430. Isrctn. Randomised controlled trial of a home-based exercise programme for patients with chronic obstructive pulmonary disease. *<https://trialsearchwhoint/Trial2aspx?TrialID=ISRCTN61732734>*. 2007;  431. Isrctn. Pursed Lip Breathing (PLB) and its role in the management of breathlessness in stable chronic obstructive pulmonary disease (COPD). *<https://trialsearchwhoint/Trial2aspx?TrialID=ISRCTN83029560>*. 2007;  432. Isrctn. Fast multiplex assay of Gram-negative rods for antibiotic stewardship in hospitalized patients. *<https://trialsearchwhoint/Trial2aspx?TrialID=ISRCTN95828556>*. 2017;  433. Isrctn. A controlled clinical trial to evaluate a personalised treatment approach for the management of acute exacerbations (flare-ups) of chronic obstructive pulmonary disease (COPD). *<https://trialsearchwhoint/Trial2aspx?TrialID=ISRCTN85620156>*. 2019;  434. Isrctn. Evaluating the usefulness of CT-scans in the diagnosis and management of chronic obstructive pulmonary disease (COPD) patients with respiratory symptoms. *<https://trialsearchwhoint/Trial2aspx?TrialID=ISRCTN39235677>*. 2020;  435. Izcovich A, Ragusa MA, Tortosa F, et al. Prognostic factors for severity and mortality in patients infected with COVID-19: A systematic review. *PLoS One*. 2020;15(11):e0241955. doi:10.1371/journal.pone.0241955  436. Izcovich A, Ragusa MA, Tortosa F, et al. Prognostic factors for severity and mortality in patients infected with COVID-19: A systematic review. Article. *PLoS ONE*. 2020;15(11 November)doi:10.1371/journal.pone.0241955  437. Izquierdo Alonso JL. The Future of Biological Markers in COPD. Article. *Archivos de Bronconeumologia*. 2017;53(10):541-542. doi:10.1016/j.arbr.2017.04.016  438. Jagannathan SH, Winn CM, Nayar AP, Koussa GJ, Brenner CA. Sarcoidosis with secondary recurrent right-sided chylothorax and chylous ascites in a Caucasian male patient. Article. *Oxford Medical Case Reports*. 2021;2021(10):399-402. doi:10.1093/omcr/omab098  439. Jain A, Aggarwal A, Kumar S. An unusual cause of diffuse pulmonary hemorrhage. Conference Abstract. *American Journal of Respiratory and Critical Care Medicine*. 2019;199(9)  440. James M, Stokes E. Spending by primary care practices - does it show what we expect? Article. *The International journal of health planning and management*. 2014;29(3):244-259.  441. Jamieson L, Thornton H, Harries T, Schofield P, Jackson K, White P. COPD admissions in London: What general practice characteristics are associated? Conference Abstract. *European Respiratory Journal*. 2013;42  442. Jarvis S, Shiner RJ, Thomas CP, et al. Microbial contamination of home nebulisers used by COPD patients. Conference Abstract. *Thorax*. 2009;64:A169. doi:10.1136/thx.2009.127654g  443. Jayaprakash K, Sharmila Nirojini P, Johny N, et al. A comparative study to assess the rates on the recovery of covid-19 patients with or without comorbidities in a tertiary care hospital. Article. *International Journal of Pharmaceutical Sciences Review and Research*. 2021;70(2):188-195. doi:10.47583/ijpsrr.2021.v70i02.023  444. Jeanrenaud P, Hammell C, Dempsey GA, et al. Markers of bacterial infection in the critically ill: A comparison of procalcitonin, C reactive protein and the neutrophil band count. Letter. *Journal of Infection*. 2012;64(5):540-542. doi:10.1016/j.jinf.2012.01.011  445. Jesús Cremades M, Luiza de Souza-Galvão M, García JM, Menéndez R. Respiratory Infections Research: a Perspective From the Tuberculosis and Respiratory Infections Area (TIR). Article. *Archivos de Bronconeumologia*. 2009;45(SUPPL. 1):11-15. doi:10.1016/S0300-2896(09)70265-0  446. Ji X, Cui W, Zhang B, Shan S. Effect of lung protective ventilation on perioperative pulmonary infection in elderly patients with mild to moderate COPD under general anesthesia. Article. *Journal of Infection and Public Health*. 2020;13(2):281-286. doi:10.1016/j.jiph.2019.11.021  447. Jia TG, Zhao JQ, Liu JH. Serum inflammatory factor and cytokines in AECOPD. Article. *Asian Pacific Journal of Tropical Medicine*. 2014;7(12):1005-1008. doi:10.1016/S1995-7645(14)60177-2  448. Jia X, Li C, Cao J, Wu X, Zhang L. Clinical characteristics and predictors of mortality in patients with candidemia: a six-year retrospective study. Article. *European Journal of Clinical Microbiology and Infectious Diseases*. 2018;37(9):1717-1724. doi:10.1007/s10096-018-3304-9  449. Jiang M, Li C, Zheng L, et al. A biomarker-based age, biomarkers, clinical history, sex (ABCS)-mortality risk score for patients with coronavirus disease 2019. Article. *Annals of translational medicine*. 2021;9(3)doi:10.21037/atm-20-6205  450. Jiang M, Liu L, Yan L, Liu C. Risk factors for infections from multidrug-resistant organisms in patients with chronic obstructive pulmonary disease and pulmonary infections and the related nursing interventions. Article. *International Journal of Clinical and Experimental Medicine*. 2020;13(12):9923-9931.  451. Jiang Y, Abudurexiti S, An MM, Cao D, Wei J, Gong P. Risk factors associated with 28-day all-cause mortality in older severe COVID-19 patients in Wuhan, China: a retrospective observational study. Article. *Scientific reports*. 2020;10(1):22369. doi:10.1038/s41598-020-79508-3  452. Jin J, Zhang H, Li D, et al. Effectiveness of Xin Jia Xuan Bai Cheng Qi Decoction in treating acute exacerbation of chronic obstructive pulmonary disease: Study protocol for a multicentre, randomised, controlled trial. Article. *BMJ Open*. 2019;9(11)doi:10.1136/bmjopen-2019-030249  453. Jin M, Lu Z, Zhang X, et al. Clinical characteristics and risk factors of fatal patients with COVID-19: a retrospective cohort study in Wuhan, China. Article. *BMC Infectious Diseases*. 2021;21(1)doi:10.1186/s12879-021-06585-8  454. Johnsen S, Sattler SM, Miskowiak KW, et al. Descriptive analysis of long COVID sequelae identified in a multidisciplinary clinic serving hospitalised and non-hospitalised patients. Article. *ERJ Open Research*. 2021;7(3)doi:10.1183/23120541.00205-2021  455. Jongepier L, Crocker C, Hawkins P. Changes in caseload and admission avoidance in patients with COPD - The north east essex experience. Conference Abstract. *COPD: Journal of Chronic Obstructive Pulmonary Disease*. 2011;8(1):43. doi:10.3109/15412555.2011.541141  456. Jongepier L, Hawkins P. <Www.effectiveness> and patient satisfaction of a new multi site community pulmonary rehabilitation programme. Conference Abstract. *COPD: Journal of Chronic Obstructive Pulmonary Disease*. 2011;8(1):42. doi:10.3109/15412555.2011.541141  457. Joseph P, Godofsky E. Outpatient antibiotic stewardship: A growing frontier-combining myxovirus resistance protein a with other biomarkers to improve antibiotic use. Article. *Open Forum Infectious Diseases*. 2018;5(2)doi:10.1093/ofid/ofy024  458. Joshi M, Varkey B. Editorial: Reflections on chronic obstructive pulmonary disease and related topics and respiratory health consequences of wildfires. Editorial. *Current Opinion in Pulmonary Medicine*. 2019;25(2):129-131. doi:10.1097/MCP.0000000000000563  459. Jothimani D, Morgan N, Snape J. Hypertension associated with hypokalaemia and hyponatraemia. Article. *CME Journal Geriatric Medicine*. 2005;7(2):106-107.  460. Joyce K, Bowman T. Necrotizing pneumonia caused by streptococcus constellatus in a male patient with poor dentition. Conference Abstract. *Journal of General Internal Medicine*. 2021;36(SUPPL 1):S342. doi:10.1007/s11606-021-06830-5  461. Junarta J, Riley JM, Pavri BB. Describing heart rate variability in patients with chronic atrial fibrillation during hospitalization for COVID-19. Article. *Journal of Arrhythmia*. 2021;37(4):893-898. doi:10.1002/joa3.12569  462. Kaeslin M, Brunner S, Raths J, Huber A. Improvement in detecting bacterial infection in lower respiratory tract infections using the Intensive Care Infection Score (ICIS). Article. *LaboratoriumsMedizin*. 2016;40(3):175-182. doi:10.1515/labmed-2016-0021  463. Kalil AC, Lisboa T. To Procalcitonin, or Not to Procalcitonin? Editorial. *Chest*. 2019;155(6):1085-1087. doi:10.1016/j.chest.2019.02.327  464. Kamat IS, Ramachandran V, Eswaran H, Abers MS, Musher DM. Low procalcitonin, community acquired pneumonia, and antibiotic therapy. Letter. *The Lancet Infectious Diseases*. 2018;18(5):496-497. doi:10.1016/S1473-3099(18)30215-9  465. Kapur N, Mackay IM, Masters IB, Sloots TP, Chang AB. Role of upper respiratory viruses in exacerbations of non-cystic fibrosis bronchiectasis in children. Conference Abstract. *Respirology*. 2011;16:71. doi:10.1111/j.1440-1843.2011.01937.x  466. Kapur N, Mackay IM, Sloots TP, Masters IB, Chang AB. Respiratory viruses in exacerbations of non-cystic fibrosis bronchiectasis in children. *Arch Dis Child*. Aug 2014;99(8):749-53. doi:10.1136/archdischild-2013-305147  467. Karabulut D, Oflar E. Comparative performance of CHA2DS2VASc and anticoagulation and risk factors in atrial fibrillation risk scores for predicting mortality in patients with COVID-19. Article. *International Journal of the Cardiovascular Academy*. 2021;7(2):33-38. doi:10.4103/ijca.ijca_57_20  468. Karimian M, Mansouri F, Borji M, et al. Prevention, diagnosis, and treatment of covid-19 in infants and children: A systematic review study of performed protocols. Review. *Archives of Clinical Infectious Diseases*. 2020;15(6)doi:10.5812/archcid.103180  469. Katz-Greenberg G, Yadav A, Gupta M, et al. Outcomes of COVID-19-positive kidney transplant recipients: A single-center experience. Letter. *Clinical Nephrology*. 2020;94(6):318-321. doi:10.5414/CN110311  470. Kaya F, Konya PŞ, Demirel E, Demirtürk N, Orhan S, Ufuk F. Visual and quantitative assessment of covid-19 pneumonia on chest ct: The relationship with disease severity and clinical findings. Article. *Current Medical Imaging*. 2021;17(9):1142-1150. doi:10.2174/1573405617666210215142528  471. Kazancioglu S, Yilmaz FM, Bastug A, et al. Assessment of galectin-1, galectin-3, and prostaglandin e2 levels in patients with covid-19. Article. *Japanese Journal of Infectious Diseases*. 2021;74(6):530-536. doi:10.7883/yoken.JJID.2021.020  472. Kearney M, Moger A, Clarke J, Holton K, Winter R, Hill S. The NHS atlas of variation in respiratory healthcare: Using evidence of variation to drive improvement in outcomes. Conference Abstract. *American Journal of Respiratory and Critical Care Medicine*. 2013;187  473. Keenan J. The European Respiratory Society Congress Munich, Germany September 2-6, 2006. Conference Paper. *Annals of Long-Term Care*. 2007;15(1):28-32.  474. Keenan J. Annual Meeting of the American Thoracic Society Toronto, Canada, May 16-21, 2008. Conference Paper. *Annals of Long-Term Care*. 2008;16(10):16-18.  475. Keenan JM. Annual Meeting of the American Thoracic Society May 16-21, 2008 Toronto, Canada. Conference Paper. *Clinical Geriatrics*. 2008;16(10):22-24.  476. Keitel V, Bode JG, Feldt T, et al. Case Report: Convalescent Plasma Achieves SARS-CoV-2 Viral Clearance in a Patient With Persistently High Viral Replication Over 8 Weeks Due to Severe Combined Immunodeficiency (SCID) and Graft Failure. Article. *Frontiers in Immunology*. 2021;12doi:10.3389/fimmu.2021.645989  477. Kettner J, Holek M, Franekova J, et al. Procalcitonin Dynamics After Long-Term Ventricular Assist Device Implantation. Article. *Heart Lung and Circulation*. 2017;26(6):599-603. doi:10.1016/j.hlc.2016.09.014  478. Kewalram G, Masel P, Mercer M, et al. Pneumonia in the elderly may cause deterioration in functional levels three months after the illness. Conference Abstract. *Respirology*. 2014;19:44. doi:10.1111/resp.12262  479. Khan D, Shadi M, Mustafa A, et al. A Wolf in Sheep's clothing; Case reports and literature review of Corynebacterium striatum endocarditis. Article. *IDCases*. 2021;24doi:10.1016/j.idcr.2021.e01070  480. Kim H, Cha SI, Shin KM, et al. Clinical relevance of bronchial anthracofibrosis in patients with chronic obstructive pulmonary disease exacerbation. Article. *Tuberculosis and Respiratory Diseases*. 2014;77(3):124-131. doi:10.4046/trd.2014.77.3.124  481. Kim V, Williams N, Ostridge K, et al. Sputum colour in the light of the health related quality of life, airways and systemic biomarkers in exacerbations of COPD. Conference Abstract. *Thorax*. 2014;69:A19-A20. doi:10.1136/thoraxjnl-2014-206260.39  482. King PT, MacDonald M, Bardin PG. Bacteria in COPD; their potential role and treatment. Review. *Translational Respiratory Medicine*. 2013;1(1):1-9. doi:10.1186/2213-0802-1-13  483. Kiss N, Lőrincz K, Medvecz M, et al. Coronavirus disease 2019 in a psoriatic patient with concomitant chronic obstructive pulmonary disease under treatment with risankizumab. Letter. *Dermatologic Therapy*. 2020;33(6)doi:10.1111/dth.14186  484. Kluge S, Janssens U, Welte T, et al. Recommendations for treatment of critically ill patients with COVID-19: Version 3 S1 guideline. Review. *Anaesthesist*. 2021;70:19-29. doi:10.1007/s00101-020-00879-3  485. Kocayığıt H, Özmen Süner K, Tomak Y, et al. Characteristics and outcomes of critically ill patients with covid-19 in Sakarya, Turkey: A single centre cohort study. Article. *Turkish Journal of Medical Sciences*. 2021;51(2):440-447. doi:10.3906/sag-2005-57  486. Koch A, Pizanis N, Bessa V, et al. Impact of normothermic ex vivo lung perfusion on early post-transplantation cytomegalovirus infection. Article. *Journal of Thoracic Disease*. 2020;12(4):1350-1356. doi:10.21037/jtd.2020.02.26  487. Koczulla AR, Noeske S, Herr C, et al. Alpha-1 antitrypsin is elevated in exhaled breath condensate and serum in exacerbated COPD patients. Article. *Respiratory Medicine*. 2012;106(1):120-126. doi:10.1016/j.rmed.2011.06.015  488. Kokturk N, Babayiǧit C, Kul S, et al. The predictors of COVID-19 mortality in a nationwide cohort of Turkish patients. Conference Abstract. *European Respiratory Journal*. 2021;58(SUPPL 65)doi:10.1183/13993003.congress-2021.PA3817  489. Kokturk N, Babayigit C, Kul S, et al. The predictors of COVID-19 mortality in a nationwide cohort of Turkish patients. Article. *Respiratory Medicine*. 2021;183doi:10.1016/j.rmed.2021.106433  490. Kolb MRJ, Yang I. Respirology year-in-review 2008: Basic science. Review. *Respirology*. 2009;14(3):318-326. doi:10.1111/j.1440-1843.2009.01491.x  491. Kopterides P, Tsangaris I. Procalcitonin and sepsis: Recent data on diagnostic utility prognostic potential and therapeutic implications in critically ill patients. Review. *Minerva Anestesiologica*. 2012;78(7):823-835.  492. Koraćević G, Ilić D. Should anti-vitamin K be started on the first day in non-high risk pulmonary embolism? Article. *Vojnosanitetski Pregled*. 2020;77(12):1336-1341. doi:10.2298/VSP170121001K  493. Kostikas K, Bakakos P, Papiris S, Stolz D, Celli BR. Systemic biomarkers in the evaluation and management of COPD patients: Are we getting closer to clinical application? Review. *Current Drug Targets*. 2013;14(2):177-191. doi:10.2174/1389450111314020005  494. Kotfis K, Witkiewicz W, Szylińska A, et al. Delirium severely worsens outcome in patients with covid-19—a retrospective cohort study from temporary critical care hospitals. Article. *Journal of Clinical Medicine*. 2021;10(13)doi:10.3390/jcm10132974  495. Krasselt M, Baerwald C, Petros S, Seifert O. Outcome of Patients With Necrotizing Vasculitis Admitted to the Intensive Care Unit (ICU) for Sepsis: Results of a Single-Centre Retrospective Analysis. Article. *Journal of Intensive Care Medicine*. 2021;36(12):1410-1416. doi:10.1177/0885066620953768  496. Kristoffersen KB, Søgaard OS, Wejse C, et al. Antibiotic treatment interruption of suspected lower respiratory tract infections based on a single procalcitonin measurement at hospital admission - A randomized trial. Article. *Clinical Microbiology and Infection*. 2009;15(5):481-487. doi:10.1111/j.1469-0691.2009.02709.x  497. Krüger S, Frechen D. Cardiovascular complications and comorbidities in CAP. 2014. p. 256-265.  498. Kubin CJ, McConville TH, Dietz D, et al. Characterization of Bacterial and Fungal Infections in Hospitalized Patients with Coronavirus Disease 2019 and Factors Associated with Health Care-Associated Infections. Article. *Open Forum Infectious Diseases*. 2021;8(6)doi:10.1093/ofid/ofab201  499. Kubrova E, Mughal U, Ade DG, Rapolu M, Lambl BB. Can PCT value predict outcome in COVID 19 patients. Conference Abstract. *Journal of General Internal Medicine*. 2021;36(SUPPL 1):S15. doi:10.1007/s11606-021-06830-5  500. Kukic BP, Savic NB, Stevanovic KS, Trailovic RD, Cvetkovic SD, Davidovic LB. Effect of IgM-Enriched Immunoglobulin as Adjunctive Therapy in a Patient Following Sepsis after Open Thoracoabdominal Aortic Aneurysm Repair. Article. *Journal of Cardiothoracic and Vascular Anesthesia*. 2016;30(3):746-748. doi:10.1053/j.jvca.2015.08.025  501. Kumar A, Arora A, Sharma P, et al. Clinical Features of COVID-19 and Factors Associated with Severe Clinical Course: A Systematic Review and Meta-Analysis. *Ssrn*. Apr 21 2020:3566166. doi:10.2139/ssrn.3566166  502. Kumar A, Fatima K, Bejko E. The unusual fatal presentation of cocaine levamisole induced vasculitis. Conference Abstract. *Journal of General Internal Medicine*. 2016;31(2):S759-S760.  503. Kumar V, Bhushan D, Supriya S, et al. A Comparative study of the clinical characteristics of the first and second waves of COVID-19 in a tertiary care centre of North India. Article. *European Journal of Molecular and Clinical Medicine*. 2021;8(4):2082-2094.  504. Kyriazopoulou E, Giamarellos-Bourboulis EJ. Antimicrobial Stewardship Using Biomarkers: Accumulating Evidence for the Critically Ill. Review. *Antibiotics*. 2022;11(3)doi:10.3390/antibiotics11030367  505. Kyriazopoulou E, Karageorgos A, Liaskou-Antoniou L, et al. BioFire® FilmArray® Pneumonia Panel for Severe Lower Respiratory Tract Infections: Subgroup Analysis of a Randomized Clinical Trial. Journal: Article. *Infectious Diseases and Therapy*. 2021;10(3):1437‐1449. doi:10.1007/s40121-021-00459-x  506. Lacoma A, Bas A, Tudela P, et al. Correlation of inflammatory and cardiovascular biomarkers with pneumonia severity scores. Article. *Enfermedades Infecciosas y Microbiologia Clinica*. 2014;32(3):140-146. doi:10.1016/j.eimc.2013.07.006  507. Lacoma A, Prat C, Andreo F, Domínguez J. Biomarkers in the management of COPD. Review. *European Respiratory Review*. 2009;18(112):96-104. doi:10.1183/09059180.00000609  508. Lacoma A, Prat C, Tudela P, et al. Pro-adrenomedullin, procalcitonin and CRP levels to predict bacterial pneumonia in patients admitted to emergency room. Conference Abstract. *European Respiratory Journal*. 2011;38  509. Lagi F, Piccica M, Graziani L, et al. Early experience of an infectious and tropical diseases unit during the coronavirus disease (COVID-19) pandemic, Florence, Italy, February to March 2020. Article. *Eurosurveillance*. 2020;25(17)doi:10.2807/1560-7917.ES.2020.25.17.2000556  510. Lai C, Yu R, Wang M, et al. Shorter incubation period is associated with severe disease progression in patients with COVID-19. Article. *Virulence*. 2020;11(1):1443-1452. doi:10.1080/21505594.2020.1836894  511. Lambers C, Qi Y, Eleni P, et al. Extracellular matrix composition is modified by β2-agonists through cAMP in COPD. Article. *Biochemical Pharmacology*. 2014;91(3):400-408. doi:10.1016/j.bcp.2014.07.026  512. Lambers C, Qi Y, Eleni P, et al. Extracellular matrix composition is modified by β₂-agonists through cAMP in COPD. *Biochem Pharmacol*. Oct 1 2014;91(3):400-8. doi:10.1016/j.bcp.2014.07.026  513. Lange NE, Sparrow D, Vokonas P, Litonjua AA. Vitamin D deficiency and obstructive lung disease in the normative aging study. Conference Abstract. *American Journal of Respiratory and Critical Care Medicine*. 2010;181(1)  514. Langley-Johnson CA, Jenkin E, Dyer CAE, et al. Facilitation of continued exercise via patient volunteers with chronic obstructive pulmonary disease (COPD) following a pulmonary rehabilitation programme: A feasibility study. Conference Abstract. *Thorax*. 2010;65:A98. doi:10.1136/thx.2010.150979.1  515. Laudisio A, Nenna A, Musarò M, et al. Perioperative management after elective cardiac surgery: The predictive value of procalcitonin for infective and noninfective complications. Article. *Future Cardiology*. 2021;17(8):1349-1358. doi:10.2217/fca-2020-0245  516. Laue J, Reierth E, Melbye H. When should acute exacerbations of copd be treated with systemic corticosteroids and antibiotics in primary care: A systematic review of current copd guidelines. Article. *Primary Care Respiratory Medicine*. 2015;25:1-8. doi:10.1038/npjpcrm.2015.2  517. Lawrence KL, Kollef MH. Antimicrobial stewardship in the Intensive care unit advances and obstacles. Review. *American Journal of Respiratory and Critical Care Medicine*. 2009;179(6):434-438. doi:10.1164/rccm.200809-1394CP  518. Layios N, Lambermont B, Canivet JL, et al. Procalcitonin usefulness for the initiation of antibiotic treatment in intensive care unit patients. Article. *Critical Care Medicine*. 2012;40(8):2304-2309. doi:10.1097/CCM.0b013e318251517a  519. Lee JR, Silberzweig J, Akchurin O, et al. Characteristics of acute kidney injury in hospitalized COVID-19 patients in an urban academic medical center. Letter. *Clinical Journal of the American Society of Nephrology*. 2021;16(2):284-286. doi:10.2215/CJN.07440520  520. Lee JX, Chieng WK, Lau SCD, Tan CE. COVID-19 and Hemoglobinopathies: A Systematic Review of Clinical Presentations, Investigations, and Outcomes. Review. *Frontiers in medicine*. 2021;8doi:10.3389/fmed.2021.757510  521. Lee JY, Hong SW, Hyun M, et al. Epidemiological and clinical characteristics of coronavirus disease 2019 in Daegu, South Korea. Article. *International Journal of Infectious Diseases*. 2020;98:462-466. doi:10.1016/j.ijid.2020.07.017  522. Leone M, Bouadma L, Bouhemad B, et al. Hospital-acquired pneumonia in ICU. Article. *Anaesthesia Critical Care and Pain Medicine*. 2018;37(1):83-98. doi:10.1016/j.accpm.2017.11.006  523. Leoni MLG, Moschini E, Beretta M, Zanello M, Nolli M. The modified NUTRIC score (mNUTRIC) is associated with increased 28-day mortality in critically ill COVID-19 patients: Internal validation of a prediction model. Article. *Clinical Nutrition ESPEN*. 2022;48:202-209. doi:10.1016/j.clnesp.2022.02.014  524. Li J, He X, Yuan Y, et al. Meta-analysis investigating the relationship between clinical features, outcomes, and severity of severe acute respiratory syndrome coronavirus 2 (SARS-CoV-2) pneumonia. Review. *American Journal of Infection Control*. 2021;49(1):82-89. doi:10.1016/j.ajic.2020.06.008  525. Li J, Zong D, Chen Y, Chen P. Anti-apoptotic effect of the Shh signaling pathway in cigarette smoke extract induced MLE 12 apoptosis. Article. *Tobacco Induced Diseases*. 2019;17(June)doi:10.18332/tid/109753  526. Li JY, Yao RQ, Liu SQ, Zhang YF, Yao YM, Tian YP. Efficiency of Monocyte/High-Density Lipoprotein Cholesterol Ratio Combined With Neutrophil/Lymphocyte Ratio in Predicting 28-Day Mortality in Patients With Sepsis. Article. *Frontiers in medicine*. 2021;8doi:10.3389/fmed.2021.741015  527. Li P, Cai H, Wang Y, Wang L, He J, Dong Y. Temporal changes in the chest based on findings from imaging in severe patients with novel coronavirus pneumonia. Article. *Molecular Medicine Reports*. 2021;23(6)doi:10.3892/mmr.2021.12081  528. Li S, Chen M, Zheng Y, Liu Z, Zeng R. Custom-made fenestrated stent for mycotic aortic aneurysms: a report of two cases. Article. *BMC Cardiovascular Disorders*. 2021;21(1)doi:10.1186/s12872-021-02234-9  529. Li S, Zhu X, Wang D, Li Y. Enteral Nutrition Improves Diaphragmatic Thickness and Prognosis of Mechanically Ventilated Patients with Chronic Obstructive Pulmonary Disease. Article. *Current Topics in Nutraceutical Research*. 2021;19(3):333-338. doi:10.37290/ctnr2641-452X.19:333-338  530. Li T, Gao L, Ma HX, et al. Clinical value of IL-13 and ECP in the serum and sputum of eosinophilic AECOPD patients. Article. *Experimental Biology and Medicine*. 2020;245(14):1290-1298. doi:10.1177/1535370220931765  531. Li X, Wang L, Yan S, et al. Clinical characteristics of 25 death cases with COVID-19: A retrospective review of medical records in a single medical center, Wuhan, China. Article. *International Journal of Infectious Diseases*. 2020;94:128-132. doi:10.1016/j.ijid.2020.03.053  532. Li Y, Li H, Song C, et al. Early Prediction of Disease Progression in Patients with Severe COVID-19 Using C-Reactive Protein to Albumin Ratio. Article. *Disease markers*. 2021;2021doi:10.1155/2021/6304189  533. Li Y, Zhu C, Zhang B, et al. Nutritional status is closely related to the severity of COVID-19: A multi-center retrospective study. Article. *Journal of Infection in Developing Countries*. 2021;15(4):490-500. doi:10.3855/JIDC.14178  534. Li YF, Li J. Clinical study on jingzhi kesou tanchuan pills combined with compound ipratropium bromide solution in treatment of acute exacerbation of chronic obstructive pulmonary disease. *Drugs and clinic*. 2019;34:2370‐2375.  535. Li Z, Yuan X, Yu L, Wang B, Gao F, Ma J. Procalcitonin-guided antibiotic therapy in acute exacerbation of chronic obstructive pulmonary disease: An updated meta-analysis. *Medicine (Baltimore)*. Aug 2019;98(32):e16775. doi:10.1097/md.0000000000016775  536. Li Z, Yuan X, Yu L, et al. Procalcitonin-guided antibiotic therapy in acute exacerbation of chronic obstructive pulmonary disease: An updated meta-analysis. Article. *Medicine (United States)*. 2019;98(32)doi:10.1097/MD.0000000000016775  537. Liddell F, Webber J. Pulmonary rehabilitation for chronic obstructive pulmonary disease: A pilot study evaluating a once-weekly versus twice-weekly supervised programme. Article. *Physiotherapy*. 2010;96(1):68-74. doi:10.1016/j.physio.2009.04.007  538. Lieveld AWE, Kok B, Schuit FH, et al. Diagnosing covid-19 pneumonia in a pandemic setting: Lung ultrasound versus ct (luvct) – a multicentre, prospective, observational study. Article. *ERJ Open Research*. 2020;6(4)doi:10.1183/23120541.00539-2020  539. Liguori G, Pavan N, Rinaldi A, et al. Fournier's gangrene: Prognostic aspects and predisposing risk factors. Conference Abstract. *European Urology, Supplements*. 2013;12(1):e614.  540. Lim HY, Abisheganaden JA, Lim AYH. Serum predictors for outcome of hospitalisation during exacerbation of bronchiectasis in adults. Conference Abstract. *Annals of the Academy of Medicine Singapore*. 2015;44(10):S157.  541. Lin C, Pang Q. Meta-analysis and systematic review of procalcitonin-guided treatment in acute exacerbation of chronic obstructive pulmonary disease. Review. *Clinical Respiratory Journal*. 2018;12(1):10-15. doi:10.1111/crj.12519  542. Lin K, Dempsey C, Patel S, Butler J, Septimus E. Evaluating the impact of procalcitonin on antibiotic utilization in chronic obstructive pulmonary disease exacerbations. Conference Abstract. *Open Forum Infectious Diseases*. 2018;5:S457-S458. doi:10.1093/ofid/ofy210.1308  543. Lindenauer PK, Shieh MS, Stefan MS, et al. Hospital procalcitonin testing and antibiotic treatment of patients admitted for chronic obstructive pulmonary disease exacerbation. Article. *Annals of the American Thoracic Society*. 2017;14(12):1779-1785. doi:10.1513/AnnalsATS.201702-133OC  544. Linet MS, Gridley G, Nyrén O, et al. Primary liver cancer, other malignancies, and mortality risks following porphyria: A cohort study in Denmark and Sweden. Article. *American Journal of Epidemiology*. 1999;149(11):1010-1015. doi:10.1093/oxfordjournals.aje.a009745  545. Linet MS, Gridley G, Nyrén O, et al. Primary liver cancer, other malignancies, and mortality risks following porphyria: a cohort study in Denmark and Sweden. *Am J Epidemiol*. Jun 1 1999;149(11):1010-5. doi:10.1093/oxfordjournals.aje.a009745  546. Lippi G, Teti L, Dipalo M, Cervellin G. Relationship between red blood cell distribution width and prognostic biomarkers in patients admitted to the emergency department with acute infections. Letter. *European Journal of Internal Medicine*. 2013;24(2):e15-e16. doi:10.1016/j.ejim.2012.09.005  547. Lippi G, Teti L, Scioscioli F, Aloe R, Dipalo M, Cervellin G. Clinical usefulness of procalcitonin and high-sensitive c reactive protein for distinguishing pneumonia from chronic obstructive pulmonary disease in the emergency department. Conference Abstract. *Biochimica Clinica*. 2012;36(6):574.  548. Liu B, Yin Q, Chen YX, Zhao YZ, Li CS. Role of Presepsin (sCD14-ST) and the CURB65 scoring system in predicting severity and outcome of community-acquired pneumonia in an emergency department. Article. *Respiratory Medicine*. 2014;108(8):1204-1213. doi:10.1016/j.rmed.2014.05.005  549. Liu C, Wang K, Li L, et al. Severity of COVID-19 in Cancer patients versus patients without Cancer: A Propensity Score Matching Analysis. Article. *Journal of Cancer*. 2021;12(12):3558-3565. doi:10.7150/JCA.54205  550. Liu C, Wen Y, Wan W, Lei J, Jiang X. Clinical characteristics and antibiotics treatment in suspected bacterial infection patients with COVID-19. Article. *International Immunopharmacology*. 2021;90doi:10.1016/j.intimp.2020.107157  551. Liu D, Chen Q, Zhu H, et al. Association of Respiratory Syncytial Virus Toll-Like Receptor 3-Mediated Immune Response with COPD Exacerbation Frequency. Article. *Inflammation*. 2018;41(2):654-666. doi:10.1007/s10753-017-0720-4  552. Liu D, Li R, Yu R, et al. Alteration of serum markers in COVID-19 and implications on mortality. Letter. *Clinical and Translational Medicine*. 2020;10(3)doi:10.1002/ctm2.119  553. Liu H, Zheng D, Lin Y, et al. Association of sputum microbiome with clinical outcome of initial antibiotic treatment in hospitalized patients with acute exacerbations of COPD. Article. *Pharmacological Research*. 2020;160doi:10.1016/j.phrs.2020.105095  554. Liu HH, Guo JB, Geng Y, Su L. Procalcitonin: present and future. Review. *Irish Journal of Medical Science*. 2015;184(3):597-605. doi:10.1007/s11845-015-1327-0  555. Liu P, Niu R, Chen J, et al. Epidemiological and clinical features in patients with coronavirus disease 2019 outside of wuhan, china: Special focus in asymptomatic patients. Article. *PLoS Neglected Tropical Diseases*. 2021;15(3)doi:10.1371/journal.pntd.0009248  556. Liu SS, Zhang YB. Article. *Chinese Journal of Antibiotics*. 2015;40(6):459-463.  557. Liu X, Deng K, Chen S, et al. 8-hydroxy-2’-deoxyguanosine as a biomarker of oxidative stress in acute exacerbation of chronic obstructive pulmonary disease. Article. *Turkish Journal of Medical Sciences*. 2019;49(1):93-100. doi:10.3906/sag-1807-106  558. Liu X, Lv J, Gan L, et al. Comparative analysis of clinical characteristics, imaging and laboratory findings of different age groups with COVID-19. Article. *Indian Journal of Medical Microbiology*. 2020;38(1):87-93. doi:10.4103/ijmm.IJMM_20_133  559. Liu X, Wen M, Ding H, et al. Effect of hypercapnia on the clinical prognosis and severity of infection in patients with severe community-acquired pneumonia. Article. *Zhonghua Wei Zhong Bing Ji Jiu Yi Xue*. 2020;32(5):564-569. doi:10.3760/cma.j.cn121430-20200122-00099  560. Liu Z, Shi F, Liu J, et al. Effect of the emphysema subtypes of patients with chronic obstructive pulmonary disease on airway inflammation and COTE index. Article. *Experimental and Therapeutic Medicine*. 2018;16(6):4745-4752. doi:10.3892/etm.2018.6799  561. Liu Z, Wang Y, Liu Y, et al. Typing of chronic obstructive pulmonary disease using high-resolution computed tomography and the association with smoking, airway inflammation, and common comorbidities. Article. *Turkish Journal of Medical Sciences*. 2018;48(5):945-951. doi:10.3906/sag-1712-39  562. Livzan MA, Drapkina OM, Nikolaev NA, et al. Algorithms for adult outpatient care of coronavirus disease 2019 (COVID-19) and its assumption. Article. *Cardiovascular Therapy and Prevention (Russian Federation)*. 2021;20(4)doi:10.15829/1728-8800-2021-2916  563. Llor C, Miravitlles M. C-reactive protein point of care testing: the answer to antibiotic prescribing in ambulatory patients with exacerbations of chronic obstructive pulmonary disease? Editorial. *Expert Review of Respiratory Medicine*. 2021;15(1):1-3. doi:10.1080/17476348.2020.1776611  564. Lo Buglio A, Bellanti F, Talia M, Romano AD, Serviddio G, Vendemiale G. Reliability of serum procalcitonin concentration for the diagnosis of sepsis in elderly patient with chronic kidney disease. Article. *Journal of Gerontology and Geriatrics*. 2016;64(2):49-54.  565. Lockley S. Supervised practice and plenty of hard work can greatly enhance pharmacists' clinical skills. Review. *Pharmacy in Practice*. 2006;16(8):222-228.  566. Lommatzsch M, Niewerth A, Klotz J, et al. Platelet and plasma BDNF in lower respiratory tract infections of the adult. Article. *Respiratory Medicine*. 2007;101(7):1493-1499. doi:10.1016/j.rmed.2007.01.003  567. Long B, Targonsky E, Brém E. Just the facts: febrile neutropenia in the emergency department setting. Article. *Canadian Journal of Emergency Medicine*. 2021;23(4):445-449. doi:10.1007/s43678-020-00055-x  568. Lopez-Campos JL, Agustí A. Heterogeneity of chronic obstructive pulmonary disease exacerbations: A two-axes classification proposal. Note. *The Lancet Respiratory Medicine*. 2015;3(9):729-734. doi:10.1016/S2213-2600(15)00242-8  569. Lorente L, Martín MM, González-Rivero AF, et al. Blood concentrations of proapoptotic sFas and antiapoptotic Bcl2 and COVID-19 patient mortality. Article. *Expert Review of Molecular Diagnostics*. 2021;21(8):837-844. doi:10.1080/14737159.2021.1941880  570. Loutsios C, Logan G, Deeming C, Powrie D, Davison A. One blood gas is not enough to assess a patient for ltot-how to kiss goodbye to circa 10 million in England. Conference Abstract. *Thorax*. 2010;65:A171. doi:10.1136/thx.2010.151068.24  571. Lu S, Li T, Xi U, et al. Prognosis of 18 H7N9 avian influenza patients in Shanghai. Article. *PLoS ONE*. 2014;9(4)doi:10.1371/journal.pone.0088728  572. Lu S, Li T, Xi X, et al. Prognosis of 18 H7N9 avian influenza patients in Shanghai. *PLoS One*. 2014;9(4):e88728. doi:10.1371/journal.pone.0088728  573. Lu W, Wu P, He L, et al. Dynamic Antibody Responses in Patients with Different Severity of COVID-19: A Retrospective Study. Article. *Infectious Diseases and Therapy*. 2021;10(3):1379-1390. doi:10.1007/s40121-021-00423-9  574. Luks V, Ahmed S. C REACTIVE PROTEIN-GUIDED ANTIBIOTIC PRESCRIBING VS STANDARD OF CARE IN EXACERBATIONS OF COPD: A SYSTEMATIC REVIEW WITH META-ANALYSIS. Conference Abstract. *Chest*. 2020;158(4):A1788. doi:10.1016/j.chest.2020.08.1559  575. Ma J, Yin J, Qian Y, Wu Y. Clinical characteristics and prognosis in cancer patients with COVID-19: A single center's retrospective study. Letter. *Journal of Infection*. 2020;81(2):318-356. doi:10.1016/j.jinf.2020.04.006  576. Ma K, Bean H, Bremmer D, Moffa M, Watson C, Walsh T. Impact of antibiotic utilization in acute exacerbations of chronic obstructive pulmonary disease in hospitalized patients with normal procalcitonin levels. Conference Abstract. *American Journal of Respiratory and Critical Care Medicine*. 2018;197(MeetingAbstracts)  577. Ma QB, Fu YW, Feng L, et al. Performance of simplified acute physiology score 3 in predicting hospital mortality in emergency intensive care unit. Article. *Chinese Medical Journal*. 2017;130(13):1544-1551. doi:10.4103/0366-6999.208250  578. Macnee W. Systemic inflammatory biomarkers and co-morbidities of chronic obstructive pulmonary disease. Review. *Annals of Medicine*. 2013;45(3):291-300. doi:10.3109/07853890.2012.732703  579. Macrea M, Campbell S, Martin T, Oursler KA. The peripheral neutrophils in subjects with COPD-OSA overlap syndrome and severe comorbidities: A feasible inflammatory biomarker? Article. *Advances in Clinical and Experimental Medicine*. 2018;27(12):1677-1682. doi:10.17219/acem/75904  580. Maddocks LE. What's hot that the other lot got. Note. *Thorax*. 2018;73(10):996. doi:10.1136/thoraxjnl-2018-212517  581. Magnussen H, Watz H, Kirsten A, et al. Stepwise withdrawal of inhaled corticosteroids in COPD patients receiving dual bronchodilation: WISDOM study design and rationale. Article. *Respiratory Medicine*. 2014;108(4):593-599. doi:10.1016/j.rmed.2014.01.002  582. Mahendran AJ, Gupta N, Agrawal S, Ish P. Colistin-induced acquired bartter-like syndrome: A rare cause of difficult weaning. Letter. *Indian Journal of Critical Care Medicine*. 2020;24(8):739-740. doi:10.5005/jp-journals-10071-23551  583. Mahmood T, Singh NK, Singh AK. Analysis of clinical sign and symptoms and biochemical parameters of post-COVID patients. Letter. *Lung India*. 2022;39(2):214-215. doi:10.4103/lungindia.lungindia_165_21  584. Maini R, Ranjha S, Tandan N, et al. Pulmonary Blastomycosis: A case series and review of unique radiological findings. Article. *Medical Mycology Case Reports*. 2020;28:49-54. doi:10.1016/j.mmcr.2020.03.006  585. Mair FS, Hiscock J, Beaton SC. Understanding factors that inhibit or promote the utilization of telecare in chronic lung disease. Article. *Chronic Illness*. 2008;4(2):110-117. doi:10.1177/1742395308092482  586. Maisel A, Neath SX, Landsberg J, et al. Use of procalcitonin for the diagnosis of pneumonia in patients presenting with a chief complaint of dyspnoea: Results from the BACH (Biomarkers in Acute Heart Failure) trial. Article. *European Journal of Heart Failure*. 2012;14(3):278-286. doi:10.1093/eurjhf/hfr177  587. Makhlouf HA, Sadek SH, Nafady AAH. Platelet function in diabetic and nondiabetic patients with chronic obstructive pulmonary disease: a case control study. Article. *Clinical Respiratory Journal*. 2018;12(1):48-56. doi:10.1111/crj.12477  588. Mammadov F, Olgun Yildizeli S, Kocakaya D, et al. The role of procalcitonin as a biomarker for acute pulmonary exacerbation in subjects with cystic fibrosis and non-cystic fibrosis bronchiectasis. Article. *Marmara Medical Journal*. 2022;35(2):164-171. doi:10.5472/marumj.1114952  589. Manalan K, Rashid T, Singanayagam A. Antibiotic treatment in exacerbations of chronic obstructive pulmonary disease: Recent trial results. Article. *Clinical Investigation*. 2015;5(2):189-204. doi:10.4155/cli.14.113  590. Mancini A, M. Corbo G, Inchingolo R, et al. Relationships between testosterone and severity indices in male patients with chronic obstructive pulmonary diseases. Conference Abstract. *International Journal of Andrology*. 2010;33:51-52. doi:10.1111/j.1365-2605.2010.01106.x  591. Mansour G, Salih M, Kukreja R, Vivekanandan R, Morrow L. COPD exacerbation, does procalcitonin help? A metaanalysis. Conference Abstract. *Chest*. 2015;148(4)doi:10.1378/chest.2281284  592. Mao Y, Qian Y, Sun X, Li N, Huang H. Eosinopenia Predicting Long-term Mortality in Hospitalized Acute Exacerbation of COPD Patients with Community-acquired Pneumonia— A Retrospective Analysis. Article. *International Journal of COPD*. 2021;16:3551-3559. doi:10.2147/COPD.S347948  593. Mao Y, Qian Y, Sun X, Li N, Huang H. Eosinopenia Predicting Long-term Mortality in Hospitalized Acute Exacerbation of COPD Patients with Community-acquired Pneumonia-A Retrospective Analysis. *Int J Chron Obstruct Pulmon Dis*. 2021;16:3551-3559. doi:10.2147/copd.S347948  594. Maouelainin N, Hoag JB, Hillman J, Hendry MA. Characterization of serum procalcitonin at baseline and during pulmonary exacerbations in patients with cystic fibrosis. Conference Abstract. *Pediatric Pulmonology*. 2011;46:301. doi:10.1002/ppul.21583  595. Maranatha D, Mawardi, Hamzah. Severe pneumonia: Etiology and outcome in a tertiary hospital in Indonesia. Article. *Indian Journal of Forensic Medicine and Toxicology*. 2021;15(2):2192-2200. doi:10.37506/ijfmt.v15i2.14698  596. Marsden E, Cubbin I, McAlavey A. An investigation into how poor compliance traditionally associated with corticosteroid therapy in asthma and chronic obstructive pulmonary disease can be improved to enhance long-term management and patient care. Conference Abstract. *International Journal of Pharmacy Practice*. 2009;17(S2):B55-B56.  597. Martinez FJ, Curtis JL. Procalcitonin-guided antibiotic therapy in COPD exacerbations: Closer but not quite there. Editorial. *Chest*. 2007;131(1):1-2. doi:10.1378/chest.06-2567  598. Maselli DJ, Restrepo MI. Pneumonia or exacerbation of copd? Article. *ERS Monograph*. 2015;2015(9781849840644):185-196. doi:10.1183/2312508X.10018414  599. Masiá M, Padilla S, García JA, et al. Impact of the Addition of Baricitinib to Standard of Care Including Tocilizumab and Corticosteroids on Mortality and Safety in Severe COVID-19. Article. *Frontiers in medicine*. 2021;8doi:10.3389/fmed.2021.749657  600. Masters B, Han MK, Martinez FJ, et al. Blood biomarker algorithms as an aide to describe exacerbations activity in symptomatic smokers and early staged COPD patients. Conference Abstract. *American Journal of Respiratory and Critical Care Medicine*. 2018;197(MeetingAbstracts)  601. Mathioudakis AG, Chatzimavridou-Grigoriadou V, Corlateanu A, Vestbo J. Clinical effectiveness of procalcitonin based protocols to guide the administration of antibiotics in patients presenting with COPD exacerbations: Systematic review and metaanalysis. Conference Abstract. *Thorax*. 2016;71:A110-A111. doi:10.1136/thoraxjnl-2016-209333.194  602. Mathioudakis AG, Chatzimavridou-Grigoriadou V, Corlateanu A, Vestbo J. Procalcitonin to guide antibiotic administration in COPD exacerbations: A meta-analysis. Review. *European Respiratory Review*. 2017;26(143)doi:10.1183/16000617.0073-2016  603. Mathioudakis AG, Moberg M, Janner J, Alonso-Coello P, Vestbo J. Outcomes reported on the management of COPD exacerbations: A systematic survey of randomised controlled trials. Article. *ERJ Open Research*. 2019;5(2)doi:10.1183/23120541.00072-2019  604. Mathioudakis AG, Vanfleteren LEGW, Lahousse L, et al. Current developments and future directions in COPD. Article. *European Respiratory Review*. 2020;29(158):1-12. doi:10.1183/16000617.0289-2020  605. Mathioudakis AG, Vestbo J. Was the implementation strategy of the ProACT trial adequately proactive? Note. *Breathe*. 2019;15(1):77-80. doi:10.1183/20734735.0338-2018  606. McBryan D, Willey V, Zubek V, et al. Assessment in a real-world setting of the effect of inhaled steroid-based triple therapy versus the combination of tiotropium and olodaterol on reducing chronic obstructive pulmonary disease exacerbations: Airwise study design. Conference Abstract. *Journal of Managed Care and Specialty Pharmacy*. 2017;23:S64.  607. McBryan D, Willey V, Zubek V, et al. Assessment in a real-world setting of the effect of inhaled steroid-based triple therapy versus the combination of tiotropium and olodaterol on reducing chronic obstructive pulmonary disease exacerbations: airwise study design. Conference Abstract. *Journal of managed care and specialty pharmacy*. 2017;23(10‐A Suppl):S64.  608. McGinnis K, Rolek K, Van Schooneveld TC. Real-world experience with procalcitonin use for lower respiratory tract infections. Conference Abstract. *Open Forum Infectious Diseases*. 2016;3doi:10.1093/ofid/ofw172.97  609. Medetalibeyoğlu A, Şenkal N, Çapar G, Köse M, Tükek T. Characteristics of the initial patients hospitalized for COVID-19: A single-center report. Letter. *Turkish Journal of Medical Sciences*. 2020;50(5):1436-1439. doi:10.3906/sag-2004-98  610. Megri M, Morris M, Morehead S. FIBROTIC PROTEASE 3 ANCA-ASSOCIATED VASCULITIS. Conference Abstract. *Chest*. 2020;158(4):A1095. doi:10.1016/j.chest.2020.08.1005  611. Mei C, Shah R, Fisher MJ, Marcos LA, Mansour M. A peculiar case of sepsis: The role of procalcitonin in diagnosing human granulocytic anaplasmosis. Conference Abstract. *American Journal of Respiratory and Critical Care Medicine*. 2018;197(MeetingAbstracts)  612. Meier MA, Ottiger M, Vögeli A, et al. Activation of the Serotonin Pathway is Associated with Poor Outcome in COPD Exacerbation: Results of a Long-Term Cohort Study. Article. *Lung*. 2017;195(3):303-311. doi:10.1007/s00408-017-0004-7  613. Meili M, Kutz A, Briel M, et al. Infection biomarkers in primary care patients with acute respiratory tract infections-comparison of Procalcitonin and C-reactive protein. Journal: Article. *BMC pulmonary medicine*. 2016;16(1) (no pagination)doi:10.1186/s12890-016-0206-4  614. Menegolo M, Colacchio EC, Piazza M, Ello MA, Grego F. Current management of inflammatory abdominal aortic aneurysms: A systematic review. Review. *Italian Journal of Vascular and Endovascular Surgery*. 2018;25(1):50-58. doi:10.23736/S1824-4777.17.01319-5  615. Menon N, Evans R, Graham JH, et al. Adherence and outcomes of implementing the provencare® model in patients with COPD admitted for exacerbations. Conference Abstract. *American Journal of Respiratory and Critical Care Medicine*. 2015;191  616. Menzel A, Samouda H, Dohet F, Loap S, Ellulu MS, Bohn T. Common and Novel Markers for Measuring Inflammation and Oxidative Stress Ex Vivo in Research and Clinical Practice-Which to Use Regarding Disease Outcomes? *Antioxidants (Basel, Switzerland)*. Mar 9 2021;10(3)doi:10.3390/antiox10030414  617. Meteran H, Sivapalan P, Stæhr Jensen JU. Treatment response biomarkers in asthma and copd. Article. *Diagnostics*. 2021;11(9)doi:10.3390/DIAGNOSTICS11091668  618. Mikelsons C, Buxton M. First national survey of the respiratory physiotherapy workforce. Conference Abstract. *Thorax*. 2010;65:A171-A172. doi:10.1136/thx.2010.151068.26  619. Miravitlles M. Is it possible to reduce the use of antibiotics in severe exacerbations of chronic obstructive pulmonary disease? Editorial. *Respiration*. 2008;76(3):249-250. doi:10.1159/000147952  620. Miravitlles M, Sotgiu G, Dimopoulos G, et al. The best on infections: Update from the 2010 ERS Congress. Review. *European Respiratory Journal*. 2011;38(2):450-455. doi:10.1183/09031936.00040011  621. Mishra AK, George AA, Sahu KK, Lal A, Abraham G. Review of clinical profile, risk factors, and outcomin patients with tuberculosis and covid-19. Review. *Acta Biomedica*. 2021;92(1)doi:10.23750/abm.v92i1.10738  622. Mishra M, Zahra A, Chauhan LV, et al. A Short Series of Case Reports of COVID-19 in Immunocompromised Patients. Article. *Viruses*. 2022;14(5)doi:10.3390/v14050934  623. Mitsuma SF, Mansour MK, Dekker JP, et al. Promising new assays and technologies for the diagnosis and management of infectious diseases. Review. *Clinical Infectious Diseases*. 2013;56(7):996-1002. doi:10.1093/cid/cis1014  624. Mitsumura T, Okamoto T, Shirai T, et al. Predictors associated with clinical improvement of SARS-CoV-2 pneumonia. Article. *Journal of Infection and Chemotherapy*. 2021;27(6):857-863. doi:10.1016/j.jiac.2021.02.012  625. Moeckli B, Limani P, Clavien PA, Vonlanthen R. Parastomal gallbladder herniation: A case report and review of the literature. Article. *International Journal of Surgery Case Reports*. 2020;73:338-341. doi:10.1016/j.ijscr.2020.07.002  626. Mohamadi M, Fattahi N, Goodarzi A, et al. A comprehensive review on covid-19 infection and comorbidities of various organs. Article. *Acta Medica Iranica*. 2021;59(1):4-14.  627. Momeni B, Nazer S, Masoompour SM, Geramizadeh B, Sajadi SV. The effect of atorvastatin on inflammatory markers in sulfur mustard gas induced bronchitis: a randomized double-blinded, placebo-control clinical trial. Journal Article; Randomized Controlled Trial. *BMC pulmonary medicine*. 2021;21(1):112. doi:10.1186/s12890-021-01481-y  628. Morales JE, Emerick BP, Griffin PL, Grill J. Alveolar hemorrhage and respiratory failure secondary to apixaban. Conference Abstract. *American Journal of Respiratory and Critical Care Medicine*. 2018;197(MeetingAbstracts)  629. Mori G, Alfano G, Fontana F, Magistroni R. Hybrid dialysis: A promising strategy to reduce hospital access during the SARS-CoV-2 pandemic. Article. *BMJ Case Reports*. 2020;13(10)doi:10.1136/bcr-2020-236411  630. Moriates C, Maisel A. The Utility of Biomarkers in Sorting Out the Complex Patient. Review. *American Journal of Medicine*. 2010;123(5):393-399. doi:10.1016/j.amjmed.2009.07.034  631. Mortensen EM. Potential causes of increased long-term mortality after pneumonia. Editorial. *European Respiratory Journal*. 2011;37(6):1306-1307. doi:10.1183/09031936.00194110  632. Moschovis PP, Lu M, Hayden D, et al. Effect modification by age of the association between obstructive lung diseases, smoking, and COVID-19 severity. Article. *BMJ Open Respiratory Research*. 2021;8(1)doi:10.1136/bmjresp-2021-001038  633. Motahari A, Barr R, Han MK, et al. Contribution of air-trapped Vs. Non-air-trapped emphysematous lung regions to a link between CT-based metrics and spirometry. SPIROMICS. Conference Abstract. *American Journal of Respiratory and Critical Care Medicine*. 2018;197(MeetingAbstracts)  634. Mou S, Zhang W, Deng Y, Tang Z, Jiang D. Comparison of CRP, Procalcitonin, Neutrophil Counts, Eosinophil Counts, sTREM-1, and OPN between Pneumonic and Nonpneumonic Exacerbations in COPD Patients. Article. *Canadian Respiratory Journal*. 2022;2022doi:10.1155/2022/7609083  635. Mueller C, Christ M, Cowie M, et al. European Society of Cardiology-Acute Cardiovascular Care Association Position paper on acute heart failure: A call for interdisciplinary care. Review. *European Heart Journal: Acute Cardiovascular Care*. 2017;6(1):81-86. doi:10.1177/2048872615593279  636. Mueller C, Christ-Crain M, Müller B. What cardiologists do need to know about procalcitonin. Review. *Clinical Laboratory*. 2005;51(1-2):1-4.  637. Muhktar U, Athar M, Boorman D, et al. Procalcitonin as a test to differentiate between central and infectious fever in SAH patients. Conference Abstract. *Critical Care Medicine*. 2016;44(12):258. doi:10.1097/01.ccm.0000509403.29382.68  638. Müller B, Morgenthaler N, Stolz D, et al. Circulating levels of copeptin, a novel biomarker, in lower respiratory tract infections. Article. *European Journal of Clinical Investigation*. 2007;37(2):145-152. doi:10.1111/j.1365-2362.2007.01762.x  639. Müller B, Prat C. Markers of acute inflammation in assessing and managing lower respiratory tract infections: Focus on procalcitonin. Review. *Clinical Microbiology and Infection*. 2006;12(SUPPL. 9):8-16. doi:10.1111/j.1469-0691.2006.01654.x  640. Müller B, Schuetz P, Trampuz A. Circulating biomarkers as surrogates for bloodstream infections. Article. *International Journal of Antimicrobial Agents*. 2007;30(SUPPL. 1):16-23. doi:10.1016/j.ijantimicag.2007.06.032  641. Murat B, Murat S, Mert KU, Bilgin M, Cavusoglu Y. Clinical characteristics and in-hospital outcomes of COVID-19 patients with history of heart failure: a propensity score-matched study. Article. *Acta Cardiologica*. 2022;77(1):37-44. doi:10.1080/00015385.2021.1945765  642. Murray DD, Itenov TS, Sivapalan P, et al. Biomarkers of acute lung injury the individualized approach: For phenotyping, risk stratification and treatment surveillance. Review. *Journal of Clinical Medicine*. 2019;8(8)doi:10.3390/jcm8081163  643. Musheyev B, Borg L, Janowicz R, et al. Functional status of mechanically ventilated COVID-19 survivors at ICU and hospital discharge. Article. *Journal of Intensive Care*. 2021;9(1)doi:10.1186/s40560-021-00542-y  644. Naidoo K, De Vasconcellos K, Skinner DL. Procalcitonin kinetics in the first 48 hours of ICU admission is associated with higher mortality in critically ill patients with community-acquired pneumonia in a setting of high HIV prevalence. Article. *Southern African Journal of Anaesthesia and Analgesia*. 2018;24(5):128-134. doi:10.1080/22201181.2018.1514787  645. Nair P. Case of a messy rash. Conference Abstract. *Journal of Hospital Medicine*. 2018;13(4)  646. Nancy Giovanna Uribe Heredia NG, Arroyo Espligueroi R, Piccone Saponara LG, et al. Evaluation of the cardiac efficiency to the exercise in patients with chronic bronchopathy and ischemic cardiomyopathy. Conference Abstract. *European Journal of Heart Failure*. 2019;21:283-284. doi:10.1002/ejhf.1488  647. Nangia V, Gandhi K. Use of procalcitonin to guide the antibiotic therapy in patients with an acute exacerbation of COPD in a resource-limited setting: A case-control study. Conference Abstract. *Clinical Microbiology and Infection*. 2012;18:64. doi:10.1111/j.1469-0691.2012.03801.x  648. Narendra DK, Hanania NA. Procalcitonin in chronic obstructive pulmonary disease exacerbations: Is it ready for primetime use? Editorial. *Annals of the American Thoracic Society*. 2017;14(12):1757-1758. doi:10.1513/AnnalsATS.201708-673ED  649. Natanov R, Wiesner O, Haverich A, Kuhn C. Mechanical circulatory support in coronavirus disease-2019-positive patients with severe respiratory failure. Article. *Interactive Cardiovascular and Thoracic Surgery*. 2021;33(3):489-493. doi:10.1093/icvts/ivab087  650. Nct. Procalcitonin Guided Antibiotic Therapy and Hospitalisation in Patients With Lower Respiratory Tract Infections: The "ProHOSP" Study. *<https://clinicaltrialsgov/show/NCT00350987>*. 2006;  651. Nct. Procalcitonin as a Marker of Bacterial Pneumonia. *<https://clinicaltrialsgov/show/NCT00415753>*. 2006;  652. Nct. Comparison of a Serum PRO-CT Guided Treatment and the Recommended Antibiotic Treatment for COPD. *<https://clinicaltrialsgov/show/NCT01125098>*. 2010;  653. Nct. Ceftidoren Versus Levofloxacin in the Treatment of Patients With Acute Exacerbations of Chronic Bronchitis (AECB). *<https://clinicaltrialsgov/show/NCT01467297>*. 2011;  654. Nct. Procalcitonin To Reduce Antibiotics in Chronic Obstructive Lung Disease (ProToCOLD). *<https://clinicaltrialsgov/show/NCT01950936>*. 2012;  655. Nct. Clinical Trial Assessing the Value of an Antibiotic Protocol Guided by Serum Procalcitonin in Acute Exacerbations of Chronic Obstructive Pulmonary Disease in Intensive Care. *<https://clinicaltrialsgov/show/NCT02521636>*. 2015;  656. Nct. Cholecalciferol Supplementation in Critically Ill Patients With Severe Vitamin D Deficiency. *<https://clinicaltrialsgov/show/NCT02868827>*. 2016;  657. Nct. Procalcitonin-guided Antibiotic Therapy During Severe Acute Exacerbation of COPD. *<https://clinicaltrialsgov/show/NCT03440060>*. 2018;  658. Nct. A Trial of Procalcitonin in Patients With Acute Exacerbation of Chronic Obstructive Pulmonary Disease. *<https://clinicaltrialsgov/show/NCT04682899>*. 2020;  659. Nct. Short Term, High Dose Vitamin D Supplementation for COVID-19. *<https://clinicaltrialsgov/show/NCT04459247>*. 2020;  660. Nct. Investigation the Effect of Montelukast in COVID-19. *<https://clinicaltrialsgov/show/NCT04718285>*. 2021;  661. Nct. Efficacy of the Infusion of Donor Plasma in COVID-19 Infection. *<https://clinicaltrialsgov/show/NCT05247307>*. 2022;  662. Nct. Use of MULTIplex PCR, Procalcitonin, and Sputum Appearance to Reduce Duration of Antibiotic Therapy During Severe COPD EXAcerbation: a Controlled, Randomized, Open-label, Parallel-Group, Multicenter Trial. *<https://clinicaltrialsgov/show/NCT05280132>*. 2022;  663. Nemoto D, Hitomi S, Moriyama Y, Iwamoto K, Saito K. Cellulitis complicated with bacteremia due to Sphingobacterium species: A report of two cases and a literature review. Article. *Internal Medicine*. 2019;58(17):2573-2576. doi:10.2169/internalmedicine.2178-18  664. Neunhauserer D, Steidle-Kloc E, Niederseer D, et al. The effect of supplemental oxygen during exercise training on systemic inflammation and cardiovascular function in patients with COPD: a double-blind randomized controlled trial. Journal: Conference Abstract. *Wiener klinische Wochenschrift*. 2017;129(19‐20):759‐. doi:10.1007/s00508-017-1273-0  665. Neunhäuserer D, Steidle-Kloc E, Niederseer D, et al. The effect of supplemental oxygen during exercise training on systemic inflammation and cardiovascular function in patients with COPD: A double-blind randomized controlled trial. Conference Abstract. *Wiener Klinische Wochenschrift*. 2017;129(19-20):759. doi:10.1007/s00508-017-1273-0  666. Neuville M, Vinclair C, Cally R, Bouadma L. Place of biomarkers in the management of pulmonary infections. Review. *Revue des Maladies Respiratoires*. 2019;36(3):405-414. doi:10.1016/j.rmr.2018.09.003  667. Ng Cheong Chung KJ, Kunadian V. Frailty assessment in the COVID-19 pandemic. Letter. *Journal of Investigative Medicine*. 2020;68(7):1300-1301. doi:10.1136/jim-2020-001540  668. Nguyen HQ, Moy ML, Fan VS, et al. Applying the pragmatic-explanatory continuum indicator summary to the implementation of a physical activity coaching trial in chronic obstructive pulmonary disease. Journal: Article. *Nursing outlook*. 2018;66(5):455‐463. doi:10.1016/j.outlook.2018.05.005  669. Nguyen LJ, Varker A, Slaughter P, Boyle D, Nekahi N. Procalcitonin-Guided Antibiotic Prescribing for Acute Exacerbations of Chronic Obstructive Pulmonary Disease in the Emergency Department. *Federal practitioner : for the health care professionals of the VA, DoD, and PHS*. Jun 2021;38(6):264-269. doi:10.12788/fp.0141  670. Ni W, Bao J, Yang D, et al. Potential of serum procalcitonin in predicting bacterial exacerbation and guiding antibiotic administration in severe COPD exacerbations: a systematic review and meta-analysis. Review. *Infectious Diseases*. 2019;51(9):639-650. doi:10.1080/23744235.2019.1644456  671. Ni Y, Ding L, Yu Y, Dai R, Chen H, Shi G. Oscillatory positive expiratory pressure treatment in lower respiratory tract infection. Article. *Experimental and Therapeutic Medicine*. 2018;16(4):3241-3248. doi:10.3892/etm.2018.6552  672. Nicholas BL. Search for biomarkers in chronic obstructive pulmonary disease: Current status. Review. *Current Opinion in Pulmonary Medicine*. 2013;19(2):103-108. doi:10.1097/MCP.0b013e32835ceee5  673. Niederman MS. Making sense of scoring systems in community acquired pneumonia. Article. *Respirology*. 2009;14(3):327-335. doi:10.1111/j.1440-1843.2009.01494.x  674. Nielsen SK, Lange P. Not all exacerbations of chronic obstructive pulmonary disease should be treated with antibiotics. Review. *Ugeskrift for laeger*. 2017;179(15)  675. Niewoehner DE. Procalcitonin level-guided treatment reduced antibiotic use in exacerbations of COPD. *ACP journal club*. May-Jun 2007;146(3):57.  676. Niu Y, Xing Y, Li J, et al. Effect of Community-Acquired Pneumonia on Acute Exacerbation of Chronic Obstructive Pulmonary Disease. Article. *COPD: Journal of Chronic Obstructive Pulmonary Disease*. 2021;18(4):417-424. doi:10.1080/15412555.2021.1950664  677. Nl. Procalcitonin-guided treatment regarding antibiotic use for acute COPD exacerbations: a prospective randomised controlled trial. *<https://trialsearchwhoint/Trial2aspx?TrialID=NL9122>*. 2020;  678. Noorjahan M, Sreedevi, SaiBaba KSS, et al. Biochemical and clinical risk factors for mortality in COVID-19 patients in ICU: A single-center retrospective study. Conference Abstract. *Indian Journal of Clinical Biochemistry*. 2021;36(SUPPL 1):S150-S151. doi:10.1007/s12291-021-01019-3  679. Nseir S, Cavestri B, Di Pompeo C, et al. Factors predicting bacterial involvement in severe acute exacerbations of chronic obstructive pulmonary disease. Article. *Respiration*. 2008;76(3):253-260. doi:10.1159/000139611  680. Nuzhdin MD, Prihodko VP, Fokin AA, Malinovsky YV. Early-and long-term results after surgical resection of primary cardiac tumors. Conference Abstract. *Interactive Cardiovascular and Thoracic Surgery*. 2011;12:S129. doi:10.1510/icvts.2011.0000S1  681. Öcal N, Yavuz Veizi BG, Cüce F, et al. How did covid-19 pandemic affect the older patients? Comparison of clinical features in older versus younger patients. Article. *Turk Geriatri Dergisi*. 2020;23(4):434-445. doi:10.31086/TJGERI.2020.181  682. Ocal N, Yavuz Veizi BG, Cuce F, et al. How did covid-19 pandemic affect the older patients? Comparison of clinical features in older versus younger patients. Journal: Article. *Turk geriatri dergisi*. 2020;23(4):434‐445. doi:10.31086/TJGERI.2020.181  683. Odeh B, Kayyali R, Nabhani S, et al. Impact of telehealth on patients' outcomes-A patients' perceptions evaluation study. Journal: Conference Abstract. *International journal of pharmacy practice*. 2013;21:104‐105. doi:10.1111/ijpp.12064  684. Odeh B, Kayyali R, Nabhani-Gebara S, Philip N. Implementing a telehealth service: nurses' perceptions and experiences. Article. *British journal of nursing (Mark Allen Publishing)*. 2014;23(21):1133-1137. doi:10.12968/bjon.2014.23.21.1133  685. Oi I, Ito I, Hirabayashi M, et al. Pneumonia Caused by Severe Acute Respiratory Syndrome Coronavirus 2 and Influenza Virus: A Multicenter Comparative Study. Article. *Open Forum Infectious Diseases*. 2021;8(7)doi:10.1093/ofid/ofab282  686. O'Kelly N, Smith J. Palliative care for patients with end-stage COPD. Letter. *Primary Care Respiratory Journal*. 2007;16(1):57-58. doi:10.3132/pcrj.2007.00003  687. Omernik A. Serum procalcitonin level in lower respiratory track infections. Review. *International Review of Allergology and Clinical Immunology in Family Medicine*. 2012;18(2):102-109.  688. Onoue S, Hashimoto N, Yamada S. Dry powder inhalation systems for pulmonary delivery of therapeutic peptides and proteins. Review. *Expert Opinion on Therapeutic Patents*. 2008;18(4):429-442. doi:10.1517/13543776.18.4.429  689. Oppen K, Ueland T, Siljan WW, et al. Hepcidin and ferritin predict microbial etiology in community-acquired pneumonia. Article. *Open Forum Infectious Diseases*. 2021;8(4)doi:10.1093/ofid/ofab082  690. Ortiz Velez I, Fonseca Ferrer V, Roman-Velez N, et al. "SHATTERED LUNGS": A RARE CASE OF A PULMONARY CAVITARY LESION BY MYCOBACTERIUM KANSASII. Conference Abstract. *Chest*. 2021;160(4):A426-A427. doi:10.1016/j.chest.2021.07.422  691. Ostendorf U, Ewig S, Torres A. Nosocomial pneumonia. Review. *Current Opinion in Infectious Diseases*. 2006;19(4):327-338. doi:10.1097/01.qco.0000235158.40184.28  692. Otero R, Garcia AJ. Clinical review: New technologies - Venturing out of the intensive care unit. Review. *Critical Care*. 2005;9(3):296-302. doi:10.1186/cc2982  693. Ott SR, Lepper PM, Hauptmeier B, et al. The impact of viruses in lower respiratory tract infections of the adult. Review. *Pneumologie*. 2009;63(12):709-717. doi:10.1055/s-0029-1215232  694. Öz M, Gürün Kaya A, Yakut ÖV, et al. Successful treatment of covid-19 infection in a patient with tracheostomy. Article. *Tuberkuloz ve Toraks*. 2020;68(4):444-448. doi:10.5578/tt.70357  695. Ozkan S, Kahveci U, Hur I, Halici A. Prognostic importance of serum presepsin level in pneumonia focal sepsis and its relationship with other biomarkers and clinical severity scores. Article. *Saudi Medical Journal*. 2021;42(9):994-1001. doi:10.15537/SMJ.2021.42.9.20210163  696. Ozkaya S, Omercikoglu S, Altunbas E, Akoglu H, Denizbasi A. Association of acute phase reactants with prognostic scores in community acquired pneumonia. Article. *Journal of the Pakistan Medical Association*. 2021;71(2 B):614-618. doi:10.47391/JPMA.630  697. Palma G, Pasqua T, Silvestri G, et al. PI3Kδ Inhibition as a Potential Therapeutic Target in COVID-19. Review. *Frontiers in Immunology*. 2020;11doi:10.3389/fimmu.2020.02094  698. Paluero JE, Tokhi L, Roglic M, Labor M. Eosinopenia in COPD patients with severe acute respiratory virus 2 (Sars-CoV-2)infection as a potential fatal outcome predictor. Conference Abstract. *European Respiratory Journal*. 2021;58(SUPPL 65)doi:10.1183/13993003.congress-2021.PA961  699. Pancani F, Pavani R, Quacquarelli A, Feri M. Successful use of CytoSorb in a Covid-19 patient with secondary septic shock due to a sacral decubitus infection. Article. *International Journal of Artificial Organs*. 2021;44(12):1034-1038. doi:10.1177/03913988211016473  700. Pantazopoulos I, Magounaki K, Kotsiou O, et al. Incorporating Biomarkers in COPD Management: The Research Keeps Going. Review. *Journal of Personalized Medicine*. 2022;12(3)doi:10.3390/jpm12030379  701. Papadopoulos A, Bartziokas K, Kostikas K. The role of procalcitonin in the management of patients with sepsis and respiratory tract infections: From bench to bedside. Review. *Pneumon*. 2010;23(4):369-375.  702. Papakonstantinou E, Grize L, Hirsch H, Tamm M, Stolz D. Hyaluronic acid in COPD exacerbations of different etiology. Journal: Conference Abstract. *European respiratory journal*. 2020;56doi:10.1183/13993003.congress-2020.967  703. Papakonstantinou E, Roth M, Tamm M, Stolz D. Serum glycosaminoglycans are differentially associated with collagen synthesis and degradation in COPD. Conference Abstract. *Respiration*. 2019;97(6):600. doi:10.1159/000499887  704. Papakonstantinou E, Stolz D. Biomarkers and rapid diagnostics in antibiotic stewardship. Article. *Minerva Pneumologica*. 2016;55(4):94-105.  705. Para O, Pieralli F, Mancini A, et al. Usefulness of procalcitonin and lactate kinetics as prognostic biomarkers in severe sepsis and septic shock in an Internal High Dependency Unit. Conference Abstract. *Italian Journal of Medicine*. 2014;8:95. doi:10.4081/itjm.2014.s2  706. Parenica J, Jarkovsky J, Malaska J, et al. Infectious complications and immune/inflammatory response in cardiogenic shock patients: A prospective observational study. Article. *Shock*. 2017;47(2):165-174. doi:10.1097/SHK.0000000000000756  707. Park MH, Kim MJ, Kim AJ, Lee MJ, Kim JS. Helmet-based noninvasive ventilation for acute exacerbation of chronic obstructive pulmonary disease: A case report. Article. *World Journal of Clinical Cases*. 2020;8(10):1939-1943. doi:10.12998/wjcc.v8.i10.1939  708. Pascual JM, Carrión F, Sánchez C, Sánchez B, González C. Nutritional changes in patients with advanced chronic obstructive pulmonary disease. Article. *Medicina clínica*. 1996;107(13):486-489.  709. Patel N, Belcher J, Thorpe G, Forsyth NR, Spiteri MA. Measurement of C-reactive protein, procalcitonin and neutrophil elastase in saliva of COPD patients and healthy controls: Correlation to self-reported wellbeing parameters. Article. *Respiratory Research*. 2015;16(1)doi:10.1186/s12931-015-0219-1  710. Paternoster G, Nagy A, Vitiello M, et al. Igm-enriched-immunoglobulins associated with EMiC2 filter in the treatment of early septic shock after cardiac surgery. Conference Abstract. *Journal of Cardiothoracic and Vascular Anesthesia*. 2019;33:S160-S161. doi:10.1053/j.jvca.2019.07.040  711. Patil S. Role of noninvasive positive pressure ventilation (NIPPV). Conference Abstract. *Indian Journal of Critical Care Medicine*. 2020;24(SUPPL 2):S39-S40. doi:10.5005/jp-journals-10071-23353.121  712. Pawar D, Arif D, Raghunath A, Rehman S. Urothelial carcinoma with mandibular metastasis and synchronous prostate cancer. Article. *BMJ Case Reports*. 2022;15(3)doi:10.1136/bcr-2021-247419  713. Pázmány P, Soós A, Hegyi P, et al. Inflammatory Biomarkers Are Inaccurate Indicators of Bacterial Infection on Admission in Patients With Acute Exacerbation of Chronic Obstructive Pulmonary Disease—A Systematic Review and Diagnostic Accuracy Network Meta-Analysis. Review. *Frontiers in medicine*. 2021;8doi:10.3389/fmed.2021.639794  714. Pázmány P, Soós A, Hegyi P, et al. Inflammatory Biomarkers Are Inaccurate Indicators of Bacterial Infection on Admission in Patients With Acute Exacerbation of Chronic Obstructive Pulmonary Disease-A Systematic Review and Diagnostic Accuracy Network Meta-Analysis. *Frontiers in medicine*. 2021;8:639794. doi:10.3389/fmed.2021.639794  715. Pearce LJ, Cecil E, Phillips C, Pulimood TB, Laroche CM. Report of a respiratory health check in a selfselected group of male prisoners in suffolk. Conference Abstract. *Thorax*. 2011;66:A119. doi:10.1136/thoraxjnl-2011-201054c.129  716. Penagos MAE, Tovar KER, Hernandez EED, Martinez ELV. Biochemical differences in patients with COPD due to tobacco and COPD by Biomass hospitalized in the intensive care unit. Conference Abstract. *European Respiratory Journal*. 2019;54doi:10.1183/13993003.congress-2019.OA292  717. Peng X, Chen Y, Deng L, et al. Clinical features of critically ill patients infected with SARS-CoV-2 outside Wuhan with and without diabetes. Article. *International Journal of Diabetes in Developing Countries*. 2020;40(4):482-490. doi:10.1007/s13410-020-00888-3  718. Peng Z, Zhan Q, Xie X, et al. Association between admission plasma 2-oxoglutarate levels and short-term outcomes in patients with acute heart failure: A prospective cohort study. Article. *Molecular Medicine*. 2019;25(1)doi:10.1186/s10020-019-0078-1  719. Pepe M, Maroun-Eid C, Romero R, et al. Clinical presentation, therapeutic approach, and outcome of young patients admitted for COVID-19, with respect to the elderly counterpart. Article. *Clinical and Experimental Medicine*. 2021;21(2):249-268. doi:10.1007/s10238-021-00684-1  720. Pereira JM, Teixeira-Pinto A, Basílio C, Sousa-Dias C, Mergulhão P, Paiva JA. Can we predict pneumococcal bacteremia in patients with severe community-acquired pneumonia? *J Crit Care*. Dec 2013;28(6):970-4. doi:10.1016/j.jcrc.2013.04.016  721. Pérez-Bogerd S, Fremault A, Decramer M, Janssens W. Criteria for antibiotic therapy in acute exacerbations of COPD. Letter. *American Journal of Respiratory and Critical Care Medicine*. 2010;182(9):1207. doi:10.1164/ajrccm.182.9.1207  722. Perincek G, Avci S, Yilmam I. Comparison of complete blood counts of stable COPD patients at two different altitude in Turkey. Journal: Conference Abstract. *Turkish thoracic journal*. 2019;20:S225‐. doi:10.5152/TurkThoracJ.2019.225  723. Perinçek G, Avci S, Yilmam I. Comparison of complete blood counts of stable COPD patients at two different altitude in Turkey. Conference Abstract. *Turkish Thoracic Journal*. 2019;20:S225. doi:10.5152/TurkThoracJ.2019.225  724. Perincek G, Karakayall M, Avcl S. Relationship between ECG Findings and Serum Biomarkers in COVID-19 Patients. Article. *Journal of Cardiovascular Emergencies*. 2021;7(3):64-69. doi:10.2478/jce-2021-0013  725. Perotti C, Baldanti F, Bruno R, et al. Mortality reduction in 46 patients with severe COVID-19 treated with hyperimmune plasma. A proof-of-concept, single-arm, multicenter trial. Article. *Haematologica*. 2020;105(12):2834-2840. doi:10.3324/haematol.2020.261784  726. Perren A, Cerutti B, Lepori M, et al. Influence of steroids on Procalcitonin and C-reactive protein in patients with COPD and community-acquired pneumonia. Article. *Infection*. 2008;36(2):163-166. doi:10.1007/s15010-007-7206-5  727. Picart J, Moiton MP, Gaüzère BA, Gazaille V, Combes X, DiBernardo S. Introduction of a PCT-based algorithm to guide antibiotic prescription in COPD exacerbation. Article. *Medecine et Maladies Infectieuses*. 2016;46(8):429-435. doi:10.1016/j.medmal.2016.07.008  728. Piechota M, Banach M, Irzmański R, et al. N-terminal brain natriuretic propeptide levels correlate with procalcitonin and C-reactive protein levels in septic patients. Article. *Cellular and Molecular Biology Letters*. 2007;12(2):162-175. doi:10.2478/s11658-006-0062-y  729. Pietri L, Giorgi R, Bégu A, et al. Excess body weight is an independent risk factor for severe forms of COVID-19. Article. *Metabolism: Clinical and Experimental*. 2021;117doi:10.1016/j.metabol.2021.154703  730. Pilgram L, Eberwein L, Wille K, et al. Clinical course and predictive risk factors for fatal outcome of SARS-CoV-2 infection in patients with chronic kidney disease. Article. *Infection*. 2021;49(4):725-737. doi:10.1007/s15010-021-01597-7  731. Pillai S, Lawrence M, Zaldua JC, et al. Relationship between the procalcitonin levels and clot microstructure in acute exacerbation of chronic obstructive pulmonary disease (AECOPD). Conference Abstract. *Critical Care*. 2022;26(SUPPL 1)doi:10.1186/s13054-022-03927-z  732. Piñeiro GJ, Molina-Andújar A, Hermida E, et al. Severe acute kidney injury in critically ill COVID-19 patients. Article. *Journal of Nephrology*. 2021;34(2):285-293. doi:10.1007/s40620-020-00918-7  733. Pinet C. Importance of procalcitonin in respiratory disease. Article. *Revue des Maladies Respiratoires*. 2006;23(5 C2):15S75-15S79. doi:10.1016/s0761-8425(06)72005-8  734. Pisi G, Olivieri D, Chetta A. The airway neurogenic inflammation: Clinical and pharmacological implications. Review. *Inflammation and Allergy - Drug Targets*. 2009;8(3):176-181. doi:10.2174/187152809788681047  735. Pitscheider L, Karolyi M, Burkert FR, et al. Muscle involvement in SARS-CoV-2 infection. Article. *European Journal of Neurology*. 2021;28(10):3411-3417. doi:10.1111/ene.14564  736. Piwowarczyk P, Szczukocka M, Kutnik P, et al. Risk factors and outcomes for acute respiratory failure in coronavirus disease 2019: An observational cohort study. Article. *Advances in Clinical and Experimental Medicine*. 2021;30(2)doi:10.17219/ACEM/130603  737. Pizzini A, Ziscka S, Sahanic A, et al. Diagnostic and prognostic value of inflammatory parameters including neopterin in pneumonia, COPD and acute exacerbations of COPD. Conference Abstract. *Pteridines*. 2016;27(1-2):46-47. doi:10.1515/pterid-2016-0002  738. Plebani M, Fabbri LM. Procalcitonin-guided antibiotic therapy: A potentially effective and efficient strategy. Editorial. *Clinical Chemistry and Laboratory Medicine*. 2015;53(4):519-520. doi:10.1515/cclm-2015-0061  739. Pletz MW, Rohde GG, Welte T, Kolditz M, Ott S. Advances in the prevention, management, and treatment of community-acquired pneumonia. Review. *F1000Research*. 2016;5doi:10.12688/f1000research.7657.1  740. Polat M, Salbaş ÇS, Sari S, Doğan M, Çam S, Karadağ A. The association between prognosis and sarcopenia assessed by psoas muscle measurements in elderly male patients with covid-19. Article. *Turk Geriatri Dergisi*. 2021;24(4):557-566. doi:10.31086/tjgeri.2021.253  741. Politis PA, Kallstrom G, Tan M, File TM. Early discontinuation of antibacterials is safe for patients with community-acquired pneumonia (CAP) who have a positive viral test, negative tests for bacteria, and low procalcitonin. Conference Abstract. *Open Forum Infectious Diseases*. 2019;6:S765. doi:10.1093/ofid/ofz360.1915  742. Polverino E, Torres A. Diagnostic strategies for healthcare- associated pneumonia. Review. *Seminars in Respiratory and Critical Care Medicine*. 2009;30(1):36-45. doi:10.1055/s-0028-1119807  743. Poropat G, Radovan A, Lackovic A, Vranic L, Hauser G, Stimac D. Use of balanced crytsalloid solutions in the early treatment of acute pancreatitis – protocol for a randomized controlled trial. Journal: Conference Abstract. *Pancreatology*. 2020;20:S77‐. doi:10.1016/j.pan.2020.07.115  744. Powrie DJ, Goodwin R, Allan-Smith D, et al. An integrated it system for COPD between primary, secondary and community care using systmone. Conference Abstract. *Thorax*. 2012;67:A188-A189. doi:10.1136/thoraxjnl-2012-202678.374  745. Pranata R, Huang I, Lim MA, Yonas E, Vania R, Kuswardhani RAT. Delirium and Mortality in Coronavirus Disease 2019 (COVID-19) – A Systematic Review and Meta-analysis. Article. *Archives of Gerontology and Geriatrics*. 2021;95doi:10.1016/j.archger.2021.104388  746. Prins HJ, Duijkers R, Kramer G, et al. Relationship between biomarkers and findings on low-dose computed tomography in hospitalised patients with acute exacerbation of COPD. *ERJ Open Res*. Apr 2022;8(2)doi:10.1183/23120541.00054-2022  747. Prins JM, Van Der Poll T. Is procalcitonin measurement useful in managing respiratory tract infections? Review. *Nederlands Tijdschrift voor Geneeskunde*. 2016;160(44)  748. Proboszcz M, Goryca K, Nejman-Gryz P, et al. Phenotypic variations of mild-to-moderate obstructive pulmonary diseases according to airway inflammation and clinical features. Article. *Journal of Inflammation Research*. 2021;14:2793-2806. doi:10.2147/JIR.S309844  749. Pullicino S, Debattista J, Gouder C, Montefort S. Infective triggers for asthma exacerbations in Malta. Article. *Malta Medical Journal*. 2022;34(2):65-77. doi:10.1183/13993003.congress-2020.2380  750. Qi L, Guo X, Nie C, Lv X, Zhang M. Research on Effects of Oropharyngeal Aspiration on Incidence of Ventilator-Associated Pneumonia in Patients with Cerebral Hemorrhage in ICU. Article. *Journal of Healthcare Engineering*. 2022;2022doi:10.1155/2022/6433666  751. Qian CL, Fan R. Effect of Pingchuan Guben decoction on patients with chronic obstructive pulmonary disease: Results from a randomized comparative effectiveness research trial. Article. *Experimental and Therapeutic Medicine*. 2017;14(4):3915-3925. doi:10.3892/etm.2017.5018  752. Qian W, Huang GZ. Neutrophil CD64 as a Marker of Bacterial Infection in Acute Exacerbations of Chronic Obstructive Pulmonary Disease. Article. *Immunological Investigations*. 2016;45(6):490-503. doi:10.1080/08820139.2016.1177540  753. Qian Y, Yu K, Xu K, Wang R. Benefit of Nadroparin in acute exacerbation among COPD patients. Journal: Conference Abstract. *Respirology*. 2011;16(Suppl 2):98. doi:10.1111/j.1400-1843.2011.02071.x  754. Quenot JP, Luyt CE, Roche N, et al. Role of biomarkers in the management of antibiotic therapy: An expert panel review II: Clinical use of biomarkers for initiation or discontinuation of antibiotic therapy. Review. *Annals of Intensive Care*. 2013;3(1):1-17. doi:10.1186/2110-5820-3-21  755. Raasveld SJ, Delnoij TSR, Broman LM, et al. Extracorporeal Membrane Oxygenation in Patients With COVID-19: An International Multicenter Cohort Study. Article. *Journal of Intensive Care Medicine*. 2021;36(8):910-917. doi:10.1177/08850666211007063  756. Rai DK, Sahay N, Lohani P. Clinical characteristics and treatment outcomes of 293 COVID-19 patients admitted to the intensive care unit of a tertiary care hospital of eastern India. Article. *Indian Journal of Critical Care Medicine*. 2021;25(12):1395-1401. doi:10.5005/jp-journals-10071-24048  757. Raiten DJ, Sakr Ashour FA, Ross AC, et al. Inflammation and nutritional science for programs/policies and interpretation of research evidence (INSPIRE). Article. *Journal of Nutrition*. 2015;145(5):1039S-1108S. doi:10.3945/jn.114.194571  758. Ramage G, Culshaw S, Jones B, Williams C. Are we any closer to beating the biofilm: Novel methods of biofilm control. Review. *Current Opinion in Infectious Diseases*. 2010;23(6):560-566. doi:10.1097/QCO.0b013e32833e5850  759. Ramazan G, Basar C, Burcu GY, Ramazan U, Ertugrul A, Salih F. The experience of an emergency intensive care unit during the COVID-19 pandemic: A retrospective cohort study. Article. *Annals of Clinical and Analytical Medicine*. 2021;12:S423-S427. doi:10.4328/ACAM.20622  760. Ramirez P, Menendez R, Torres A. Biomarkers and pneumonia: Should we all be using them? Article. *Current Respiratory Medicine Reviews*. 2011;7(4):262-267. doi:10.2174/157339811798281287  761. Ranard BL, Megjhani M, Terilli K, et al. Identification of Endotypes of Hospitalized COVID-19 Patients. Article. *Frontiers in medicine*. 2021;8doi:10.3389/fmed.2021.770343  762. Reale ML, Bironzo P, Bertaglia V, et al. SARS-CoV-2 Infection in Cancer Patients: A Picture of an Italian Onco-Covid Unit. Article. *Frontiers in Oncology*. 2020;10doi:10.3389/fonc.2020.01722  763. Reinhart K, Hartog CS. Biomarkers as a guide for antimicrobial therapy. Article. *International Journal of Antimicrobial Agents*. 2010;36(SUPPL. 2):S17-S21. doi:10.1016/j.ijantimicag.2010.11.009  764. Rello J, Bello I, de Vicente R, et al. Risk Factors for Mortality in 272 Patients With Lung Transplant: A Multicenter Analysis of 7 Intensive Care Units. *Arch Bronconeumol*. Aug 2017;53(8):421-426. doi:10.1016/j.arbres.2016.12.019  765. Rello J, Bello I, de Vicente R, et al. Risk Factors for Mortality in 272 Patients With Lung Transplant: A Multicenter Analysis of 7 Intensive Care Units. Article. *Archivos de Bronconeumologia*. 2017;53(8):421-426. doi:10.1016/j.arbres.2016.12.019  766. Ren Q, Liu H, Wang Y, et al. The Role of Red Blood Cell Distribution Width in the Severity and Prognosis of Community-Acquired Pneumonia. Article. *Canadian Respiratory Journal*. 2021;2021doi:10.1155/2021/8024024  767. Rendon A, Rendon-Ramirez EJ, Rosas-Taraco AG. Relevant Cytokines in the Management of Community-Acquired Pneumonia. Review. *Current Infectious Disease Reports*. 2016;18(3):1-9. doi:10.1007/s11908-016-0516-y  768. Rhee C. Using procalcitonin to guide antibiotic therapy. Review. *Open Forum Infectious Diseases*. 2017;4(1)doi:10.1093/ofid/ofw249  769. Rinaudo M, Ferrer M, Terraneo S, et al. Impact of COPD in the outcome of ICU - Acquired pneumonia with and without previous intubation. Article. *Chest*. 2015;147(6):1530-1538. doi:10.1378/chest.14-2005  770. Ringe JD. Glucocorticoid-induced osteoporosis. Clinical Trial; Journal Article; Randomized Controlled Trial; Review. *Clinical rheumatology*. 1989;8 Suppl 2:109‐115. doi:10.1007/BF02207244  771. Ringe JD, Welzel D. Salmon calcitonin in the therapy of corticoid-induced osteoporosis. Clinical Trial; Controlled Clinical Trial; Journal Article; Randomized Controlled Trial. *European journal of clinical pharmacology*. 1987;33(1):35‐39. doi:10.1007/BF00610377  772. Río Ramírez MT, Juretschke Moragues MA, Fernández González R, et al. Value of Exhaled Nitric Oxide (FeNO) And Eosinophilia During the Exacerbations of Chronic Obstructive Pulmonary Disease Requiring Hospital Admission. Article. *COPD: Journal of Chronic Obstructive Pulmonary Disease*. 2018;15(4):369-376. doi:10.1080/15412555.2018.1482532  773. Rios-Toro JJ, Pola-Gallego de Guzman MD, Guerrero-Marin M, et al. Prognostic Value of Variations in Serum Biomarkers and Prognostic Scores Values Between Admission and Second Day in Intensive Care Unit Septic Patients. *Cureus*. Jul 2021;13(7):e16472. doi:10.7759/cureus.16472  774. Rizwan R, Feuerstadt P. Bad blood: Ischemic conditions of the large bowel. Review. *Current Opinion in Gastroenterology*. 2022;38(1):72-79. doi:10.1097/MOG.0000000000000797  775. Rodríguez A, Ferri C, Martin-Loeches I, et al. Risk factors for noninvasive ventilation failure in critically ill subjects with confirmed influenza infection. Article. *Respiratory Care*. 2017;62(10):1307-1315. doi:10.4187/respcare.05481  776. Rodriguez W, Candelario A, Otero-Dominguez Y, Torres-Palacios J. A not so typical atypical mycobacterial infection: Pulmonary mycobacterial infection with mycobacterium marseillense. Conference Abstract. *American Journal of Respiratory and Critical Care Medicine*. 2019;199(9)  777. Rola P, Doroszko A, Trocha M, et al. Sex-Dependent Differences in Predictive Value of the C2HEST Score in Subjects with COVID-19—A Secondary Analysis of the COLOS Study. Article. *Viruses*. 2022;14(3)doi:10.3390/v14030628  778. Ronderos D, Omar AMS, Abbas H, et al. Chronic hepatitis-C infection in COVID-19 patients is associated with in-hospital mortality. Article. *World Journal of Clinical Cases*. 2021;9(29):8749-8762. doi:10.12998/wjcc.v9.i29.8749  779. Rothberg MB, Pekow PS, Lindenauer PK. In reply. Letter. *JAMA - Journal of the American Medical Association*. 2010;304(12):1326-1327. doi:10.1001/jama.2010.1340  780. Roy K, Marau A, Esmond G, et al. Home based respiratory point of care testing (R-POCTc) to improve the diagnosis and management of COPD exacerbations in the community. Conference Abstract. *Thorax*. 2019;74:A66. doi:10.1136/thorax-2019-BTSabstracts2019.110  781. Roy K, Marau A, Gora L, Esmond G, Ciobanu C, Buxton M. Respiratory point of care testing (R-POCT) to facilitate diagnosis and treatment in the community for COPD exacerbations. Conference Abstract. *European Respiratory Journal*. 2019;54doi:10.1183/13993003.congress-2019.PA4241  782. Runzheimer J, Mewes C, Büttner B, et al. Lack of an association between the functional polymorphism TREM-1 rs2234237 and the clinical course of sepsis among critically ill caucasian patients—A monocentric prospective genetic association study. Article. *Journal of Clinical Medicine*. 2019;8(3)doi:10.3390/jcm8030301  783. Russo A, Bassetti M, Bellelli V, et al. Efficacy of a Fosfomycin-Containing Regimen for Treatment of Severe Pneumonia Caused by Multidrug-Resistant Acinetobacter baumannii: A Prospective, Observational Study. Article. *Infectious Diseases and Therapy*. 2021;10(1):187-200. doi:10.1007/s40121-020-00357-8  784. Russo A, Cacciola E, Borrazzo C, et al. Clinical characteristics and outcome of patients with suspected covid-19 in emergency department (Resiliency study ii). Article. *Diagnostics*. 2021;11(8)doi:10.3390/diagnostics11081368  785. Sabaz MS, Aşar S. FACTORS ASSOCIATED WITH PROLONGED MECHANICAL VENTILATION IN THE GERIATRIC POPULATION AND ITS EFFECT ON PATIENT OUTCOMES. Article. *Turk Geriatri Dergisi*. 2021;24(4):463-477. doi:10.31086/tjgeri.2021.244  786. Sabetian G, Feiz F, Shakibafard A, et al. Challenges of diagnosis of COVID-19 in trauma patients: A case series. Article. *Trauma (United Kingdom)*. 2021;23(3):218-229. doi:10.1177/1460408620950602  787. Sabrine N, Nejla T, Habiba S, et al. Procalcitonin guided antibiotic therapy during severe acute exacerbation of chronic obstructive pulmonary disease requiring mechanical ventilation: A before after study. Conference Abstract. *Annals of Intensive Care*. 2018;8(1)doi:10.1186/s13613-017-0345-7  788. Sadigov A, Khoshbanyani P, Mirzoyeva V, Bagirzadeh A. PREDICTING OF MORTALITY FOR PATIENTS WITH COVID-19 ADMITTED TO ICU: A RETROSPECTIVE ANALYSIS OF UNIVERSITY MONO-CENTRIC DATA. Conference Abstract. *Chest*. 2021;160(4):A1007. doi:10.1016/j.chest.2021.07.937  789. Saeed MA, Adickes ED, Romberger DJ. Blasts in the lung: Rare presentation of acute myeloid leukemia. Conference Abstract. *American Journal of Respiratory and Critical Care Medicine*. 2018;197(MeetingAbstracts)  790. Safarika A, Wacker JW, Katsaros K, et al. A 29-mRNA host response test from blood accurately distinguishes bacterial and viral infections among emergency department patients. *Intensive Care Med Exp*. Jun 18 2021;9(1):31. doi:10.1186/s40635-021-00394-8  791. Sager R, Kutz A, Mueller B, Schuetz P. Procalcitonin-guided diagnosis and antibiotic stewardship revisited. *BMC Med*. Jan 24 2017;15(1):15. doi:10.1186/s12916-017-0795-7  792. Şahin F, Koşar AF, Aslan AF, Yiǧitbaş B, Uslu B. Serum biomarkers in patients with stable and acute exacerbation of chronic obstructive pulmonary disease: A comparative study. Article. *Journal of Medical Biochemistry*. 2019;38(4):503-511. doi:10.2478/jomb-2018-0050  793. Saib A, Amara W, Wang P, et al. Lack of efficacy of hydroxychloroquine and azithromycin in patients hospitalized for covid-19 pneumonia: A retrospective study. Article. *PLoS ONE*. 2021;16(6 June)doi:10.1371/journal.pone.0252388  794. Saleem A. Serum procalcitonin in lower respiratory tract infections in adult patients. Review. *British Journal of Medical Practitioners*. 2019;12(2)  795. Salgado-Aranda R, Pérez-Castellano N, Núñez-Gil I, et al. Influence of Baseline Physical Activity as a Modifying Factor on COVID-19 Mortality: A Single-Center, Retrospective Study. Article. *Infectious Diseases and Therapy*. 2021;10(2):801-814. doi:10.1007/s40121-021-00418-6  796. Salmaggi C, Ancona F, Olivetti J, Pagliula G, Ramirez GA. Raoultella planticola-associated cholangitis and sepsis: A case report and literature review. Article. *QJM*. 2014;107(11):911-913. doi:10.1093/qjmed/hcu087  797. Samantaray S, Karan P, Sharma A, et al. Prevalence, Presentation and Outcome of Secondary Bloodstream Infections among COVID-19 Patients. Article. *Infectious Disorders - Drug Targets*. 2022;22(5)doi:10.2174/1871526522666220418093450  798. Samantaray S, Karan P, Sharma A, et al. Prevalence, presentation and outcome of secondary bloodstream infections among the COVID-19 patients. *Infectious disorders drug targets*. Apr 18 2022;doi:10.2174/1871526522666220418093450  799. Saminan S, Julisafrida L, Ridwan M, Fajri N. COVID-19 Pandemic: What Considerations Should Be Taken during the Assessment and Management of COPD Exacerbation? Review. *Open Access Macedonian Journal of Medical Sciences*. 2022;10:71-80. doi:10.3889/oamjms.2022.7930  800. Sampson F, O'Cathain A, Strong M, Pickin M, Esmonde L. Commissioning processes in primary care trusts: A repeated cross-sectional survey of health care commissioners in England. Article. *Journal of Health Services Research and Policy*. 2012;17(SUPPL. 1):31-39. doi:10.1258/jhsrp.2011.010191  801. Sánchez Cadena AD, Negreira Caamaño M, Pérez Serrano R, Porras Leal ML. Intravenous immunoglobulins: A therapeutic alternative to consider in kidney transplant patients with COVID-19. Letter. *Nefrologia*. 2021;41(2):220-222. doi:10.1016/j.nefroe.2021.04.001  802. Sandifer JP, Jones AE. Can procalcitonin levels guide antibiotic therapy in bacterial infections and reduce antibiotic overconsumption without having a negative effect on clinical outcomes? Editorial. *Annals of Emergency Medicine*. 2012;60(3):370-371. doi:10.1016/j.annemergmed.2012.01.007  803. Şanli DET, Yildirim D. A new imaging sign in COVID-19 pneumonia: Vascular changes and their correlation with clinical severity of the disease. Article. *Diagnostic and Interventional Radiology*. 2021;27(2):172-180. doi:10.5152/dir.2020.20346  804. Santino I, Alari A, Bono S, et al. Saccharomyces cerevisiae fungemia, a possible consequence of the treatment of Clostridium difficile colitis with a probioticum. Article. *International Journal of Immunopathology and Pharmacology*. 2014;27(1):143-146. doi:10.1177/039463201402700120  805. Sartini M, Del Puente F, Oliva M, et al. Riding the covid waves: Clinical trends, outcomes, and remaining pitfalls of the sars-cov-2 pandemic: An analysis of two high-incidence periods at a hospital in Northern Italy. Article. *Journal of Clinical Medicine*. 2021;10(22)doi:10.3390/jcm10225239  806. Savage TJ, Pearce HL, Lockett J, Chapman SR. Health information for commissioners: Asthma & chronic obstructive pulmonary disease (COPD). Conference Abstract. *Pharmacoepidemiology and Drug Safety*. 2010;19(6):656-657. doi:10.1002/pds  807. Savelikhina I, Ostrovskyy M, Ostrovska K, Kulynych-Miskiv M, Varunkiv O, Shvets K. Biomarkers in COPD: Beneficial effects of roflumilast. Conference Abstract. *European Respiratory Journal*. 2017;50doi:10.1183/1393003.congress-2017.PA1071  808. Savelikhina I, Ostrovskyy M, Ostrovska K, Varunkiv O, Kulynych-Miskiv M, Shvets K. The role of biomarkers and effectiveness of maintenance therapy in stable severe COPD. Conference Abstract. *European Respiratory Journal*. 2017;50doi:10.1183/1393003.congress-2017.PA1072  809. Sayin Gülensoy E, Yüksel A, Ogan N, Umudum H, Akpinar EE. Subacute lung injury associated with heated tobacco products. Article. *Duzce Medical Journal*. 2021;23(2):218-220. doi:10.18678/dtfd.896093  810. Sayit AT, Elmali M, Deveci A, Gedikli O. Relationship between acute phase reactants and prognosis in patients with or without covid-19 pneumonia. Article. *Revista do Instituto de Medicina Tropical de Sao Paulo*. 2021;63doi:10.1590/S1678-9946202163051  811. Schaaf B, Rupp J. Diagnosis of community-acquired pneumonia. Review. *Pneumologie*. 2006;60(9):547-558. doi:10.1055/s-2006-932223  812. Scheinpflug K, Schalk E, Grabert E, Jost Achenbach H. Procalcitonin is not useful to discriminate between infectious and noninfectious CRP elevation in patients with non–small cell lung cancer. Letter. *Infection Control and Hospital Epidemiology*. 2015;36(9):1117-1118. doi:10.1017/ice.2015.134  813. Scherr A, Blasi F, Stolz D. An update on exacerbation in COPD. Review. *Minerva Pneumologica*. 2011;50(2):129-146.  814. Schroeder M, Schaumburg B, Mueller Z, et al. High estradiol and low testosterone levels are associated with critical illness in male but not in female COVID-19 patients: a retrospective cohort study. Article. *Emerging Microbes and Infections*. 2021;10(1):1807-1818. doi:10.1080/22221751.2021.1969869  815. Schuetz P, Beishuizen A, Broyles M, et al. Procalcitonin (PCT)-guided antibiotic stewardship: An international experts consensus on optimized clinical use. Article. *Clinical Chemistry and Laboratory Medicine*. 2019;57(9):1308-1318. doi:10.1515/cclm-2018-1181  816. Schuetz P, Birkhahn R, Sherwin R, et al. Serial procalcitonin predicts mortality in severe sepsis patients: results from the multicenter procalcitonin monitoring SEpsis (MOSES) Study. Journal: Article. *Critical care medicine*. 2017;45(5):781‐789. doi:10.1097/CCM.0000000000002321  817. Schuetz P, Birkhahn R, Sherwin R, et al. Serial procalcitonin predicts mortality in severe sepsis patients: Results from the multicenter procalcitonin monitoring SEpsis (MOSES) Study. Article. *Critical Care Medicine*. 2017;45(5):781-789. doi:10.1097/CCM.0000000000002321  818. Schuetz P, Bolliger R, Merker M, et al. Procalcitonin-guided antibiotic therapy algorithms for different types of acute respiratory infections based on previous trials. *Expert Rev Anti Infect Ther*. Jul 2018;16(7):555-564. doi:10.1080/14787210.2018.1496331  819. Schuetz P, Bolliger R, Merker M, et al. Procalcitonin-guided antibiotic therapy algorithms for different types of acute respiratory infections based on previous trials. Review. *Expert Review of Anti-Infective Therapy*. 2018;16(7):555-564. doi:10.1080/14787210.2018.1496331  820. Schuetz P, Briel M, Mueller B. Clinical outcomes associated with procalcitonin algorithms to guide antibiotic therapy in respiratory tract infections. Journal: Note. *JAMA - journal of the american medical association*. 2013;309(7):717‐718. doi:10.1001/jama.2013.697  821. Schuetz P, Chiappa V, Briel M, Greenwald JL. Procalcitonin algorithms for antibiotic therapy decisions: A systematic review of randomized controlled trials and recommendations for clinical algorithms. Review. *Archives of Internal Medicine*. 2011;171(15):1322-1331. doi:10.1001/archinternmed.2011.318  822. Schuetz P, Christ-Crain M, Albrich W, Zimmerli W, Mueller B. Guidance of antibiotic therapy with procalcitonin in lower respiratory tract infections: Insights into the ProHOSP study. Article. *Virulence*. 2010;1(2):88-92. doi:10.4161/viru.1.2.10488  823. Schuetz P, Christ-Crain M, Müller B. Procalcitonin and other biomarkers to improve assessment and antibiotic stewardship in infections - Hope for hype? Review. *Swiss Medical Weekly*. 2009;139(23-24):318-326.  824. Schuetz P, Christ-Crain M, Thomann R, et al. Effect of procalcitonin-based guidelines vs standard guidelines on antibiotic use in lower respiratory tract infections: the ProHOSP randomized controlled trial. Comparative Study; Journal Article; Multicenter Study; Randomized Controlled Trial; Research Support, Non‐U.S. Gov't. *JAMA*. 2009;302(10):1059‐1066. doi:10.1001/jama.2009.1297  825. Schuetz P, Christ-Crain M, Thomann R, et al. Effect of procalcitonin-based guidelines vs standard guidelines on antibiotic use in lower respiratory tract infections: The ProHOSP randomized controlled trial. Article. *JAMA - Journal of the American Medical Association*. 2009;302(10):1059-1066. doi:10.1001/jama.2009.1297  826. Schuetz P, Christ-Crain M, Thomann R, et al. Effect of procalcitonin-based guidelines compared with standard guidelines on antibiotic use in lower respiratory tract infections: The randomized-controlled multicenter ProHOSP trial. Conference Abstract. *Critical Care*. 2009;13:S157. doi:10.1186/cc7550  827. Schuetz P, Christ-Crain M, Wolbers M, et al. Procalcitonin guided antibiotic therapy and hospitalization in patients with lower respiratory tract infections: a prospective, multicenter, randomized controlled trial. Journal Article; Multicenter Study; Randomized Controlled Trial; Research Support, Non‐U.S. Gov't. *BMC health services research*. 2007;7:102. doi:10.1186/1472-6963-7-102  828. Schuetz P, Christ-Crain M, Wolbers M, et al. Procalcitonin guided antibiotic therapy and hospitalization in patients with lower respiratory tract infections: a prospective, multicenter, randomized controlled trial. *BMC Health Serv Res*. Jul 5 2007;7:102. doi:10.1186/1472-6963-7-102  829. Schuetz P, Leykum LK. Procalcitonin. Review. *Hospital Medicine Clinics*. 2012;1(3):e301-e312. doi:10.1016/j.ehmc.2012.04.005  830. Schuetz P, Mueller B. Biomarker-guided de-escalation of empirical therapy is associated with lower risk for adverse outcomes. Letter. *Intensive Care Medicine*. 2014;40(1):141. doi:10.1007/s00134-013-3139-x  831. Schuetz P, Wirz Y, Mueller B. Procalcitonin testing to guide antibiotic therapy in acute upper and lower respiratory tract infections. Review. *JAMA - Journal of the American Medical Association*. 2018;319(9):925-926. doi:10.1001/jama.2018.0852  832. Schuetz P, Wirz Y, Sager R, et al. Procalcitonin to initiate or discontinue antibiotics in acute respiratory tract infections. Review. *Cochrane Database of Systematic Reviews*. 2017;2017(10)doi:10.1002/14651858.CD007498.pub3  833. Schuetz P, Wolbers M, Christ-Crain M, et al. Prohormones for prediction of adverse medical outcome in community-acquired pneumonia and lower respiratory tract infections. Journal: Article. *Critical care (London, England)*. 2010;14(3)doi:10.1186/cc9055  834. Schuetz P, Wolbers M, Christ-Crain M, et al. Prohormones for prediction of adverse medical outcome in community-acquired pneumonia and lower respiratory tract infections. Article. *Critical Care*. 2010;14(3)doi:10.1186/cc9055  835. Schult D, Reitmeier S, Koyumdzhieva P, et al. Gut bacterial dysbiosis and instability is associated with the onset of complications and mortality in COVID-19. Article. *Gut Microbes*. 2022;14(1)doi:10.1080/19490976.2022.2031840  836. Scott R, Sage B, Cheah K, Vancheeswaran R, Mack DJ. The use of procalcitonin to guide antimicrobial use for respiratory tract infections in a district general hospital: A feasibility study. Conference Abstract. *Clinical Microbiology and Infection*. 2011;17:S453-S454. doi:10.1111/j.1469-0691.2011.03558.x  837. Seeßle J, Hippchen T, Schnitzler P, Gsenger J, Giese T, Merle U. High rate of HSV-1 reactivation in invasively ventilated COVID-19 patients: Immunological findings. Article. *PLoS ONE*. 2021;16(7 July)doi:10.1371/journal.pone.0254129  838. Şenol A, Gündağ Ö, Özer Balin Ş, Aşçi Toraman Z, Gül Y, Yildirim A. The importance of hematological and demographic characteristics in the course of COVID-19 pneumonia. Article. *Flora*. 2021;26(3):460-467. doi:10.5578/FLORA.20219714  839. Şenol E, Çilli A, Günen H, et al. The role of pneumococcal pneumonia among community-acquired pneumonia in adult turkish population: Turkcap study. Article. *Turkish Thoracic Journal*. 2021;22(4):339-345. doi:10.5152/TurkThoracJ.2021.20223  840. Seo H, Cha SI, Shin KM, et al. Clinical relevance of emphysema in patients hospitalized with community-acquired pneumonia: Clinical features and prognosis. Article. *Clinical Respiratory Journal*. 2021;15(7):826-834. doi:10.1111/crj.13370  841. Seo K, Kitazawa T, Yoshino Y, Koga I, Ota Y. Characteristics of serum endocan levels in infection. Article. *PLoS ONE*. 2015;10(4)doi:10.1371/journal.pone.0123358  842. Serrao S, Jabo B, Shah C, Jackson C. Risk factors associated with mortality in hospitalized patients with gastroduodenal angioectasia using data from the national inpatient sample. Conference Abstract. *American Journal of Gastroenterology*. 2013;108:S93. doi:10.1038/ajg.2013.264  843. Serrao S, Jabo B, Shah C, Jackson C. Factors associated with mortality in patients with intestinal angioectasia (IA) and blood loss using a population-based inpatient sample. Conference Abstract. *American Journal of Gastroenterology*. 2013;108:S168. doi:10.1038/ajg.2013.266  844. Sezer R, Esendagli D, Erol C, Hekimoglu K. New challenges for management of COVID-19 patients: Analysis of MDCT based “Automated pneumonia analysis program”. Article. *European Journal of Radiology Open*. 2021;8doi:10.1016/j.ejro.2021.100370  845. Shah C, Grando DJ, Rainess RA, et al. Factors associated with increased mortality in hospitalized COVID-19 patients. Article. *Annals of Medicine and Surgery*. 2020;60:308-313. doi:10.1016/j.amsu.2020.10.071  846. Shah N, Patel R, Jain R. NOTHING “GOOD” ABOUT IT: A CASE OF GOODPASTURE SYNDROME PRESENTING AS DIFFUSE ALVEOLAR HEMORRHAGE. Conference Abstract. *Chest*. 2019;156(4):A1540. doi:10.1016/j.chest.2019.08.1359  847. Shakespeare J, Storey K, Parr D. An audit of the effectiveness of competency based spirometry training. Conference Abstract. *Thorax*. 2011;66:A161. doi:10.1136/thoraxjnl-2011-201054c.228  848. Shang L, Xi AQ, Zhu AQ. Correlation between chronic obstructive pulmonary disease and arterial stiffness in elderly in plateau area. Conference Abstract. *Journal of the American Geriatrics Society*. 2019;67:S660. doi:10.1111/jgs.16120  849. Shao L, Li X, Zhou Y, et al. Novel Insights Into Illness Progression and Risk Profiles for Mortality in Non-survivors of COVID-19. Article. *Frontiers in medicine*. 2020;7doi:10.3389/fmed.2020.00246  850. Sharma BB, Singh V. Indian pneumonia guidelines. Editorial. *Lung India*. 2012;29(4):307-308. doi:10.4103/0970-2113.102793  851. Shehabi Y, Seppelt I. Pro/Con debate: Is procalcitonin useful for guiding antibiotic decision making in critically ill patients? Review. *Critical Care*. 2008;12(3)doi:10.1186/cc6860  852. Sheng X, Zhao C, Wang Z. The relationship between induced sputum cell counts and lung function in patients hospitalized for chronic obstructive pulmonary disease. Conference Abstract. *Respirology*. 2011;16:118. doi:10.1111/j.1400-1843.2011.02071.x  853. Sheng XM, Wang ZS, Zhao CY. Relationship between induced sputum cell counts and lung function in patients hospitalized for chronic obstructive pulmonary disease. Article. *Journal of Dalian Medical University*. 2014;36(2):164-167+181. doi:10.11724/jdmu.2014.02.15  854. Shi B, Shi F, Xu K, et al. The prognostic performance of Sepsis-3 and SIRS criteria for patients with urolithiasis-associated sepsis transferred to ICU following surgical interventions. Article. *Experimental and Therapeutic Medicine*. 2019;18(5):4165-4172. doi:10.3892/etm.2019.8057  855. Shim JJ, Choi J, Chung S, et al. Combined serum eosinophil count and procalcitonin level as a marker of bacterial infection in acute exacerbation of chronic obstructive pulmonary disease. Conference Abstract. *European Respiratory Journal*. 2016;48doi:10.1183/13993003.congress-2016.PA1004  856. Shu B, Li H, Zhou X, Ding Z, Wan L. Efficacy and Safety of Re Du Ning Injection for Acute Exacerbations of Chronic Obstructive Pulmonary Disease: A Systematic Review and Meta-Analysis. Review. *Evidence-based Complementary and Alternative Medicine*. 2022;2022doi:10.1155/2022/7479639  857. Sialer S, Adamantia L, Guerrero M, Torres A. Relation between chronic obstructive pulmonary disease and antibiotics. Article. *Current Infectious Disease Reports*. 2012;14(3):300-307. doi:10.1007/s11908-012-0255-7  858. Siddiqi A, Sethi S. Optimizing antibiotic selection in treating COPD exacerbations. Review. *International Journal of COPD*. 2008;3(1):31-44.  859. Signes-Costa J, Núñez-Gil IJ, Soriano JB, et al. Prevalence and 30-Day Mortality in Hospitalized Patients With Covid-19 and Prior Lung Diseases. Article. *Archivos de Bronconeumologia*. 2021;57:13-20. doi:10.1016/j.arbres.2020.11.012  860. Silverio R, Sequeira M, Bastos M. The value of procalcitonin in guiding antibiotic use in lower respiratory tract infection in elderly patients. Conference Abstract. *European Journal of Internal Medicine*. 2013;24:e60-e61. doi:10.1016/j.ejim.2013.08.147  861. Singh H, Handa R, Kak V, Wasilewski A. Complex encephalopathy arising from the combination of opioids and gabapentin. Review. *Drug and Therapeutics Bulletin*. 2019;57(8):125-127. doi:10.1136/dtb.2019.228354rep  862. Sintes H, Sibila O, Waterer GW, Chalmers JD. Severity assessment tools in CAP. 2014. p. 88-104.  863. Sıpahı S, Dheır H, Toçoğlu A, et al. Characteristics and mortality determinants of COVID-19 patients undergoing hemodialysis. Article. *Turkish Journal of Medical Sciences*. 2021;51(2):421-427. doi:10.3906/sag-2006-54  864. Sivapalan P, Jensen JU. Biomarkers in Chronic Obstructive Pulmonary Disease: Emerging Roles of Eosinophils and Procalcitonin. Article in Press. *Journal of innate immunity*. 2021;doi:10.1159/000517161  865. Sjoding MW, Valley TS, Prescott HC, Iwashyna TJ. Treatment uncertainty in patients with acute respiratory failure is common and associated with increased mortality. Conference Abstract. *American Journal of Respiratory and Critical Care Medicine*. 2019;199(9)  866. Ślusarczyk A, Piechocki K, Symonides B. Dyspnoe, cough and fever in 80-year old patient with chronic heart failure. Article. *Polski merkuriusz lekarski : organ Polskiego Towarzystwa Lekarskiego*. 2019;47(281):187-189.  867. Snell K, Breen C, Herring M, Maguire K. Roadmap to prevention: Identifiable traits linked to readmission within 90 days of a COPD exacerbation. Conference Abstract. *Chest*. 2017;152(4):A777. doi:10.1016/j.chest.2017.08.808  868. Soler N. Systemic markers of exacerbated chronic obstructive pulmonary disease: How they can help with the decision of whether or not to prescribe antibiotics. Editorial. *Archivos de Bronconeumologia*. 2008;44(11):581-583. doi:10.1016/S1579-2129(08)60109-3  869. Soler N, Esperatti M, Ewig S, Huerta A, Agusti C, Torres A. Sputum purulence-guided antibiotic use in hospitalised patients with exacerbations of COPD. Journal: Article. *European respiratory journal*. 2012;40(6):1344‐1353. doi:10.1183/09031936.00150211  870. Soler N, Esperatti M, Huerta AH, et al. Sputum purulence guided antibiotic therapy in patients with severe exacerbations of COPD. Conference Abstract. *American Journal of Respiratory and Critical Care Medicine*. 2010;181(1)  871. Song J, Zeng M, Wang H, et al. Distinct effects of asthma and COPD comorbidity on disease expression and outcome in patients with COVID-19. Article. *Allergy: European Journal of Allergy and Clinical Immunology*. 2021;76(2):483-496. doi:10.1111/all.14517  872. Song W, Wang Y, Tian F, et al. Clinical Significance of Procalcitonin, C-Reactive Protein, and Interleukin-6 in Helping Guide the Antibiotic Use for Patients with Acute Exacerbations of Chronic Obstructive Pulmonary Disease. Article. *Disease markers*. 2021;2021doi:10.1155/2021/8879401  873. Soni SL, Kajal K, Yaddanapudi LN, et al. Demographic & clinical profile of patients with covid-19 at a tertiary care hospital in north india. Article. *Indian Journal of Medical Research, Supplement*. 2021;153(1-2):115-125. doi:10.4103/ijmr.IJMR_2311_20  874. Soyler CA, Tanriover MD, Ascioglu S, Aksu NM, Arici M. Differentiation of acute decompensated heart failure and pneumonia in emergency department. Conference Abstract. *European Journal of Internal Medicine*. 2013;24:e196. doi:10.1016/j.ejim.2013.08.502  875. Sparano C, Zago E, Morettini A, et al. Euthyroid sick syndrome as an early surrogate marker of poor outcome in mild SARS-CoV-2 disease. Article. *Journal of Endocrinological Investigation*. 2022;45(4):837-847. doi:10.1007/s40618-021-01714-1  876. Spiegler P. Using procalcitonin to refine treatment of chronic obstructive pulmonary disease exacerbations. Note. *Clinical Pulmonary Medicine*. 2007;14(3):184-185. doi:10.1097/CPM.0b013e3180550295  877. Spiegler P. Do antibiotics for copd exacerbations improve outcomes? Note. *Clinical Pulmonary Medicine*. 2009;16(4):226-227. doi:10.1097/CPM.0b013e3181ad2a88  878. Spoto S, Daniel Markley J, Valeriani E, et al. Active Surveillance Cultures and Procalcitonin in Combination With Clinical Data to Guide Empirical Antimicrobial Therapy in Hospitalized Medical Patients With Sepsis. Article. *Frontiers in Microbiology*. 2022;13doi:10.3389/fmicb.2022.797932  879. Stephenson P, Sheikh A. Editors' choice. Editorial. *Primary Care Respiratory Journal*. 2011;20(4):1. doi:10.4104/pcrj.2011.00109  880. Stettmeier K, Manek G, Datta D. HERPES SIMPLEX PNEUMONIA IN AN IMMUNOCOMPETENT PATIENT WITH ARDS. Conference Abstract. *Chest*. 2019;156(4):A683-A684. doi:10.1016/j.chest.2019.08.662  881. Steuten L, Mewes J, Lepage-Nefkens I, Vrijhoef H. Is Procalcitonin Biomarker-Guided Antibiotic Therapy a Cost-Effective Approach to Reduce Antibiotic Resistant and Clostridium difficile Infections in Hospitalized Patients? Article. *OMICS A Journal of Integrative Biology*. 2018;22(9):616-625. doi:10.1089/omi.2018.0040  882. Stevenson R, Angus R, Blanchard M, et al. Intermediate care - Hospital-at-Home in chronic obstructive pulmonary disease: British Thoracic Society guideline. Article. *Thorax*. 2007;62(3):200-210. doi:10.1136/thx.2006.064931  883. Stockley RA. Sputum colour: A marker of bacterial infection. 2013. p. 27-33.  884. Stoeckle K, Johnston CD, Jannat-Khah DP, et al. COVID-19 in hospitalized adults with HIV. Article. *Open Forum Infectious Diseases*. 2020;7(8)doi:10.1093/ofid/ofaa327  885. Stojanovic I, Schneider JE, Wei L, Hong Z, Keane C, Schuetz P. Economic evaluation of procalcitonin-guided antibiotic therapy in acute respiratory infections: A Chinese hospital system perspective. Article. *Clinical Chemistry and Laboratory Medicine*. 2017;55(4):561-570. doi:10.1515/cclm-2016-0349  886. Stolz D, Boersma W, Louis R, et al. Association of the severe, frequent exacerbation phenotype with exercise capacity, sputum bacteriology and copeptin circulating levels in COPD. Conference Abstract. *European Respiratory Journal*. 2011;38  887. Stolz D, Breidthardt T, Christ-Crain M, et al. Use of B-type natriuretic peptide in the risk stratification of acute exacerbations of COPD. Comparative Study; Journal Article; Randomized Controlled Trial; Research Support, Non‐U.S. Gov't. *Chest*. 2008;133(5):1088‐1094. doi:10.1378/chest.07-1959  888. Stolz D, Christ-Crain M, Bingisser R, et al. Procalcitonin-guided antibiotic therapy in acute exacerbations of COPD (AECOPD): a randomised trial - the ProCOLD study. *European respiratory journal*. 2005;26(Suppl 49):Abstract No. 3044.  889. Stolz D, Christ-Crain M, Bingisser R, et al. Antibiotic treatment of exacerbations of COPD: a randomized, controlled trial comparing procalcitonin-guidance with standard therapy. Comparative Study; Journal Article; Randomized Controlled Trial; Research Support, Non‐U.S. Gov't. *Chest*. 2007;131(1):9‐19. doi:10.1378/chest.06-1500  890. Stolz D, Christ-Grain M, Bingisser R, et al. Antibiotic treatment of exacerbations of COPD: A randomized, controlled trial comparing procalcitonin-guidance with standard therapy. Article. *Chest*. 2007;131(1):9-19. doi:10.1378/chest.06-1500  891. Stolz D, Louis R, Aerts J, et al. Bacterial airway colonization is not associated with increased procalcitonin in stable COPD. Conference Abstract. *European Respiratory Journal*. 2012;40  892. Stolz D, Tamm M. Authors' reply. Letter. *Swiss Medical Weekly*. 2008;138(9-10):151.  893. Stolz D, Tamm M. Discriminate use of antibiotics for exacerbation of COPD. Review. *Current Opinion in Pulmonary Medicine*. 2009;15(2):126-132. doi:10.1097/MCP.0b013e328324e6d7  894. Sturm T, Leiblein J, Schneider-Lindner V, Kirschning T, Thiel M. Association of Microcirculation, Macrocirculation, and Severity of Illness in Septic Shock: A Prospective Observational Study to Identify Microcirculatory Targets Potentially Suitable for Guidance of Hemodynamic Therapy. Article. *Journal of Intensive Care Medicine*. 2018;33(4):256-266. doi:10.1177/0885066616671689  895. Su L, Feng L, Song Q, et al. Diagnostic value of dynamics serum sCD163, sTREM-1, PCT, and CRP in differentiating sepsis, severity assessment, and prognostic prediction. Article. *Mediators of Inflammation*. 2013;2013doi:10.1155/2013/969875  896. Suarez NM, Bunsow E, Falsey AR, Walsh EE, Mejias A, Ramilo O. Superiority of transcriptional profiling over procalcitonin for distinguishing bacterial from viral lower respiratory tract infections in hospitalized adults. Conference Paper. *Journal of Infectious Diseases*. 2015;212(2):213-222. doi:10.1093/infdis/jiv047  897. Subedi B, Louzon P, Zappas K, Onyia W, DeBoer K. Impact of Pharmacist-Led Procalcitonin-Guided Antibiotic Therapy in Critically Ill Patients With Pneumonia. Article. *Hospital Pharmacy*. 2020;55(3):204-210. doi:10.1177/0018578719836643  898. Suberviola B, Rellan L, Riera J, et al. Role of biomarkers in early infectious complications after lung transplantation. Article. *PLoS ONE*. 2017;12(7)doi:10.1371/journal.pone.0180202  899. Suffredini AF, Masur H, Lynch JP. Update in pulmonary and critical care medicine. Article. *Annals of Internal Medicine*. 2010;152(9):601-608. doi:10.7326/0003-4819-152-9-201005040-00247  900. Sun D, Li X, Guo D, et al. Ct quantitative analysis and its relationship with clinical features for assessing the severity of patients with COVID-19. Article. *Korean Journal of Radiology*. 2020;21(7):859-868. doi:10.3348/kjr.2020.0293  901. Sun WP, Yuan GX, Hu YJ, Liao LZ, Fu L. Effect of low-dose glucocorticoid on corticosteroid insuffi cient patients with acute exacerbation of chronic obstructive pulmonary disease. Journal: Article. *World journal of emergency medicine*. 2015;6(1):34‐39. doi:10.5847/wjem.j.1920-8642.2015.01.006  902. Sun ZZ, Li Y, Shu M. Effect of early airway management on postoperative rehabilitation of patients undergoing cervical spine surgery. Journal: Article. *Indian Journal of Pharmaceutical Sciences*. 2021;83:153‐157. doi:10.36468/pharmaceutical-sciences.spl.268  903. Surme S, Buyukyazgan A, Bayramlar OF, et al. Predictors of intensive care unit admission or mortality in patients with coronavirus disease 2019 pneumonia in istanbul, turkey. Article. *Japanese Journal of Infectious Diseases*. 2021;74(5):458-464. doi:10.7883/yoken.JJID.2020.1065  904. Surmont VF, Salhi B, Haenebalcke C, et al. REINFORCE: A randomized trial of resistance training in patients with radically treated respiratory cancer. Conference Abstract. *Journal of Clinical Oncology*. 2013;31(15)  905. Surmont VF, Salhi B, Haenebalcke C, et al. REINFORCE: a randomized trial of resistance training in patients with radically treated respiratory cancer. Journal: Conference Abstract. *Journal of clinical oncology*. 2013;31(15 SUPPL. 1)  906. Suryananda TD, Yudhawati R. Association of serum KL-6 levels on COVID-19 severity: A cross-sectional study design with purposive sampling. Article. *Annals of Medicine and Surgery*. 2021;69doi:10.1016/j.amsu.2021.102673  907. Sweeney DA, Kalil AC. Choosing the Duration of Therapy for Bacteremia: Keep Calm and Work with Your Infectious Disease and Antibiotic Stewardship Colleagues∗. Editorial. *Critical Care Medicine*. 2016;44(2):439-440. doi:10.1097/CCM.0000000000001503  908. Tabata N, Al-Kassou B, Sugiura A, et al. Predictive factors and long-term prognosis of transcatheter aortic valve implantation-associated endocarditis. Article. *Clinical Research in Cardiology*. 2020;109(9):1165-1176. doi:10.1007/s00392-020-01609-w  909. Tack G, Walker PP, Thompson E, Crone H, Flatt G, Pearson MG. Could the average “total bed days per patient” be an indicator of combined hospital and community care? Conference Abstract. *Thorax*. 2009;64:A66-A67. doi:10.1136/thx.2009.127100b  910. Tack G, Walker PP, Thompson E, Holton K, Pearson M, Hill S. Wide variation in hospital bed days for COPD exacerbations per 1000 local population may be a useful indicator of the local organisation and delivery of care. Conference Abstract. *American Journal of Respiratory and Critical Care Medicine*. 2010;181(1)  911. Tai DBG, Goldstein R. Procalcitonin-guided antibiotic strategy for lower respiratory tract infections: Outcomes and safety of withholding antibiotics. Conference Abstract. *Open Forum Infectious Diseases*. 2019;6:S747. doi:10.1093/ofid/ofz360.1874  912. Tai DYH. Role of biomarkers in sepsis. Conference Abstract. *Critical Care and Shock*. 2012;15(3):60-62.  913. Tajti G, Papp C, Bíró K, et al. Questions in differential diagnosis of bronchial asthma, chronic obstructive pulmonary disease and overlap syndrome. Conference Abstract. *Acta Physiologica*. 2014;211:105. doi:10.1111/apha.12362  914. Takahashi H, Yokozawa T, Oda T. Listeria monocytogenes bacteremia one month after contact with raw venison: A case report. Article. *IDCases*. 2022;28doi:10.1016/j.idcr.2022.e01515  915. Tanaka Y, Yamamoto K, Fukuda Y, et al. An adult case of invasive pneumococcal disease due to serotype 12F-specific polysaccharide antibody failure following a 23-valent polysaccharide vaccination. Letter. *Emerging Microbes and Infections*. 2020;9(1):2266-2268. doi:10.1080/22221751.2020.1830716  916. Tanayan C, Janda T, Ilkanich C, Pastoral M, Silver K. AMIODARONE TOXICITY: SOONER THAN YOU EXPECT. Conference Abstract. *Journal of the American College of Cardiology*. 2019;73(9 Supplement 1):2938. doi:10.1016/S0735-1097(19)33544-2  917. Tang B, Chen M, Lian XZ, et al. Study of the correlation between the acute exacerbation of chronic obstructive pulmonary disease (syndrome of phlegm-heat obstructing lung) and the clinically relevant indicators. Article. *Chinese Journal of Evidence-Based Medicine*. 2016;16(8):876-881. doi:10.7507/1672-2531.20160135  918. Tarquinio N, Falsetti L, Fioranelli A, et al. SOFA-T score is associated with an increased mortality rate among patients affected by severe sepsis and septic shock. Conference Abstract. *Italian Journal of Medicine*. 2015;9:108. doi:10.4081/itjm.2015.s2  919. Taskin G, Selcuk A, Uysal S. Serial C1 inhibitor levels in patients with ventilator-associated pneumonia: A prospective observational study. Article. *Journal of Critical and Intensive Care*. 2021;12(1):8-14. doi:10.37678/dcybd.2020.2538  920. Teixeira A, Legrain S, Ray P. Etiological diagnosis of acute dyspnea in elderly patients: role of biomarkers in emergencies. Short Survey. *Presse Medicale*. 2009;38(10):1506-1515. doi:10.1016/j.lpm.2008.12.028  921. Telli Dizman G, Metan G, Ayaz Ceylan ÇM, et al. A COVID-19 first evaluation clinic at a university hospital in Turkey. Article. *Turkish Journal of Medical Sciences*. 2022;52(1):1-10. doi:10.3906/sag-2104-152  922. Tessmer M. Community-acquired lower respiratory tract infections/community-acquired pneumonias in adult patients - Diagnosis, antimicrobial therapy and management: Recommendations of the current S3 guideline. Conference Paper. *Arzneimitteltherapie*. 2010;28(2):60-67.  923. Teta M, Drabkin MJ. Fatal retroperitoneal hematoma associated with Covid-19 prophylactic anticoagulation protocol. Article. *Radiology Case Reports*. 2021;16(7):1618-1621. doi:10.1016/j.radcr.2021.04.029  924. Tezza F, Lorenzoni G, Azzolina D, Barbar S, Leone LAC, Gregori D. Predicting in-hospital mortality of patients with covid-19 using machine learning techniques. Article. *Journal of Personalized Medicine*. 2021;11(5)doi:10.3390/jpm11050343  925. Thompson P. Respirology: Editorial. Editorial. *Respirology*. 2008;13(4):487. doi:10.1111/j.1440-1843.2008.01305.x  926. Ticona JH, Zaccone VM, McFarlane IM. Community-Acquired Pneumonia: A Focused Review. *American journal of medical case reports*. 2021;9(1):45-52. doi:10.12691/ajmcr-9-1-12  927. Titova EV, Henriksen AH, Steinshamn S, Asberg A. Procalcitonin as a predictor of pneumonia in patients hospitalized with acute exacerbation of COPD: a prospective observational study. Conference Abstract. *European Respiratory Journal*. 2017;50doi:10.1183/1393003.congress-2017.A3609  928. Tokman S, Schuetz P, Bent S. Procalcitonin-guided antibiotic therapy for chronic obstructive pulmonary disease exacerbations. Review. *Expert Review of Anti-Infective Therapy*. 2011;9(6):727-735. doi:10.1586/eri.11.45  929. Tosoni A, Paratore M, Piscitelli P, Addolorato G, De Cosmo S, Mirijello A. The use of procalcitonin for the management of sepsis in Internal Medicine wards: current evidence. *Panminerva Med*. Mar 2020;62(1):54-62. doi:10.23736/s0031-0808.19.03809-6  930. Townsend J, Adams V, Galiatsatos P, et al. Procalcitonin-guided antibiotic therapy reduces antibiotic use for lower respiratory tract infections in a United States medical center: Results of a clinical trial. Article. *Open Forum Infectious Diseases*. 2018;5(12)doi:10.1093/ofid/ofy327  931. Townsend J, Adams V, Galiatsatos P, et al. Procalcitonin-guided antibiotic therapy reduces antibiotic use for lower respiratory tract infections in a United States medical center: results of a clinical trial. Journal: Article. *Open forum infectious diseases*. 2018;5(12)doi:10.1093/ofid/ofy327  932. Townsend J, Adams-Sommer V, Galiatsatos P, et al. Procalcitonin-guided antibiotic therapy for lower respiratory tract infections in a us academic medical center. Conference Abstract. *Open Forum Infectious Diseases*. 2018;5:S570-S571. doi:10.1093/ofid/ofy210.1624  933. Travaglino F, Russo V, De Berardinis B, et al. Thirty and ninety days mortality predictive value of admission and in-hospital procalcitonin and mid-regional pro-adrenomedullin testing in patients with dyspnea. Results from the VERyfing DYspnea trial. Article. *American Journal of Emergency Medicine*. 2014;32(4):334-341. doi:10.1016/j.ajem.2013.12.045  934. Triner M, Patel S, Craft R, Rajkumar A, Patel T. Impact of procalcitonin-guided antibiotic management in chronic obstructive pulmonary disease exacerbation and community-acquired pneumonia. Conference Abstract. *Open Forum Infectious Diseases*. 2019;6:S483-S484. doi:10.1093/ofid/ofz360.1200  935. Tsao TC, Tsai HC, Chang SC. Clinical usefulness of urinary fatty acid binding proteins in assessing the severity and predicting treatment response of pneumonia in critically ill patients: A cross-sectional study. Article. *Medicine (United States)*. 2016;95(19)doi:10.1097/MD.0000000000003682  936. Turkkan S, Beyoglu MA, Sahin MF, Yazicioglu A, Tezer Tekce Y, Yekeler E. COVID-19 in lung transplant recipients: A single-center experience. Article. *Transplant Infectious Disease*. 2021;23(5)doi:10.1111/tid.13700  937. Turner AM, Sen S, Steeley C, et al. Evaluation of oxygen prescription in relation to hospital admission rate in patients with chronic obstructive pulmonary disease. Article. *BMC Pulmonary Medicine*. 2014;14(1)doi:10.1186/1471-2466-14-127  938. Turrini M, Gardellini A, Beretta L, et al. Clinical course and risk factors for in-hospital mortality of 205 patients with SARS-CoV-2 pneumonia in Como, Lombardy region, Italy. Article. *Vaccines*. 2021;9(6)doi:10.3390/vaccines9060640  939. Twomey P, Rogerson S. 'Commissioning in the round - A co-ordinated approach': A proposed model. Article. *Quality in Primary Care*. 2006;14(2):99-105.  940. Vaillant JJ, Mersfelder TL, Maynard RS, Kavanaugh K. Procalcitonin-Guided Antibiotic Discontinuation: An Antimicrobial Stewardship Initiative to Assist Providers. Article. *Journal of Clinical Outcomes Management*. 2020;27(3):111-114.  941. Valenzuela RG, Michelen Y, Bracey A, et al. Outcomes in Hispanics With COVID-19 Are Similar to Those of Caucasian Patients in Suburban New York. Article. *Academic Emergency Medicine*. 2020;27(12):1260-1269. doi:10.1111/acem.14146  942. van de Geijn GM, Denker S, Meuleman-van Waning V, et al. Evaluation of new laboratory tests to discriminate bacterial from nonbacterial chronic obstructive pulmonary disease exacerbations. *Int J Lab Hematol*. Dec 2016;38(6):616-628. doi:10.1111/ijlh.12550  943. Van Der Does Y, Rood PPM, Haagsma JA, Patka P, Van Gorp ECM, Limper M. Procalcitonin-guided therapy for the initiation of antibiotics in the ED: A systematic review. Review. *American Journal of Emergency Medicine*. 2016;34(7):1286-1293. doi:10.1016/j.ajem.2016.03.065  944. van der Eerden MM. Use of antibiotics for asthma and COPD exacerbation. Article. *Nederlands tijdschrift voor geneeskunde*. 2019;163  945. Van Der Maas M, Steuten L. A PCT-algorithm to guide antibiotic therapy in patients hospitalized with COPD exacerbations leads to net cost savings by reducing frequency and duration of antibiotic use as compared to current practice. Conference Abstract. *Value in Health*. 2015;18(7):A356.  946. van der Maas ME, Mantjes G, Steuten LM. Procalcitonin Biomarker Algorithm Reduces Antibiotic Prescriptions, Duration of Therapy, and Costs in Chronic Obstructive Pulmonary Disease: A Comparison in the Netherlands, Germany, and the United Kingdom. *Omics : a journal of integrative biology*. Apr 2017;21(4):232-243. doi:10.1089/omi.2016.0186  947. Van Der Maas ME, Mantjes G, Steuten LMG. Procalcitonin Biomarker Algorithm Reduces Antibiotic Prescriptions, Duration of Therapy, and Costs in Chronic Obstructive Pulmonary Disease: A Comparison in the Netherlands, Germany, and the United Kingdom. Article. *OMICS A Journal of Integrative Biology*. 2017;21(4):232-243. doi:10.1089/omi.2016.0186  948. Van Der Merwe C. Prescribing of acute antibiotics for common infections: Audit assessing adherence to current recommendations. Conference Abstract. *International Journal of Pharmacy Practice*. 2013;21:132-133. doi:10.1111/ijpp.12064  949. Van Meerbeeck J, Salhi B, Haenebalcke C, et al. Resistance training in patients with radically treated respiratory cancer: A prospective randomized multi-center study (REINFORCE). Conference Abstract. *European Journal of Cancer*. 2013;49:S796-S797. doi:10.1016/S0959-8049(13)70065-0  950. van Velzen P, Riet GT, Brinkman P, Sterk PJ, Prins JM. Doxycycline for exacerbations of chronic obstructive pulmonary disease in outpatients: Who benefits? Letter. *ERJ Open Research*. 2020;6(2):1-4. doi:10.1183/23120541.00099-2020  951. Vandewoude MFJ. Infections in older people. Conference Paper. *Aging Health*. 2007;3(6):711-714. doi:10.2217/1745509X.3.6.711  952. Vasilakopoulou A, Vourli S, Siafakas N, Kavatha D, Tziolos N, Pournaras S. Enterococcus casseliflavus Bacteraemia in a Patient with Chronic Renal Disease. Article. *Infectious Disease Reports*. 2020;12(3):70-73. doi:10.3390/IDR12030015  953. Verduri A, D'Amico R, Ruggieri V, Vicini R, Liverani A, Plebani M. Pro-Calcitonin Guided Antibiotic Treatment Of Acute Exacerbations Of Chronic Obstructive Pulmonary Disease. *American journal of respiratory and critical care medicine*. 2012;185(Meeting Abstracts):A3026.  954. Verduri A, D'Amico R, Ruggieri V, et al. Pro-calcitonin guided antibiotic treatment of acute exacerbations of chronic obstructive pulmonary disease. Journal: Conference Abstract. *American journal of respiratory and critical care medicine*. 2012;185  955. Verduri A, D'Amico R, Ruggieri V, et al. Pro-calcitonin guided antibiotic treatment of acute exacerbations of chronic obstructive pulmonary disease. Conference Abstract. *American Journal of Respiratory and Critical Care Medicine*. 2012;185  956. Verduri A, Luppi F, D'Amico R, et al. Antibiotic treatment of severe exacerbations of chronic obstructive pulmonary disease with procalcitonin: a randomized noninferiority trial. *PLoS One*. 2015;10(3):e0118241. doi:10.1371/journal.pone.0118241  957. Verduri A, Luppi F, D'Amico R, et al. Antibiotic treatment of severe exacerbations of chronic obstructive pulmonary disease with procalcitonin: a randomized noninferiority trial. Journal Article; Multicenter Study; Randomized Controlled Trial; Research Support, Non‐U.S. Gov't. *PloS one*. 2015;10(3):e0118241. doi:10.1371/journal.pone.0118241  958. Verduri A, Luppi F, D'Amico R, et al. Antibiotic treatment of severe exacerbations of chronic obstructive pulmonary disease with procalcitonin: A randomized noninferiority trial. Article. *PLoS ONE*. 2015;10(3)doi:10.1371/journal.pone.0118241  959. Viasus D, Simonetti AF, Estupiñan-Bohórquez AF, Carratalà J. Effects of age and comorbidities on serum levels of inflammatory markers in community-acquired pneumonia. Article. *European Journal of Clinical Investigation*. 2021;51(6)doi:10.1111/eci.13480  960. Villalba GC, Amat-Santos IJ, Dueñas C, et al. Impact of the presence of heart disease, cardiovascular medications and cardiac events on outcome in covid-19. Article. *Cardiology Journal*. 2021;28(3):360-368. doi:10.5603/CJ.a2021.0034  961. Voshaar T, Stais P, Köhler D, Dellweg D. Conservative management of covid-19 associated hypoxaemia. Article. *ERJ Open Research*. 2021;7(1)doi:10.1183/23120541.00026-2021  962. Voutsas V, Soulountsi V, Kontou P, et al. Prognostic value of procalcitonin and b-type natriuretic peptide (BNP) in ICU patients with new onset of fever. Conference Abstract. *Intensive Care Medicine*. 2010;36:S135. doi:10.1007/s00134-010-1999-x  963. Wade RC, Wells JM. Rebuttal From Drs Wade and Wells. Editorial. *Chest*. 2020;157(5):1078-1079. doi:10.1016/j.chest.2019.11.052  964. Walker PP, Thompson E, Hill SL, Holton K, Bodger K, Pearson MG. Commissioning for COPD care: a new, recordable metric that supports the patient interest. Article. *Journal of public health (Oxford, England)*. 2016;38(2):396-402. doi:10.1093/pubmed/fdv056  965. Walsh EE, Swinburne AJ, Becker KL, et al. Can serum procalcitonin levels help interpret indeterminate chest radiographs in patients hospitalized with acute respiratory illness? Article. *Journal of Hospital Medicine*. 2013;8(2):61-67. doi:10.1002/jhm.1984  966. Wan R, Bai L, Yan Y, et al. A Clinically Applicable Nomogram for Predicting the Risk of Invasive Mechanical Ventilation in Pneumocystis jirovecii Pneumonia. Article. *Frontiers in cellular and infection microbiology*. 2022;12doi:10.3389/fcimb.2022.850741  967. Wang F, Qu M, Zhou X, et al. The timeline and risk factors of clinical progression of COVID-19 in Shenzhen, China. Article. *Journal of Translational Medicine*. 2020;18(1)doi:10.1186/s12967-020-02423-8  968. Wang F, Ran L, Qian C, et al. Epidemiology and Outcomes of Acute Kidney Injury in COVID-19 Patients with Acute Respiratory Distress Syndrome: A Multicenter Retrospective Study. Article. *Blood Purification*. 2021;50(4-5):499-505. doi:10.1159/000512371  969. Wang G, Zhang Q, Wu C, et al. Clinical Characteristics of Adult Fevered COVID-19 Patients and Predictors for Developing Severe Events. Article. *Frontiers in medicine*. 2020;7doi:10.3389/fmed.2020.00324  970. Wang H, Qu G. Observation of the Effect of Singulair Combined With Ketotifen in the Treatment of Acute Exacerbation of Chronic Obstructive Pulmonary Disease With Airway Hyperresponsiveness and Its Influence on Th17/Treg. *Frontiers in surgery*. 2022;9:848724. doi:10.3389/fsurg.2022.848724  971. Wang HC, Chang K, Lu PL, Tsai KB, Chen HC. Fatal invasive aspergillosis: a rare co-infection with an unexpected image presentation in a patient with dengue shock syndrome. Article. *Clinical Respiratory Journal*. 2017;11(2):248-253. doi:10.1111/crj.12323  972. Wang HQ, Wu FJ, Cao DK, Niu YY, Yao YW. Clinical efficacy of vitamin D combined with budesonide and formoterol fumarate powder for inhalation and azithromycin in sequential treatment of chronic obstructive pulmonary disease patients with infectious pneumonia. *Chinese journal of nosocomiology*. 2019;29:535‐539.  973. Wang J, Cao B. Procalcitonin and other markers to guide antibiotic use in chronic obstructive pulmonary disease exacerbations in the era of antimicrobial resistance. Review. *Current Opinion in Pulmonary Medicine*. 2019;25(2):158-164. doi:10.1097/MCP.0000000000000555  974. Wang J, Gao Y, Zhu J, Huang Y, Li W. Serum procalcitonin levels in predicting the prognosis of severe pneumonia patients and its correlation with white blood cell count and c-reactive protein levels. Article. *International Journal of Clinical and Experimental Medicine*. 2020;13(2):809-815.  975. Wang J, Zhang H, Qiao R, et al. Thrombo-inflammatory features predicting mortality in patients with COVID-19: The FAD-85 score. Article. *Journal of International Medical Research*. 2020;48(9)doi:10.1177/0300060520955037  976. Wang M, Jiang N, Li C, et al. Sex-Disaggregated Data on Clinical Characteristics and Outcomes of Hospitalized Patients With COVID-19: A Retrospective Study. Article. *Frontiers in cellular and infection microbiology*. 2021;11doi:10.3389/fcimb.2021.680422  977. Wang Y, Chen Y, Zhou X. Tocilizumab, an Exploratory Treatment for Severe COVID-19 Patients. Article. *Canadian Journal of Infectious Diseases and Medical Microbiology*. 2022;2022doi:10.1155/2022/6375870  978. Wang Y, Fu X, Yu B, Ai F. Long non-coding RNA THRIL predicts increased acute respiratory distress syndrome risk and positively correlates with disease severity, inflammation, and mortality in sepsis patients. Article. *Journal of Clinical Laboratory Analysis*. 2019;33(6)doi:10.1002/jcla.22882  979. Wang Y, Li J, Li H, Lei P, Shen G, Yang C. Persistence of SARS-CoV-2-specific antibodies in COVID-19 patients. Article. *International Immunopharmacology*. 2021;90doi:10.1016/j.intimp.2020.107271  980. Wang Y, Zheng Y, Zhai YL, Liu FQ, Ding N. Comparative analysis of MCP-1 and TF in elderly patients with acute exacerbations of COPD and its clinical significance. Article. *European Review for Medical and Pharmacological Sciences*. 2015;19(2):215-219.  981. Wang Z, Wang Z. Identification of risk factors for in-hospital death of COVID - 19 pneumonia -- lessions from the early outbreak. Article. *BMC Infectious Diseases*. 2021;21(1)doi:10.1186/s12879-021-05814-4  982. Wang Z, Yang J, Tang X, Pi X, Li L, Yu J. Is blood culture a reliable guide to diagnosing catheter-related bacteremia (CRB)? A case of CRB caused by fungi and seven types of bacteria in a hemodialysis patient. Letter. *International Urology and Nephrology*. 2014;46(12):2421-2422. doi:10.1007/s11255-014-0760-8  983. Ward S, Barnes H, Ward R. Evaluating a respiratory intermediate care team. Article. *Nursing standard (Royal College of Nursing (Great Britain) : 1987)*. 2005;20(5):46-50.  984. Wasielewski S. Procalcitonin test reduces antibiotic use in lower respiratory tract infections. Journal: Short Survey. *Deutsche apotheker zeitung*. 2004;144(30):46‐48.  985. Wasserman E, Perosevic N, Pawar R, Manzoor K, Singha M. NONCARDIOGENIC PULMONARY EDEMA IN AN OBESE PATIENT DUE TO EXCESSIVE DYNAMIC AIRWAY COLLAPSE AND SEVERE OSA. Conference Abstract. *Chest*. 2021;160(4):A2405. doi:10.1016/j.chest.2021.07.2082  986. Wästfelt M, Cao Y, Ström JO. Predictors of post-stroke fever and infections: A systematic review and meta-analysis. Article. *BMC Neurology*. 2018;18(1)doi:10.1186/s12883-018-1046-z  987. Watson RS. Editorial: Is it time to put procalcitonin to the (randomized, controlled) test? Editorial. *Pediatric Critical Care Medicine*. 2008;9(4):446-447. doi:10.1097/PCC.0b013e318172ebfe  988. Wedzicha W. Systemic effects of COPD exacerbations. Conference Abstract. *Clinical Respiratory Journal*. 2011;5:2-3. doi:10.1111/j.1752-699X.2011.00265_3.x  989. Weerasuriya N, Jayawardena M, Snape J. Thoracic aortic dissection mistakenly diagnosed as pulmonary embolism: A potentially fatal error. Article. *CPD Journal Acute Medicine*. 2004;3(3):114-116.  990. Wei Y, Zeng W, Huang X, et al. Clinical characteristics of 276 hospitalized patients with coronavirus disease 2019 in Zengdu District, Hubei Province: A single-center descriptive study. Article. *BMC Infectious Diseases*. 2020;20(1)doi:10.1186/s12879-020-05252-8  991. Weishuai B, Wei C, Xuyun G, et al. Analysis of related factors of carbapenem resistant klebsiella pneumoniae infection in patients with artificial airway. Article. *Zhonghua Wei Zhong Bing Ji Jiu Yi Xue*. 2020;32(11):1324-1330. doi:10.3760/cma.j.cn121430-20200601-00431  992. Weiss TJ, Barranco-Trabi JJ, Brown A, Oommen TT, Mank V, Ryan C. Case Report: Shewanella Algae Pneumonia and Bacteremia in an Elderly Male Living at a Long-Term Care Facility. Article. *American Journal of Tropical Medicine and Hygiene*. 2022;106(1):60-61. doi:10.4269/ajtmh.21-0614  993. Welte T. Treatment of community acquired pneumonia. Review. *Internist*. 2007;48(5):476-488. doi:10.1007/s00108-007-1852-7  994. Welte T. Risk factors and severity scores in hospitalized patients with community-acquired pneumonia: Prediction of severity and mortality. Review. *European Journal of Clinical Microbiology and Infectious Diseases*. 2012;31(1):33-47. doi:10.1007/s10096-011-1272-4  995. Wettersten N, Maisel AS. Biomarkers for Heart Failure: An Update for Practitioners of Internal Medicine. Review. *American Journal of Medicine*. 2016;129(6):560-567. doi:10.1016/j.amjmed.2016.01.013  996. Whisenant JG, Baena J, Cortellini A, et al. A Definitive Prognostication System for Patients With Thoracic Malignancies Diagnosed With Coronavirus Disease 2019: An Update From the TERAVOLT Registry. Article. *Journal of Thoracic Oncology*. 2022;17(5):661-674. doi:10.1016/j.jtho.2021.12.015  997. Williams N, Ostridge K, Kim V, et al. The role of the blood neutrophil-lymphocyte ratio in acute exacerbations of COPD. Conference Abstract. *American Journal of Respiratory and Critical Care Medicine*. 2015;191  998. Wirtz TH, Buendgens L, Weiskirchen R, et al. Association of serum calprotectin concentrations with mortality in critically ill and septic patients. Article. *Diagnostics*. 2020;10(11)doi:10.3390/diagnostics10110990  999. Wolfisberg S, Gregoriano C, Schuetz P. Procalcitonin for individualizing antibiotic treatment: an update with a focus on COVID-19. Review. *Critical Reviews in Clinical Laboratory Sciences*. 2022;59(1):54-65. doi:10.1080/10408363.2021.1975637  1000. Wong HR, Walley KR, Pettilä V, et al. Comparing the prognostic performance of ASSIST to interleukin-6 and procalcitonin in patients with severe sepsis or septic shock. Article. *Biomarkers*. 2015;20(2):132-135. doi:10.3109/1354750X.2014.1000971  1001. Woo MS, Steins D, Häußler V, et al. Control of SARS-CoV-2 infection in rituximab-treated neuroimmunological patients. Letter. *Journal of Neurology*. 2021;268(1):5-7. doi:10.1007/s00415-020-10046-8  1002. Woodhead M, Blasi F, Ewig S, et al. Guidelines for the management of adult lower respiratory tract infections - Full version. Article. *Clinical Microbiology and Infection*. 2011;17(SUPPL. 6):E1-E59. doi:10.1111/j.1469-0691.2011.03672.x  1003. Woodhead MA, Torres A, Ewig S. Pigs might fly. Editorial. *European Respiratory Journal*. 2009;33(6):1241-1243. doi:10.1183/09031936.00075009  1004. Woodruff PG. Novel outcomes and end points: Biomarkers in chronic obstructive pulmonary disease clinical trials. Conference Paper. *Proceedings of the American Thoracic Society*. 2011;8(4):350-355. doi:10.1513/pats.201101-015RM  1005. Wu F, Chipman A, Kozar RA. What's New in Shock, September 2019? Note. *Shock*. 2019;52(3):285-287. doi:10.1097/SHK.0000000000001391  1006. Wu F, Zhou Y, Wang Z, et al. Clinical characteristics of COVID-19 infection in chronic obstructive pulmonary disease: A multicenter, retrospective, observational study. Article. *Journal of Thoracic Disease*. 2020;12(5):1811-1823. doi:10.21037/jtd-20-1914  1007. Wu S, Alikhil M, Forsyth R, Allen B. Impact of Potentially Unwarranted Intravenous Antibiotics Targeting Pulmonary Infections in Acute Decompensated Heart Failure. Article. *Journal of Pharmacy Technology*. 2021;37(6):298-303. doi:10.1177/87551225211038020  1008. Wu W, Liu M, Geng J, Wang M. Teicoplanin combined with conventional vancomycin therapy for the treatment of pulmonary methicillin-resistant Staphylococcus aureus and Staphylococcus epidermidis infections. Journal: Article. *World Journal of Clinical Cases*. 2021;9(34):10549‐10556. doi:10.12998/wjcc.v9.i34.10549  1009. Wu Y, Huang X, Sun J, et al. Clinical characteristics and immune injury mechanisms in 71 patients with COVID-19. Article. *mSphere*. 2020;5(4)doi:10.1128/mSphere.00362-20  1010. Wu Y, Luo Z, Liu C. Variations in fecal microbial profiles of acute exacerbations and stable chronic obstructive pulmonary disease. Article. *Life sciences*. 2021;265doi:10.1016/j.lfs.2020.118738  1011. Wunderink RG. Other Community Respiratory Viruses. Review. *Clinics in Chest Medicine*. 2017;38(1):37-43. doi:10.1016/j.ccm.2016.11.003  1012. Xia RY, Hu XY, Fei YT, et al. Shufeng jiedu capsules for treating acute exacerbations of chronic obstructive pulmonary disease: A systematic review and meta-analysis. Article. *BMC Complementary Medicine and Therapies*. 2020;20(1)doi:10.1186/s12906-020-02924-5  1013. Xiao H, Zhang P, Xiao Y, et al. Diagnostic accuracy of procalcitonin as an early predictor of infection after radical gastrectomy for gastric cancer: A prospective bicenter cohort study. Article. *International Journal of Surgery*. 2020;75:3-10. doi:10.1016/j.ijsu.2020.01.019  1014. Xie C, Wen Y, Zhao Y, et al. Clinical features of patients with bronchiectasis with comorbid chronic obstructive pulmonary disease in China. Article. *Medical Science Monitor*. 2019;25:6805-6811. doi:10.12659/MSM.917034  1015. Xie Y, You Q, Wu C, et al. Impact of cardiovascular disease on clinical characteristics and outcomes of coronavirus disease 2019 (COVID-19). Article. *Circulation Journal*. 2020;84(8):1277-1283. doi:10.1253/circj.CJ-20-0348  1016. Xie Z, Wu W, Meng D, et al. A case of Phaeohyphomycosis caused by Corynespora cassiicola infection. Article. *BMC Infectious Diseases*. 2018;18(1)doi:10.1186/s12879-018-3342-z  1017. Xiong B, Du H, Zhan Z, et al. Effectiveness of adjunctive corticosteroid therapy in patients with severe COVID-19: A retrospective cohort study. Article. *World Journal of Clinical Cases*. 2021;9(15):3546-3558. doi:10.12998/wjcc.v9.i15.3546  1018. Xu G, Yang Y, Du Y, et al. Clinical Pathway for Early Diagnosis of COVID-19: Updates from Experience to Evidence-Based Practice. Review. *Clinical Reviews in Allergy and Immunology*. 2020;59(1):89-100. doi:10.1007/s12016-020-08792-8  1019. Xu L, Mao Y, Chen G. Risk factors for 2019 novel coronavirus disease (COVID-19) patients progressing to critical illness: a systematic review and meta-analysis. *Aging (Albany NY)*. Jun 23 2020;12(12):12410-12421. doi:10.18632/aging.103383  1020. Xu W, Huang CL, Fei L, et al. A Novel Prediction Model of COVID-19 Progression: A Retrospective Cohort Study. Article. *Infectious Diseases and Therapy*. 2021;10(3):1491-1504. doi:10.1007/s40121-021-00460-4  1021. Xu Z, Zhang Y, Zhang C, Xiong F, Zhang J, Xiong J. Clinical Features and Outcomes of COVID-19 Patients with Acute Kidney Injury and Acute Kidney Injury on Chronic Kidney Disease. *Aging Dis*. Jun 2022;13(3):884-898. doi:10.14336/ad.2021.1125  1022. Yang L, Jin J, Luo W, Gan Y, Chen B, Li W. Risk factors for predicting mortality of COVID-19 patients: A systematic review and meta-analysis. Review. *PLoS ONE*. 2020;15(11 November 2020)doi:10.1371/journal.pone.0243124  1023. Yang L, Liu T, Liu BC, Liu CT. Severe Pneumonia Advanced to Lung Abscess and Empyema Due to Rothia Mucilaginosa in an Immunocompetent Patient. Letter. *American Journal of the Medical Sciences*. 2020;359(1):54-56. doi:10.1016/j.amjms.2019.10.015  1024. Yang M, Liu X, Hu Q, et al. Eosinopenia as a biomarker for antibiotic use in COPD exacerbations: Protocol for a retrospective hospital-based cohort study. Article. *BMJ Open*. 2022;12(1)doi:10.1136/bmjopen-2021-051939  1025. Yang Q, Liu W, Yu J, Jiang J, Xu T, Zhou Y. Effect of prealbumin level on mortality in heatstroke patients. Article. *Experimental and Therapeutic Medicine*. 2019;17(4):3053-3060. doi:10.3892/etm.2019.7298  1026. Yang Z, Cui M, Zhang X, et al. Identification of Symptom Clusters and Their Influencing Factors in Subgroups of Chinese Patients With Acute Exacerbation of Chronic Obstructive Pulmonary Disease. Article. *Journal of Pain and Symptom Management*. 2020;60(3):559-567. doi:10.1016/j.jpainsymman.2020.03.037  1027. Yao Q, Wang P, Wang X, et al. A retrospective study of risk factors for severe acute respiratory syndrome coronavirus 2 infections in hospitalized adult patients. Article. *Polish Archives of Internal Medicine*. 2020;130(5):390-399. doi:10.20452/pamw.15312  1028. Yaqub S, Hamid A, Naeem Z. COVID-19 in Hemodialysis Patients. Letter. *Journal of the College of Physicians and Surgeons Pakistan*. 2021;31(7):S141. doi:10.29271/jcpsp.2021.Supp2.S141  1029. Ye Y, Wu X, Li X, et al. Prediction and follow-up of risk factors for severe SARS-CoV-2 pneumonia and application of CT visual scoring. Conference Paper. *Technology and Health Care*. 2021;29(S1):S153-S164. doi:10.3233/THC-218016  1030. Yealy DM, Fine MJ. Measurement of serum procalcitonin: A step closer to tailored care for respiratory infections? Editorial. *JAMA - Journal of the American Medical Association*. 2009;302(10):1115-1116. doi:10.1001/jama.2009.1318  1031. Yeşilbaş O, Şevketoğlu E, Kıhtır HS, et al. [A case of bronchiolitis obliterans secondary to human metapneumovirus bronchiolitis]. *Mikrobiyoloji bulteni*. Oct 2016;50(4):606-612. İnsan metapnömovirus bronşiyolitine ikincil gelişen bronşiyolitis obliterans olgusu. doi:10.5578/mb.32195  1032. Yeşildaǧ M, Bakdik BÖ, Şentürk Z, Bekçi TT. Clinical characteristics of hospitalized patients with viral pneumonia diseases, September 2017-April 2018. Conference Abstract. *Turkish Thoracic Journal*. 2019;20:S175. doi:10.5152/TurkThoracJ.2019.175  1033. Yew MS, Abisheganaden JA, Xu HY. Predictors of elevated follow-up high sensitivity troponin after an initial normal value during acute exacerbations of chronic obstructive pulmonary disease. Conference Abstract. *European Heart Journal*. 2022;43(SUPPL 1):i102. doi:10.1093/eurheartj/ehab849.084  1034. Yifan C, Jun P. Understanding the Clinical Features of Coronavirus Disease 2019 From the Perspective of Aging: A Systematic Review and Meta-Analysis. Review. *Frontiers in Endocrinology*. 2020;11doi:10.3389/fendo.2020.557333  1035. Yilmaz Demırcı N, Köktürk N. Spontaneous pneumopericardium in a patient with COPD. Letter. *Tuberkuloz ve Toraks*. 2016;64(3):260-262. doi:10.5578/tt.24224  1036. Yormaz B, Findik D, Süerdem M. Differences of viral panel positive versus negative by real-time PCR in COPD exacerbated patients. Article. *Tuberkuloz ve Toraks*. 2019;67(2):124-130. doi:10.5578/tt.68471  1037. Yormaz B, Fındık D, Süerdem M. Differences of viral panel positive versus negative by real-time PCR in COPD exacerbated patients. *Tuberk Toraks*. Jun 2019;67(2):124-130. KOAH alevlenmeli hastalarda gerçek zamanlı PCR tekniğiyle viral panel pozitif olanların negatif olanlardan farklılığı. doi:10.5578/tt.68471  1038. Younis I, Roberts NJ, Partridge MR. Barriers to healthcare professionals encouraging self-management in asthma and COPD. Conference Abstract. *Thorax*. 2009;64:A93. doi:10.1136/thx.2009.127134r  1039. Younis I, Roberts NJ, Partridge MR. Self-management in asthma and COPD. Conference Abstract. *Thorax*. 2009;64:A165. doi:10.1136/thx.2009.127191x  1040. Yousuf A, McAuley H, Elneima O, Brightling CE. The different phenotypes of COPD. Article. *British medical bulletin*. 2021;137(1):82-97. doi:10.1093/bmb/ldaa043  1041. Yuan SC, Huang XL, Hua SY, Zhou YH, Rui QL. Effect of acupuncture on diaphragmatic function in patients with AECOPD typeⅡ respiratory failure evaluated by ultrasound detection. Journal Article; Randomized Controlled Trial. *Zhongguo zhen jiu [Chinese acupuncture & moxibustion]*. 2021;41(7):703‐710. doi:10.13703/j.0255-2930.20200606-k0007  1042. Yue-liang X, Jiang-lin W, Hui-qin Y, et al. The risk factors for severe patients with COVID-19 in China: A systematic review and meta-analysis. Article. *European Journal of Inflammation*. 2021;19doi:10.1177/20587392211000890  1043. Zahra A, Fries BC, Sae-Tia S. Characteristics and Outcomes in Hospitalized Patients with Covid-19 Complicated by Fungemia: A Single Center Retrospective Study. Conference Abstract. *Open Forum Infectious Diseases*. 2021;8(SUPPL 1):S278-S279. doi:10.1093/ofid/ofab466.549  1044. Zaini J, Fadhillah MR, Reisa T, Isbaniah F, Handayani RRD. Tuberculosis and COVID-19 coinfection: A report of two cases at a tertiary referral in Indonesia. Article. *Journal of Infection in Developing Countries*. 2022;16(3):478-483. doi:10.3855/jidc.15481  1045. Zaldua JC, Lawrence M, Whitley J, et al. Clot microstructure ( df) as a biomarker and measurement of thrombogenicity in acute exacerbation of chronic obstructive pulmonary disease (AECOPD) S Pillai1. Conference Abstract. *Critical Care*. 2022;26(SUPPL 1)doi:10.1186/s13054-022-03927-z  1046. Zarogoulidis P, Hohenforst-Schmidt W, Huang H, et al. Vapor for lung volume reduction; pros and cons. Article. *Expert Review of Respiratory Medicine*. 2020;14(12):1189-1195. doi:10.1080/17476348.2020.1816467  1047. Zarogoulidis P, Kouliatsis G, Papanas N, et al. Long-term respiratory follow-up of H1N1 infection. Article. *Virology Journal*. 2011;8doi:10.1186/1743-422X-8-319  1048. Żegleń S, Karolak W, Mikołajczyk G, et al. Cryobiopsy as a New Tool for Complications Diagnosis During Follow-up After Lung Transplantation: Single Institution Case Series. Article. *Transplantation Proceedings*. 2021;53(6):2008-2012. doi:10.1016/j.transproceed.2021.03.041  1049. Zeng H, Wang D, Nie J, et al. The efficacy assessment of convalescent plasma therapy for COVID-19 patients: a multi-center case series. Article. *Signal Transduction and Targeted Therapy*. 2020;5(1)doi:10.1038/s41392-020-00329-x  1050. Zeng XB, Deng JH, Gui JP, He D, Li N. Clinical research on procalcitonin guidance on the use and termination of antibiotic treatment in acute exacerbation of chronic obstructive pulmonary disease. *Progress in modern biomedicine*. 2017;17:3310‐3312.  1051. Zhai H, Luo S, Lin L, Du D, Duan B. [Clinical study on optimal switching mode in sequential noninvasive-invasive mechanical ventilation for acute exacerbation of chronic obstructive pulmonary disease]. *Zhonghua Wei Zhong Bing Ji Jiu Yi Xue*. Feb 2020;32(2):161-165. doi:10.3760/cma.j.cn121430-20190923-00030  1052. Zhan Y, Ye F. Early diagnosis of invasive pulmonary aspergillus in patients with chronic obstructive pulmonary diseases: A case control study. Conference Abstract. *European Respiratory Journal*. 2019;54doi:10.1183/13993003.congress-2019.PA2897  1053. Zhang BY, Sun YT, Pan H, Zhang L. Clinical characteristics of nutritional indicators in patients with Covid-19. Article. *Indian Journal of Pharmaceutical Sciences*. 2021;83:57-61. doi:10.36468/pharmaceutical-sciences.spl.251  1054. Zhang C, Ren D, Ouyang C, Wang Q, Liu L, Zou H. Effect of standardized enteral nutrition on AECOPD patients with respiratory failure. Journal: Article. *American journal of translational research*. 2021;13(9):10793‐10800.  1055. Zhang G, Hu C, Luo L, et al. Clinical features and short-term outcomes of 221 patients with COVID-19 in Wuhan, China. Article. *Journal of Clinical Virology*. 2020;127doi:10.1016/j.jcv.2020.104364  1056. Zhang H, He H, Chen Y. THE CLINICAL VALUE OF NONINVASIVE POSITIVE PRESSURE VENTILATION IN COPD WITH ACUTE RESPIRATORY FAILURE. Article. *Acta Medica Mediterranea*. 2022;38(3):1733-1737. doi:10.19193/0393-6384_2022_3_264  1057. Zhang H, Zhong F, Wang B, Liao M. A nomogram predicting the severity of COVID-19 based on initial clinical and radiologic characteristics. Article. *Future Virology*. 2022;17(4):221-229. doi:10.2217/fvl-2020-0193  1058. Zhang J. Single center experience of ROSE+BALF+TBLB+NGS in diagnose of pneumocystis pneumonia in lung transplant recipients. Conference Abstract. *American Journal of Respiratory and Critical Care Medicine*. 2019;199(9)  1059. Zhang JJ, Dong X, Cao YY, et al. Clinical characteristics of 140 patients infected with SARS-CoV-2 in Wuhan, China. Article in Press. *Allergy: European Journal of Allergy and Clinical Immunology*. 2020;doi:10.1111/all.14238  1060. Zhang L, Huang J, Xu T, Lin Y. [Procalcitonin-guided algorithms of antibiotic therapy in community-acquired lower respiratory tract infections: a systematic review and meta-analysis of randomized controlled trials]. Review. *Zhonghua jie he he hu xi za zhi = Zhonghua jiehe he huxi zazhi = Chinese journal of tuberculosis and respiratory diseases*. 2012;35(4):275-282.  1061. Zhang P, Yan H, Wang S, et al. Post-radiotherapy maintenance treatment with fluticasone propionate and salmeterol for lung cancer patients with grade III radiation pneumonitis: A case report. Article. *Medicine (United States)*. 2018;97(21)doi:10.1097/MD.0000000000010681  1062. Zhang Q, Li CS. Risk stratification and prognostic evaluation of endothelial cell-specific molecule1, von willebrand factor, and a disintegrin-like and metalloprotease with thrombospondin type 1 motif for sepsis in the emergency department: An observational study. Article. *Experimental and Therapeutic Medicine*. 2019;17(6):4527-4535. doi:10.3892/etm.2019.7485  1063. Zhang T, Huang WS, Guan W, et al. Risk factors and predictors associated with the severity of COVID-19 in China: A systematic review, meta-analysis, and meta-regression. Review. *Journal of Thoracic Disease*. 2020;12(12):7429-7441. doi:10.21037/jtd-20-1743  1064. Zhang XQ, Lei Y, Tan XL, et al. Optimization of Early Antimicrobial Strategies for Lung Transplant Recipients Based on Metagenomic Next-Generation Sequencing. Article. *Frontiers in Microbiology*. 2022;13doi:10.3389/fmicb.2022.839698  1065. Zhang XX, Yang Y, Zhao W, Cui LY, Wang YQ, Nan YM. [Analysis of risk factors and prognosis of cirrhosis combined with bacterial pneumonia]. *Zhonghua Gan Zang Bing Za Zhi*. Jul 20 2020;28(7):561-566. doi:10.3760/cma.j.cn501113-201200624-00344  1066. Zhang Y, Zhou L. Diagnostic value of C-reactive protein and procalcitonin for bacterial infection in acute exacerbations of chronic obstructive pulmonary disease. Article. *Zhong nan da xue xue bao Yi xue ban = Journal of Central South University Medical sciences*. 2014;39(9):939-943. doi:10.11817/j.issn.1672-7347.2014.09.013  1067. Zhang Z, Wu X. Clinical characteristics and risks of china's 2019 novel coronavirus patients with Aki: A systematic review and meta-analysis. Conference Abstract. *Nephrology*. 2020;25(SUPPL 1):5. doi:10.1111/nep.13771  1068. Zhang Z, Zhang L, Zha D, Hu C, Wu X. Clinical characteristics and risks of Chinàs 2019 novel coronavirus patients with AKI: a systematic review and meta-analysis. Letter. *Renal Failure*. 2020;42(1):926-931. doi:10.1080/0886022X.2020.1812401  1069. Zhao H, Gu H, Liu T, Ge J, Shi G. Analysis of curative effect of adjuvant therapy with bronchoalveolar lavage on COPD patients complicated with pneumonia. Journal Article. *Experimental and therapeutic medicine*. 2018;16(5):3799‐3804. doi:10.3892/etm.2018.6662  1070. Zhao X, Chen YX, Li CS. The prognostic performance of the complement system in septic patients in emergency department: A cohort study. Article. *Biomarkers in Medicine*. 2015;9(7):661-668. doi:10.2217/bmm.15.32  1071. Zhao Y, Li F, Liu Y, et al. Comparison of efficiency of inhaled and intravenous corticosteroid on pregnant women with COPD and the effects on the expression of PCT and hs-CRP. Journal Article. *Experimental and therapeutic medicine*. 2018;15(6):4717‐4722. doi:10.3892/etm.2018.6011  1072. Zhao YF, Jiang YP, Zhou LF, Wu XL. The value of assessment tests in patients with acute exacerbation of chronic obstructive pulmonary disease. Article. *American Journal of the Medical Sciences*. 2014;347(5):393-399. doi:10.1097/MAJ.0b013e31829a63b1  1073. Zhao Z, Chen A, Hou W, et al. Prediction model and risk scores of ICU admission and mortality in COVID-19. Article. *PLoS ONE*. 2020;15(7 July)doi:10.1371/journal.pone.0236618  1074. Zhong J, Li X, Zhang W, Chen D, Zhang LY, Li S. The Role of Soluble Programmed Death Protein 1 in Immunosuppression of Sepsis and its Estimation Value in Prognosis. Article. *Indian Journal of Pharmaceutical Sciences*. 2021;83:45-54. doi:10.36468/pharmaceutical-sciences.spl.331  1075. Zhong L, Wu Y, Gao J, et al. Effects of hypertension on the outcomes of COVID-19: a multicentre retrospective cohort study. Article. *Annals of Medicine*. 2021;53(1):770-776. doi:10.1080/07853890.2021.1931957  1076. Zhong LJ, Zhang LS, Gu CF, et al. Procalcitonin impact analysis, respiratory function and blood gas analysis on Xiaoqinglong decoction combined with non-invasive ventilation in treatment of AECOPD patients. Journal Article; Randomized Controlled Trial. *Zhongguo Zhong yao za zhi [China journal of Chinese materia medica]*. 2018;43(14):3026‐3030. doi:10.19540/j.cnki.cjcmm.20180327.002  1077. Zhou A, Zhou Z, Zhao Y, Chen P. The recent advances of phenotypes in acute exacerbations of COPD. Review. *International Journal of COPD*. 2017;12:1009-1018. doi:10.2147/COPD.S128604  1078. Zhou F, Gu L, Qu JX, Liu YM, Cao B. Evaluating the utility of Binax NOW Streptococcus pneumoniae urinary antigen test in adults with community acquired pneumonia in China. Article. *Clinical Respiratory Journal*. 2018;12(2):425-432. doi:10.1111/crj.12533  1079. Zhou J, Chen B, Wang Y, Wu F. Epidemiological and clinical characteristics of 217 cases of COVID-19 in Jiangsu Province, China. Article. *Medical Science Monitor*. 2021;27doi:10.12659/MSM.930853  1080. Zhou L, Liu W, Liu K, et al. Clinical characteristics of death and recovered cases of COVID-19 patients with endotracheal intubation. Article. *Acta Medica Mediterranea*. 2021;37(2):833-839. doi:10.19193/0393-6384_2021_2_126  1081. Zhou W, Liu Y, Xu B, et al. Early identification of patients with severe COVID-19 at increased risk of in-hospital death: A multicenter case-control study in Wuhan. Article. *Journal of Thoracic Disease*. 2021;13(3):1380-1395. doi:10.21037/jtd-20-2568  1082. Zhou W, Zhou C, Liu X, et al. A randomised trial on the therapeutic effectiveness of bronchoalveolar lavage under fiberoptic bronchoscopy in patients with severe lung infection living in the Tibetan plateau area. Journal Article; Randomized Controlled Trial. *Annals of palliative medicine*. 2021;10(3):3336‐3342. doi:10.21037/apm-21-470  1083. Zhou X, Cheng Z, Shu D, et al. Characteristics of mortal COVID-19 cases compared to the survivors. *Aging (Albany NY)*. Nov 21 2020;12(24):24579-24595. doi:10.18632/aging.202216  1084. Zhu H, Huang LQ, Qin YL, Zhao XX. To explore the clinical curative effect and mechanism of qingjin zhuyu decoction iontophoresis on COPD. *Journal of tropical medicine*. 2016;16:480‐482.  1085. Zhu J, Yan L, Ji JN, et al. Fluvastatin combined with corbrin capsule can improve depression and anxiety in the treatment of chronic obstructive pulmonary disease. Article. *American Journal of Translational Research*. 2021;13(9):10501-10508.  1086. Zhu QQ, Gong T, Huang GQ, et al. Pulmonary artery trunk enlargement on admission as a predictor of mortality in in-hospital patients with COVID-19. Article. *Japanese Journal of Radiology*. 2021;39(6):589-597. doi:10.1007/s11604-021-01094-9  1087. Zioud F, Marzaioli V, El-Benna J, Bachoual R. Punica granatum and citrillus colocynthis aqueous extracts protect mice from lps-induced lung inflammation and inhibit metalloproteinases-2 and -9. Article. *Indian Journal of Pharmaceutical Education and Research*. 2019;53(3):503-510. doi:10.5530/ijper.53.3.82  1088. Zirpe K, Gurav S, Deshmukh A, Wankhede P. Barotrauma, invasive ventilation, and timing of tocilizumab as predictors of mortality along with inflammatory markers and comorbidities in critically ill COVID-19 patients: A retrospective study. Review. *Indian Journal of Anaesthesia*. 2021;65(10):755-759. doi:10.4103/ija.ija_637_21  1089. Zornić N, Milovanović DR, Stojadinović M, et al. Quality of life of the mechanically ventilated patients with community acquired pneumonia. Article. *Vojnosanitetski Pregled*. 2018;75(9):864-874. doi:10.2298/VSP160905008Z  1090. Zou ZY, Ren D, Chen RL, et al. Persistent lymphopenia after diagnosis of COVID-19 predicts acute respiratory distress syndrome: A retrospective cohort study. Article. *European Journal of Inflammation*. 2021;19doi:10.1177/20587392211036825  1091. Zuo YH, Wang WQ, Chen QJ, et al. Candida in Lower Respiratory Tract Increases the Frequency of Acute Exacerbation of Chronic Obstructive Pulmonary Disease: A Retrospective Case-Control Study. Article. *Frontiers in cellular and infection microbiology*. 2020;10doi:10.3389/fcimb.2020.538005 | |
| Reviews (n =67 ) | 1. Torbay PCT plan to cut hospital admissions. Note. *Pharmaceutical Journal*. 2004;272(7300):631.  2. Procalcitonin test can reduce antibiotic use in COPD. *The Journal of family practice*. Apr 2007;56(4):273.  3. Abstracts of Papers Presented at the Royal Pharmaceutical Society Conference. Conference Review. *International Journal of Pharmacy Practice*. 2010;18  4. An unmet medical need: Rapid molecular diagnostics tests for respiratory tract infections. Article. *Clinical Infectious Diseases*. 2011;52(SUPPL. 4):S384-S395. doi:10.1093/cid/cir055  5. 2013 CAEP/ACMU Scientific Abstracts, CAEP 2013. Conference Review. *Canadian Journal of Emergency Medicine*. 2013;15:S1.  6. Editor’s commentary. Editorial. *Respiratory Care*. 2019;64(5):i.  7. Abedini A, Kiani A, Emami H, Touhidi MH. Serum procalcitonin level as a predictor of bacterial infection in patients with COPD exacerbation. Article. *Tanaffos*. 2019;18(2):112-117.  8. Arellano-Orden E, Calero-Acuña C, Cordero JA, et al. Specific networks of plasma acute phase reactants are associated with the severity of chronic obstructive pulmonary disease: A case-control study. Article. *International Journal of Medical Sciences*. 2017;14(1):67-74. doi:10.7150/ijms.16907  9. Bafadhel M, Clark TW, Reid C, et al. Procalcitonin and C-reactive protein in hospitalized adult patients with community-acquired pneumonia or exacerbation of asthma or COPD. Journal: Article. *Chest*. 2011;139(6):1410‐1418. doi:10.1378/chest.10-1747  10. Baumgartner T, Zurauskaite G, Steuer C, et al. Association of serum sphingomyelin profile with clinical outcomes in patients with lower respiratory tract infections: Results of an observational, prospective 6-year follow-up study. Article. *Clinical Chemistry and Laboratory Medicine*. 2019;57(5):679-689. doi:10.1515/cclm-2018-0509  11. Bhardwaj M, Saini V, Kaur J. Evaluation and Correlation of Serum C-Reactive Protein and Procalcitonin Levels in Acute Exacerbation and Stable Phase of Chronic Obstructive Pulmonary Disease Patients: An Observational Study. Article. *Journal of Clinical and Diagnostic Research*. 2021;15(12):OC24-OC28. doi:10.7860/JCDR/2021/52498.15756  12. Bremmer DN, Moffa MA, Ma K, et al. Acute exacerbations of chronic obstructive pulmonary disease with a low procalcitonin concentration: Impact of antibiotic therapy. Article. *Clinical Infectious Diseases*. 2019;68(5):725-730. doi:10.1093/cid/ciy552  13. Chang C, Yao WZ, Chen YH, Liu ZY, Zhang XW. The changes and clinical implications of serum procalcitonin in acute exacerbations of chronic obstructive pulmonary disease. Article. *Zhonghua jie he he hu xi za zhi = Zhonghua jiehe he huxi zazhi = Chinese journal of tuberculosis and respiratory diseases*. 2006;29(7):444-447.  14. Chen CYJ, Yew MS, Abisheganaden JA, Xu H. Predictors of Influenza PCR Positivity in Acute Exacerbations of Chronic Obstructive Pulmonary Disease. Article. *International Journal of COPD*. 2022;17:25-32. doi:10.2147/COPD.S338757  15. Chi CT. Normal procalcitonin guides antibiotic therapy for acute exacerbation of chronic obstructive pulmonary disease-single center, prospective randomized control study. *<https://trialsearchwhoint/Trial2aspx?TrialID=ChiCTR-TRC-14004726>*. 2014;  16. Dai G, Ran Y, Wang J, et al. Clinical Differences between Eosinophilic and Noneosinophilic Acute Exacerbation of Chronic Obstructive Pulmonary Disease: A Multicenter Cross-Sectional Study. Article. *Mediators of Inflammation*. 2020;2020doi:10.1155/2020/1059079  17. Daniels JM, Schoorl M, Snijders D, et al. Procalcitonin vs C-reactive protein as predictive markers of response to antibiotic therapy in acute exacerbations of COPD. Comparative Study; Journal Article; Randomized Controlled Trial; Research Support, Non‐U.S. Gov't. *Chest*. 2010;138(5):1108‐1115. doi:10.1378/chest.09-2927  18. Daubin C, Fournel F, Thiollière F, et al. Ability of procalcitonin to distinguish between bacterial and nonbacterial infection in severe acute exacerbation of chronic obstructive pulmonary syndrome in the ICU. Article. *Annals of Intensive Care*. 2021;11(1)doi:10.1186/s13613-021-00816-6  19. Daubin C, Parienti JJ, Vabret A, et al. Procalcitonin levels in acute exacerbation of COPD admitted in ICU: A prospective cohort study. Article. *BMC Infectious Diseases*. 2008;8doi:10.1186/1471-2334-8-145  20. El-Azeem AA, Hamdy G, Saraya M, Fawzy E, Anwar E, Abdulattif S. The role of procalcitonin as a guide for the diagnosis, prognosis, and decision of antibiotic therapy for lower respiratory tract infections. Article. *Egyptian Journal of Chest Diseases and Tuberculosis*. 2013;62(4):687-695. doi:10.1016/j.ejcdt.2013.07.017  21. Falsey AR, Becker KL, Swinburne AJ, et al. Utility of serum procalcitonin values in patients with acute exacerbations of chronic obstructive pulmonary disease: A cautionary note. Article. *International Journal of COPD*. 2012;7:127-135. doi:10.2147/COPD.S29149  22. Fowler CL. Procalcitonin for triage of patients with respiratory tract symptoms: A case study in the trial design process for approval of a new diagnostic test for lower respiratory tract infections. Article. *Clinical Infectious Diseases*. 2011;52(SUPPL. 4):S351-S356. doi:10.1093/cid/cir058  23. Fu L, Xiao B, Zhang L, Pan H, Zhong W, Zhu L. Changes in serum STREM-1 and PCT in patients with AECOPD before and after treatment and its correlation with the severity of COPD. Article. *Acta Medica Mediterranea*. 2019;35(4):2075-2079. doi:10.19193/0393-6384_2019_4_326  24. Gao E, Zhang C, Wang J. Effects of budesonide combined with noninvasive ventilation on PCT, sTREM-1, chest lung compliance, humoral immune function and quality of life in patients with AECOPD complicated with type II respiratory failure. Journal: Article. *Open medicine (poland)*. 2019;14(1):271‐278. doi:10.1515/med-2019-0023  25. Haag E, Gregoriano C, Molitor A, et al. Does mid-regional pro-adrenomedullin (MR-proADM) improve the sequential organ failure assessment-score (SOFA score) for mortality-prediction in patients with acute infections? Results of a prospective observational study. Review. *Clinical Chemistry and Laboratory Medicine*. 2021;59(6):1165-1176. doi:10.1515/cclm-2020-1566  26. Huang DT, Yealy DM, Angus DC. Longer-term outcomes of the ProACT trial. Letter. *New England Journal of Medicine*. 2020;382(5):485-486. doi:10.1056/NEJMc1910508  27. Johnson SA, Rupp AB, Rupp KL, Reddy S. Clinical outcomes and costs associated with procalcitonin utilization in hospitalized patients with pneumonia, heart failure, viral respiratory infection, or chronic obstructive pulmonary disease. Article. *Internal and emergency medicine*. 2021;16(3):677-686. doi:10.1007/s11739-020-02618-3  28. Junmin L. Diagnosis and application of serum procalcitonin in lower respiratory tract infections. Article. *Indian Journal of Pharmaceutical Sciences*. 2020;82:1-9.  29. Karhu J, Ala-Kokko TI, Vuorinen T, Ohtonen P, Julkunen I, Syrjälä HT. Interleukin-5, interleukin-6, interferon induced protein-10, procalcitonin and C-reactive protein among mechanically ventilated severe community-acquired viral and bacterial pneumonia patients. Article. *Cytokine*. 2019;113:272-276. doi:10.1016/j.cyto.2018.07.019  30. Kherad O, Kaiser L, Bridevaux PO, et al. Upper-respiratory viral infection, biomarkers, and COPD exacerbations. Article. *Chest*. 2010;138(4):896-904. doi:10.1378/chest.09-2225  31. Koutsokera A, Stolz D, Loukides S, Kostikas K. Systemic biomarkers in exacerbations of COPD: The evolving clinical challenge. Article. *Chest*. 2012;141(2):396-405. doi:10.1378/chest.11-0495  32. Kristoffersen KB, Søgaard OS, Wejse C, et al. Antibiotic treatment interruption of suspected lower respiratory tract infections based on a single procalcitonin measurement at hospital admission--a randomized trial. Journal Article; Randomized Controlled Trial; Research Support, Non‐U.S. Gov't. *Clinical microbiology and infection*. 2009;15(5):481‐487. doi:10.1111/j.1469-0691.2009.02709.x  33. Lacoma A, Prat C, Andreo F, et al. Value of procalcitonin, C-reactive protein, and neopterin in exacerbations of chronic obstructive pulmonary disease. Article. *International Journal of COPD*. 2011;6(1):157-169. doi:10.2147/COPD.S16070  34. Li Y, Xie L, Xin S, Li K. Values of procalcitonin and C-reactive proteins in the diagnosis and treatment of chronic obstructive pulmonary disease having concomitant bacterial infection. Article. *Pakistan Journal of Medical Sciences*. 2017;33(3):566-569. doi:10.12669/pjms.333.12554  35. Li Z, Shu Y, Yu S, et al. Presepsin levels predict to bacterial infection in patients with acute exacerbations of COPD. Article. *Acta Medica Mediterranea*. 2019;35(5):2715-2719. doi:10.19193/0393-6384_2019_5_427  36. Lin TL, Chen WW, Ding ZR, Wei SC, Huang ML, Li CH. Correlations between serum amyloid A, C-reactive protein and clinical indices of patients with acutely exacerbated chronic obstructive pulmonary disease. Article. *Journal of Clinical Laboratory Analysis*. 2019;33(4)doi:10.1002/jcla.22831  37. Lıu X, Deng K, Chen S, et al. 8-Hydroxy-2’-deoxyguanosine as a biomarker of oxidative stress in acute exacerbation of chronic obstructive pulmonary disease. *Turk J Med Sci*. Feb 11 2019;49(1):93-100. doi:10.3906/sag-1807-106  38. Lopez-Campos JL, Calero-Acuna C, Lopez-Ramirez C, et al. Implications of the inflammatory response for the identification of biomarkers of chronic obstructive pulmonary disease. Review. *Biomarkers in Medicine*. 2016;10(2):109-122. doi:10.2217/bmm.15.87  39. Mathioudakis AG. Capsule Commentary on Bremmer et. al., Impact of Procalcitonin Guidance on Management of Adults Hospitalized with Chronic Obstructive Pulmonary Disease Exacerbations. Article in Press. *Journal of General Internal Medicine*. 2018:1. doi:10.1007/s11606-018-4341-x  40. Nct. Procalcitonin as a Marker of Antibiotic Therapy in Patients With Lower Respiratory Tract Infections. *<https://clinicaltrialsgov/show/NCT02171338>*. 2014;  41. Nucera F, Bello FL, Shen SS, et al. Role of atypical chemokines and chemokine receptors pathways in the pathogenesis of copd. Review. *Current Medicinal Chemistry*. 2021;28(13):2577-2653. doi:10.2174/0929867327999200819145327  42. Pandey S, Garg R, Kant S, Verma A, Gaur P. Serum procalcitonin levels in chronic obstructive pulmonary disease patients in North Indian Population. Article. *Annals of African Medicine*. 2019;18(2):103-107. doi:10.4103/aam.aam_44_18  43. Pizzini A, Lunger F, Sahanic A, et al. Diagnostic and Prognostic Value of Inflammatory Parameters Including Neopterin in the Setting of Pneumonia, COPD, and Acute Exacerbations. Article. *COPD: Journal of Chronic Obstructive Pulmonary Disease*. 2017;14(3):298-303. doi:10.1080/15412555.2016.1266317  44. Ruiz-González A, Sáez-Huerta E, Martínez-Alonso M, Bernet-Sánchez A, Porcel JM. A Simple Scoring System to Differentiate Bacterial from Viral Infections in Acute Exacerbations of COPD Requiring Hospitalization. Article. *International Journal of COPD*. 2022;17:773-779. doi:10.2147/COPD.S356950  45. Russi EW, Karrer W, Brutsche M, et al. Diagnosis and management of chronic obstructive pulmonary disease: The swiss guidelines: Official guidelines of the swiss respiratory society. Article. *Respiration*. 2013;85(2):160-174. doi:10.1159/000346025  46. Şahin F, Koşar AF, Aslan AF, Yiğitbaş B, Uslu B. Serum Biomarkers in Patients with Stable and Acute Exacerbation of Chronic Obstructive Pulmonary Disease: A Comparative Study. *J Med Biochem*. Oct 2019;38(4):503-511. doi:10.2478/jomb-2018-0050  47. Shin B, Kim SH, Yong SJ, et al. Early readmission and mortality in acute exacerbation of chronic obstructive pulmonary disease with community-acquired pneumonia. Article. *Chronic Respiratory Disease*. 2019;16doi:10.1177/1479972318809480  48. Soler N, Esperatti M, Ewig S, Huerta A, Agustí C, Torres A. Sputum purulence-guided antibiotic use in hospitalised patients with exacerbations of COPD. *The European respiratory journal*. Dec 2012;40(6):1344-53. doi:10.1183/09031936.00150211  49. Song Y, Liu Y, Zhou Z, Yang W, Zhou Y. The clinical study of serum hs-CRP, TNF-α, PCT and IL-6 in patients with acute exacerbation of chronic obstructive pulmonary disease. Article. *International Journal of Clinical and Experimental Medicine*. 2017;10(9):13550-13556.  50. Stolz D, Bernasconi M, Tamm M. Role of biomarkers in exacerbation of chronic obstructive pulmonary disease. Article. *Clinical Pulmonary Medicine*. 2011;18(3):101-108. doi:10.1097/CPM.0b013e31821750fa  51. Taghizadieh A, Soleimanpour H, Rahmani F, Nia KS, Khodaverdizadeh H. Comparison of serum levels of procalcitonin and c-reactive protein in patients with community acquired pneumonia and COPD exacerbation. Article. *Iranian Red Crescent Medical Journal*. 2016;18(12)doi:10.5812/ircmj.25811  52. Tanrıverdi H, Örnek T, Erboy F, et al. Comparison of diagnostic values of procalcitonin, C-reactive protein and blood neutrophil/lymphocyte ratio levels in predicting bacterial infection in hospitalized patients with acute exacerbations of COPD. Article. *Wiener Klinische Wochenschrift*. 2015;127(19-20):756-763. doi:10.1007/s00508-014-0690-6  53. Taşçi C, Balkan A, Karadurmuş N, et al. The importance of serum procalcitonin levels in patients with chronic obstructive pulmonary disease exacerbations. Article. *Turkish Journal of Medical Sciences*. 2008;38(2):139-144.  54. Titova E, Christensen A, Henriksen AH, Steinshamn S, Åsberg A. Comparison of procalcitonin, C-reactive protein, white blood cell count and clinical status in diagnosing pneumonia in patients hospitalized with acute exacerbations of COPD: A prospective observational study. Article. *Chronic Respiratory Disease*. 2019;16doi:10.1177/1479972318769762  55. Ulrich RJ, McClung D, Wang BR, Winters S, Flanders SA, Rao K. Introduction of Procalcitonin Testing and Antibiotic Utilization for Acute Exacerbations of Chronic Obstructive Pulmonary Disease. Article. *Infectious Diseases: Research and Treatment*. 2019;12doi:10.1177/1178633719852626  56. van de Geijn GJM, Denker S, Meuleman-van Waning V, et al. Evaluation of new laboratory tests to discriminate bacterial from nonbacterial chronic obstructive pulmonary disease exacerbations. Article. *International Journal of Laboratory Hematology*. 2016;38(6):616-628. doi:10.1111/ijlh.12550  57. Wang JX, Zhang SM, Li XH, Zhang Y, Xu ZY, Cao B. Acute exacerbations of chronic obstructive pulmonary disease with low serum procalcitonin values do not benefit from antibiotic treatment: a prospective randomized controlled trial. Journal Article; Randomized Controlled Trial. *International journal of infectious diseases*. 2016;48:40‐45. doi:10.1016/j.ijid.2016.04.024  58. Weerasuriya N, Snape J. A study of candida esophagitis in elderly patients attending a district general hospital in the UK. Article. *Diseases of the Esophagus*. 2006;19(3):189-192. doi:10.1111/j.1442-2050.2006.00563.x  59. Wróblewski T, Marcisz C. Procalcitonin as a biomarker of acute lower respiratory tract infections. Review. *Expert Opinion on Medical Diagnostics*. 2009;3(1):67-79. doi:10.1517/17530050802623859  60. Xiong G, Qiao B, Wu Z, Li Y. Diagnostic values of C-reactive protein, procalcitonin and serum amyloid a in predicting bacterial infection in patients with acute exacerbations of chronic obstructive pulmonary disease. Article. *International Journal of Clinical and Experimental Medicine*. 2018;11(7):7118-7124.  61. Xiong W, Xu M, Zhao Y, Wu X, Pudasaini B, Liu JM. Can we predict the prognosis of COPD with a routine blood test? Article. *International Journal of COPD*. 2017;12:615-625. doi:10.2147/COPD.S124041  62. Xu N, Chen J, Chang X, et al. NCD64 index as a prognostic biomarker for mortality in acute exacerbation of chronic obstructive pulmonary disease. Article. *Annals of Saudi Medicine*. 2016;36(1):37-41. doi:10.5144/0256-4947.2016.37  63. Xu Y, Wu F, Ding H. Influence of soluble myeloid cell trigger receptor-1, procalcitonin, and C-reactive protein on lower respiratory tract bacterial infection in patients with chronic obstructive pulmonary disease. Article. *Acta Medica Mediterranea*. 2019;35(5):2423-2426. doi:10.19193/0393-6384_2019_5_378  64. Yu Z, Zhou L, Wang S, Ding Y. Evaluation of COPD patients with combined detection of serum PA, SAA and PCT levels. Article. *International Journal of Clinical and Experimental Medicine*. 2020;13(12):9898-9903.  65. Zhou SH, Xu XP, Mao ZF, Fang L, Wang XL. Effect of levofloxacin combined with inhalation of large dose ambroxol injection by oxygen on levels of serum ApoE and PCT of elderly patients with COPD complicated with pulmonary infections. *Chinese journal of nosocomiology*. 2018;28:848‐851.  66. Zhou W, Tan J. The expression and the clinical significance of eosinophils, PCT and CRP in patients with acute exacerbation of chronic obstructive pulmonary disease complicated with pulmonary infection. Article. *American Journal of Translational Research*. 2021;13(4):3451-3458.  67. Zou Y, Zhu Z, Zhang Y. Significance of serum procalcitonin combined with C-reactive protein in diagnosis of acute exacerbation of chronic obstructive pulmonary disease and guidance of antibiotics therapy. *International journal of clinical and experimental medicine*. 2018;11(10):11070‐11078. | |
| Abstracts (n = 31) | 1. Agmy G, Gad Y, Helal S. Clinical implication of serum procalcitonin in acute exacerbations of chronic obstructive pulmonary disease. Conference Abstract. *Chest*. 2011;140(4)doi:10.1378/chest.1089758  2. Bi YY, Wang ZS, Li YZ. The changes and clinical implications of serum procalcitonin and C-reactive protein in acute exacerbations of chronic obstructive pulmonary disease. Conference Abstract. *Respirology*. 2011;16:101-102. doi:10.1111/j.1400-1843.2011.02071.x  3. Blairon L, De Villenfagne MA, Dahma H, Mols P, Dediste A, Vandenberg O. Utility of unique procalcitonin dosage in patients with acute exacerbation of chronic obstructive pulmonary disease in emergency wards. Conference Abstract. *Clinical Microbiology and Infection*. 2009;15:S190-S191. doi:10.1111/j.1469-0691.2009.02858.x  4. Borsi H, Nia EP, Mal-Amir MD, Raji H. Relationship between serum procalcitonin level and chronic obstructive pulmonary disease. *Journal of family medicine and primary care*. Feb 2019;8(2):738-740. doi:10.4103/jfmpc.jfmpc_468_18  5. Burke L, Alhajji M, Todd N, S White J. Procalcitonin (PCT) is a safe and reliable biomarker of bacterial infection in exacerbations of copddso why is it so challenging to introduce it into a large UK hospital? Conference Abstract. *Thorax*. 2010;65:A125-A126. doi:10.1136/thx.2010.150987.14  6. Çetinkaya A, Uysal MA, Niksarlioǧlu EY, Durna AS, Özer NO, Çamsari G. Can neutrophil/lymphocyte ratio, C-reactive protein (CRP) and procalcitonin predict the hospitalization time in patients with lower tract respiratory Infections? Conference Abstract. *Turkish Thoracic Journal*. 2019;20:S126. doi:10.5152/TurkThoracJ.2019.126  7. Clark T, Bafadhel M, Reid C, et al. Serum procalcitonin and C-reactive protein in patients hospitalised with pneumonia, asthma or COPD. Conference Abstract. *Thorax*. 2009;64:A118. doi:10.1136/thx.2009.127159z  8. Çolak AA, Doʇan N, Aktoʇu S, et al. PCT and CRP levels patients with community acquired pneumonia or exacerbation of COPD. Conference Abstract. *Turkish Journal of Biochemistry*. 2013;38  9. Costales JK, Nomura JH, Joanie Chung WL, Ironside K, Sim JJ, Salama P. Initial procalcitonin level and antibiotic practice patterns among patients with acute respiratory infections. Conference Abstract. *Open Forum Infectious Diseases*. 2019;6:S668. doi:10.1093/ofid/ofz360.1672  10. Crisafulli E, Huerta A, Guerrero M, et al. Treatment failure of hospitalized patients with COPD exacerbation using systemic corticosteroids plus antibiotics: Role of early inflammatory predictors. Conference Abstract. *European Respiratory Journal*. 2014;44  11. Daubin C, Fournel F, Allouche S, et al. Procalcitonin fail to distinguish between bacterial and viral infection in severe AECOPD. Conference Abstract. *Annals of Intensive Care*. 2019;9doi:10.1186/s13613-018-0474-7  12. Daubin C, Parienti JJ, Fradin S, et al. Procalcitonin levels and bacterial aetiology among COPD patients admitted to the ICU with severe pneumonia: A prospective cohort study. Article. *BMC Infectious Diseases*. 2009;9:157. doi:10.1186/1471-2334-9-157  13. Eraslan Doganay G, Cirik MO. Determinants of prognosis in geriatric patients followed in respiratory ICU; either infection or malnutrition. *Medicine (Baltimore)*. Sep 10 2021;100(36):e27159. doi:10.1097/md.0000000000027159  14. Gough C, Pillai P, Mulla R. Our experience of procalcitonin assay in identifying bacterial COPD exacerbations. Conference Abstract. *European Respiratory Journal*. 2011;38  15. Habel M, Brazeau D. Use of procalcitonin in the acute exacerbation of chronic obstructive pulmonary disease in the emergency room : A costs/benefits and hospital practice short study. Conference Abstract. *Clinical Chemistry and Laboratory Medicine*. 2015;53:S564. doi:10.1515/cclm-2015-5016  16. Hassaan K, Mohamed M, Ramadan E, Hashim M, Sharaf S. Procalcitonin as a diagnostic marker in acute exacerbation of COPD. Conference Abstract. *European Respiratory Journal*. 2012;40  17. Huerta A, Crisafulli E, Menendez R, et al. Pneumonic and non-pneumonic exacerbations of COPD: Systemic inflammatory response and clinical characteristics. Conference Abstract. *European Respiratory Journal*. 2013;42  18. Jeong SW, Lee SY, Yoo SS, et al. Comparison of clinical features and outcomes between community-acquired pneumonia and acute exacerbation in patients with chronic obstructive pulmonary disease. Conference Abstract. *Respirology*. 2009;14:A214. doi:10.1111/j.1440-1843.2009.01658.x  19. Kawamatawong T, Jintanalert W. Serum procalcitonin (PCT-Q) as a diagnostic tool for bacterial lower respiratory tract infection among COPD patients with acute exacerbation. Conference Abstract. *European Respiratory Journal*. 2013;42  20. Lin SH, He YP, Lian JJ, Chu CK. Procalcitonin kinetics to guide sequential invasive-noninvasive mechanical ventilation weaning in patients with acute exacerbation of chronic obstructive pulmonary disease and respiratory failure: procalcitonin's adjunct role. Journal Article; Randomized Controlled Trial. *Libyan journal of medicine*. 2021;16(1):1961382. doi:10.1080/19932820.2021.1961382  21. Medveczky T, Jackson M. Procalcitonin use in acute exacerbations of COPD. Conference Abstract. *European Respiratory Journal*. 2011;38  22. Patel N, Jones P, Adamson V, Thorpe G, Belcher J, Spiteri M. Evaluation of saliva biomarkers as indicators of health status and exacerbations in copd. Conference Abstract. *Thorax*. 2014;69:A102-A103. doi:10.1136/thoraxjnl-2014-206260.205  23. Pizzini A, Sahanic A, Kurz K, Weiss G, Bellmann-Weiler R. Diagnostic and prognostic value of inflammatory parameters including neopterin in pneumonia, COPD and acute exacerbations of COPD. Conference Abstract. *Pneumologie*. 2016;70(5)doi:10.1055/s-0036-1583511  24. Rader W, Petite S, Murphy J. Assessment of the use of procalcitonin in hospitalized, non-critically ill patients with chronic obstructive pulmonary disease exacerbations. Conference Abstract. *JACCP Journal of the American College of Clinical Pharmacy*. 2020;3(8):1638-1639. doi:10.1002/jac5.1351  25. Rovina N, Dima E, Gennimata SA, Vakali S, Koutsoukou A, Koulouris N. Neutrophil/lymphocyte (NLR) ratio in infectious acute exacerbations of COPD (AECOPD). Conference Abstract. *European Respiratory Journal*. 2014;44  26. Shang L. Serum concentrations of HMGB1, STREM1 and inflammatory cytokines correlate with pulmonary function changes at moderate altitude in patients with COPD exacerbations. Conference Abstract. *High Altitude Medicine and Biology*. 2016;17(3):250. doi:10.1089/ham.2016.29011.abstracts  27. Stolz D, Blasi F, Louis R, et al. Relation of health-related quality of life, frequent exacerbation phenotype and circulating systemic biomarkers in stable COPD. Conference Abstract. *European Respiratory Journal*. 2011;38  28. Tanriverdi H, Tor MM, Atalay F, et al. Relationship between procacitonin and C-reactive protein and sputum culture results in patients with acute excecerbation of COPD. Conference Abstract. *European Respiratory Journal*. 2014;44  29. Titova EV, Lofblad L, Asberg A, Henriksen AH. Calprotectin as a biomarker of pneumonia in patients hospitalized with AECOPD. Conference Abstract. *European Respiratory Journal*. 2019;54doi:10.1183/13993003.congress-2019.PA2895  30. Wang F, Yang L, Wang M, He B. Low blood eosinophil count is associated with gram-positive cocci infection and poor clinical outcomes in severe exacerbations of COPD. Conference Abstract. *European Respiratory Journal*. 2020;56doi:10.1183/13993003.congress-2020.4719  31. Xu H, Yew MS, Tan JH, Abisheganaden J. Predictors of influenza PCR positivity in acute exacerbations of chronic obstructive pulmonary disease. Conference Abstract. *European Respiratory Journal*. 2020;56doi:10.1183/13993003.congress-2020.2377 | |
| **Studies excluded in full-text review with reasons (n = 22)** | | |
| Conference abstract(n=5) | 1. Telgen MC, Brusse-Keizer MGJ, Van Der Valk PDLPM, Van Der Palen J, Kerstjens HAM, Hendrix MGR. Procalcitonin and proadrenomedullin in COPD patients: Stable state versus exacerbation difference in mortality rate? European Respiratory Journal. 2012;40. 2. Topeli A, Ergan Arsava B. Prognostic value of procalcitonin and its role in identification of bacterial infections in exacerbations of COPD requiring mechanical ventilation. American Journal of Respiratory and Critical Care Medicine. 2010;181(1). 3. Ocakli B, Tuncay E, Gungor S, Sertbas M, Adiguzel N, Irmak I, et al. Inflammatory Markers in Patients Using Domiciliary Non-invasive Mechanical Ventilation: C Reactive Protein, Procalcitonin, Neutrophil Lymphocyte Ratio. Frontiers in public health. 2018;6:245. 4. Patel N, Thorpe G, Jones P, Adamson V, Belcher J, Spiteri MA. Levels of salivary C-reactive protein, procalcitonin and neutrophil elastase can predict exacerbations in copd and determine those patients at high risk of re-exacerbation. Thorax. 2016;71:A39-A40. 5. Huang X, Zhang J, Huang X, Chen Y, Zeng M. Usefulness of procalcitonin as a prognostic marker in critically ill patients with acute exacerbation of chronic obstructive pulmonary disease. Chest. 2016;149(4):A366. | |
| Missing information(n=4) | 1. El Halim AA, Sayed M. The value of serum procalcitonin among exacerbated COPD patients. Egyptian Journal of Chest Diseases and Tuberculosis. 2015;64(4):821-7.<https://doi.org/10.1016/j.ejcdt.2015.05.016>. 2. Wang H, Yang T, Yu X, Chen Z, Ran Y, Wang J, et al. Risk Factors for Length of Hospital Stay in Acute Exacerbation Chronic Obstructive Pulmonary Disease: A Multicenter Cross-Sectional Study. International Journal of General Medicine. 2022;15:3447-58.<https://doi.org/10.2147/IJGM.S354748>. 3. Yang L, Li M, Shu J, Yang Y, Huang Q. A risk prediction model for prolonged length of stay in patients with acute exacerbations of chronic obstructive pulmonary disease: A retrospective study of 225 patients in a single center in Kunming China. Medical Science Monitor. 2021;28.<https://doi.org/10.12659/MSM.934392>.   Chen Y, Li LQ, Ge YL, Hu XY, Zhang Q, Zhan HF, et al. Procalcitonin (PCT) improves the accuracy and sensitivity of dyspnea, eosinopenia, consolidation, acidemia and Atrial Fibrillation (DECAF) score in predicting AECOPD patients admission to ICU. Clinical Laboratory. 2020;66(3):287-96.<https://doi.org/10.7754/CLIN.LAB.2019.190612>. | |
| Did not meet criteria(n=13) | 1. Chang CH, Tsao KC, Hu HC, et al. Procalcitonin and C-reactive protein cannot differentiate bacterial or viral infection in COPD exacerbation requiring emergency department visits. Article. *International Journal of COPD*. 2015;10:767-774. doi:10.2147/COPD.S76740  2. Crisafulli E, Torres A, Huerta A, et al. Predicting In-Hospital Treatment Failure (≤7 days) in Patients with COPD Exacerbation Using Antibiotics and Systemic Steroids. Article. *COPD: Journal of Chronic Obstructive Pulmonary Disease*. 2016;13(1):82-92. doi:10.3109/15412555.2015.1057276  3. Kawamatawong T, Apiwattanaporn A, Siricharoonwong W. Serum inflammatory biomarkers and clinical outcomes of COPD exacerbation caused by different pathogens. Article. *International Journal of COPD*. 2017;12:1625-1630. doi:10.2147/COPD.S132132  4. Liu L, Luan Y, Xiao L, Wang N, Wang J, Cui Z. The predictive value of serum procalcitonin for non-invasive positive pressure ventilation in the patients with acute exacerbation of chronic obstructive pulmonary disease. Article. *Medicine*. 2021;100(16):e25547. doi:10.1097/MD.0000000000025547  5. Lotfy S, Zayed N, Moghawri M, Fouad R. Serum procalcitonin level as a predictor of NIPPV and mortality in patients with COPD at Zagazig University Hospitals. Article. *Egyptian Journal of Chest Diseases and Tuberculosis*. 2019;68(2):170-174. doi:10.4103/ejcdt.ejcdt_136_18  6. Meier MA, Ottiger M, Vogeli A, et al. Activation of the Serotonin Pathway is Associated with Poor Outcome in COPD Exacerbation: results of a Long-Term Cohort Study. Journal: Article. *Lung*. 2017;195(3):303‐311. doi:10.1007/s00408-017-0004-7  7. Mohamed HA, Abdelfattah MT, Mohammad MA, Kasem AH, Ali NM. Evaluation of the diagnostic utility of procalcitonin and some old tools in prediction of the need for noninvasive mechanical ventilation in acute exacerbation chronic obstructive pulmonary disease. Article. *Egyptian Journal of Chest Diseases and Tuberculosis*. 2020;69(1):72-79. doi:10.4103/ejcdt.ejcdt_74_19  8. Pazarli AC, Koseoglu HI, Doruk S, et al. Procalcitonin: Is it a predictor of noninvasive positive pressure ventilation necessity in acute chronic obstructive pulmonary disease exacerbation? Article. *Journal of Research in Medical Sciences*. 2012;17(11):1047-1051.  9. Shao XQ, Chen YB, Sun Y, Li N, Chen DC. Diagnostic and prognostic value of solublefms-like tyrosine kinase 1 in patients with AECOPD accompanied with pneumonia. Article. *Journal of Xi'an Jiaotong University (Medical Sciences)*. 2019;40(4):583-587 and 645. doi:10.7652/jdyxb201904018  10. Sun W, Luo Z, Jin J, Cao Z, Ma Y. The neutrophil/lymphocyte ratio could predict noninvasive mechanical ventilation failure in patients with acute exacerbation of chronic obstructive pulmonary disease: A retrospective observational study. Article. *International Journal of COPD*. 2021;16:2267-2277. doi:10.2147/COPD.S320529  11. Wang J, Shang H, Yang X, Guo S, Cui Z. Procalcitonin, C-reactive protein, PaCO2, and noninvasive mechanical ventilation failure in chronic obstructive pulmonary disease exacerbation. Article. *Medicine (United States)*. 2019;98(17)doi:10.1097/MD.0000000000015171  12. Wu LQ, Yang J, Tong CR, Yu YS, Zhang XW. [Analysis of factors associated with failure of sequential mechanical ventilation in patients with acute exacerbation of chronic obstructive pulmonary disease complicated with respiratory failure]. *Zhonghua Jie He He Hu Xi Za Zhi*. Sep 12 2018;41(9):714-717. doi:10.3760/cma.j.issn.1001-0939.2018.09.012  13. Zhu JJ, Liu LJ. Analysis of factors influenced by the effectiveness of non-invasive ventilation in the treatment of acute exacerbation of chronic obstructive pulmonary disease with different severities. Article. *European Review for Medical and Pharmacological Sciences*. 2016;20(22):4775-4781. | |
| **Studies included(n=14)** | | |
| Included | | Citations |
|  | | 1. Stolz D, Christ-Crain M, Morgenthaler NG, Miedinger D, Leuppi J, Müller C, et al. Plasma pro-adrenomedullin but not plasma pro-endothelin predicts survival in exacerbations of COPD. Chest. 2008;134(2):263-72.<https://doi.org/10.1378/chest.08-0047>. 2. Rammaert B, Verdier N, Cavestri B, Nseir S. Procalcitonin as a prognostic factor in severe acute exacerbation of chronic obstructive pulmonary disease. Respirology. 2009;14(7):969-74.<https://doi.org/10.1111/j.1440-1843.2009.01597.x>. 3. Zuur-Telgen MC, Brusse-Keizer MGJ, VanderValk PDLPM, Van Der Palen J, Kerstjens Dr HAM, Hendrix Dr MGR. Stable-state midrange-proadrenomedullin level is a strong predictor of mortality in patients with COPD. Chest. 2014;145(3):534-41.<https://doi.org/10.1378/chest.13-1063>. 4. Kutz A, Briel M, Christ-Crain M, Stolz D, Bouadma L, Wolff M, et al. Prognostic value of procalcitonin in respiratory tract infections across clinical settings. Critical care (London, England). 2015;19:74.<https://doi.org/10.1186/s13054-015-0792-1>. 5. Grolimund E, Kutz A, Marlowe RJ, Vogeli A, Alan M, Christ-Crain M, et al. Long-term Prognosis in COPD Exacerbation: Role of Biomarkers, Clinical Variables and Exacerbation Type. Copd. 2015;12(3):295-305.<https://doi.org/10.3109/15412555.2014.949002>. 6. Ergan B, Sahin AA, Topeli A. Serum Procalcitonin as a Biomarker for the Prediction of Bacterial Exacerbation and Mortality in Severe COPD Exacerbations Requiring Mechanical Ventilation. Respiration. 2016;91(4):316-24.<https://doi.org/10.1159/000445440>. 7. Flattet Y, Garin N, Serratrice J, Perrier A, Stirnemann J, Carballo S. Determining prognosis in acute exacerbation of COPD. International Journal of COPD. 2017;12:467-75.<https://doi.org/10.2147/COPD.S122382>. 8. Yao C, Wang L, Shi F, Chen R, Li B, Liu W, et al. Optimized combination of circulating biomarkers as predictors of prognosis in AECOPD patients complicated with heart failure. International Journal of Medical Sciences. 2021;18(7):1592-9.<https://doi.org/10.7150/ijms.52405>. 9. Stolz D, Christ-Crain M, Morgenthaler NG, Leuppi J, Miedinger D, Bingisser R, et al. Copeptin, C-reactive protein, and procalcitonin as prognostic biomarkers in acute exacerbation of COPD. Chest. 2007;131(4):1058-67.<https://doi.org/10.1378/chest.06-2336>. 10. Ceylan O, Ucgun I, Us T, Kasifoglu N, Kiremitci A, Demircan F, et al. The role of serum procalcitonin and CRP levels in determining of etiology and outcome in acute exacerbations of COPD. Intensive Care Medicine Experimental. 2015;3.<https://doi.org/10.1186/2197-425X-3-S1-A796>. 11. Gong C, Yang Y, Chen M, Xie Z. Effect of procalcitonin on the prognosis of patients with COPD. Biomedical reports. 2020;12(6):313‐8.<https://doi.org/10.3892/br.2020.1298>. 12. Yu X, Zhu GP, Cai TF, Zheng JY. Establishment of risk prediction model and risk score for in-hospital mortality in patients with AECOPD. Clinical Respiratory Journal. 2020;14(11):1090-8.<https://doi.org/10.1111/crj.13246>.   36. Galani M, Kyriakoudi A, Filiou E, Kompoti M, Lazos G, Gennimata SA, et al. Older age, disease severity and co-morbidities independently predict mortality in critically ill patients with copd exacerbation. Pneumon. 2021;34(2).<https://doi.org/10.18332/pne/139637>.  14. Koç Ç, Şahin F. What Are the Most Effective Factors in Determining Future Exacerbations, Morbidity Weight, and Mortality in Patients with COPD Attack? Medicina (Kaunas, Lithuania). 2022;58(2).<https://doi.org/10.3390/medicina58020163>. |
