# Supplementary material for: Prognostic value of procalcitonin in acute exacerbation of chronic obstructive pulmonary disease: A systematic review and meta-analysis
Source: PLoS One. 2024 Dec 30;19(12):e0312099. doi: 10.1371/journal.pone.0312099 (PMC11684632; doi:10.1371/journal.pone.0312099)
Supplement: S3 Table — (DOCX) [file pone.0312099.s005.docx]

S3 Table. Raw data used in current meta-analysis (**Name of data extractors**: Lin Changyang and Li Gaigai; **Date of data extraction**: October 14, 2023; All authors confirmed that the studies below were eligible to be included in the review)

|  | comparison |  | | Outcome(OR，95%CI) | | | | | |  |
| --- | --- | --- | --- | --- | --- | --- | --- | --- | --- | --- |
| Stolz2008 | PCT predict mortality |  | | 1.68[0.96,2.93] | | | | | |  |
| Rammaert 2009 | PCT predict mortality |  | | 1.02[1.00,1.03] | | | | | |  |
| Zuur-Telgen 2014 | PCT predict mortality |  | | 2.24[0.91,5.52] | | | | | |  |
| Kutz 2015 | PCT predict mortality |  | | 6.12[2.46,15.23] | | | | | |  |
| Grolimund 2015 | PCT predict mortality |  | | 1.50[1.10,2.04] | | | | | |  |
| Ergan 2016 | PCT predict mortality |  | | 1.85[1.07,3.20] | | | | | |  |
| Flattet 2017 | PCT predict mortality |  | | 1.02[1.00,1.04] | | | | | |  |
| YaoC 2021 |  |  | | 1.00[0.97,1.03] | | | | | |  |
|  | | | | | | | | | | |
|  | outcome | PCT positive group | | | | PCT negative group | | | | |
|  |  | event | | total | | event | | total | | |
| Stolz 2007 | mortality | 2 | | 31 | | 3 | | 136 | | |
| Rammaert 2009 | mortality | 19 | | 52 | | 11 | | 64 | | |
| Ceylan 2015 | mortality | 5 | | 18 | | 1 | | 40 | | |
| Ergan 2016 | mortality | 10 | | 27 | | 5 | | 36 | | |
|  | | | | | | | | | | |
|  |  | mortality group | | | | survival group | | | | |
|  |  | mean | sd | | n | mean | sd | | n | |
| Galani 2021 | PCT levels | 1.3 | 1.1 | | 45 | 0.93 | 0.97 | | 82 | |
| Koc 2022 | PCT levels | 1.1 | 2.2 | | 24 | 0.7 | 0.7 | | 136 | |
| Kult 2015 | PCT levels | 1.06 | 0.25 | | 17 | 0.23 | 0.1 | | 567 | |
| Yu X 2024 | PCT levels | 1.2 | 2.2 | | 42 | 0.7 | 4.8 | | 653 | |
|  | | | | | | | | | | |
