# Supplementary material for: Prognostic value of procalcitonin in acute exacerbation of chronic obstructive pulmonary disease: A systematic review and meta-analysis
Source: PLoS One. 2024 Dec 30;19(12):e0312099. doi: 10.1371/journal.pone.0312099 (PMC11684632; doi:10.1371/journal.pone.0312099)
Supplement: S4 Table — (DOC) [file pone.0312099.s006.doc]

S4 Table. Risk of bias for each study

| Study | Selection Bias | Comparability | Outcome Bias | Final ROB |
| --- | --- | --- | --- | --- |
| Stolz 2007 | 4 | 1 | 3 | 8 |
| Stolz 2008 | 4 | 2 | 3 | 9 |
| Rammaert 2009 | 4 | 2 | 3 | 9 |
| Zuur-Telgen2014 | 4 | 2 | 3 | 9 |
| Ceylan2015 | 3 | 0 | 1 | 4 |
| Grolinund 2015 | 4 | 2 | 2 | 8 |
| Kutz 2015 | 4 | 1 | 1 | 6 |
| Ergan 2016 | 4 | 1 | 3 | 8 |
| Flattet 2017 | 3 | 1 | 2 | 6 |
| Gong 2020 | 3 | 1 | 3 | 7 |
| Yu 2020 | 4 | 1 | 3 | 8 |
| Galani2021 | 3 | 1 | 3 | 7 |
| Yao2021 | 3 | 1 | 3 | 7 |
| Koc 2022 | 4 | 1 | 2 | 7 |
