# Supplementary material for: Prognostic value of procalcitonin in acute exacerbation of chronic obstructive pulmonary disease: A systematic review and meta-analysis
Source: PLoS One. 2024 Dec 30;19(12):e0312099. doi: 10.1371/journal.pone.0312099 (PMC11684632; doi:10.1371/journal.pone.0312099)
Supplement: Attachment — Submitted filename: Response to Reviewers.docx [file pone.0312099.s008.docx]

Dear Editors and Reviewers:

Thank you for your letter and for the reviewers’ comments concerning our manuscript entitled “Prognostic Value of Procalcitonin in Acute Exacerbation of Chronic Obstructive Pulmonary Disease: A Systematic Review and Meta-analysis” (ID: PONE-D-24-13109). Those comments are all valuable and very helpful for revising and improving our paper, as well as the important guiding significance to our researches. We have studied comments carefully and have made correction which we hope meet with approval. Revised portion are marked in red in the paper. The main corrections in the paper and the responds to the reviewer’s comments are as flowing:

Responds to the reviewer’s comments:

1.	In the introduction (Line 51), grammatical errors are there. ‘Negatively impacts on health status’. It should be ‘negative impact on health’.

Response: We are very sorry for our negligence of grammar. And we have made modifications in the manuscript. To rectify the situation, we have carefully reviewed the entire document and implemented the necessary changes to correct the grammatical issues.

2.	In the results, 8 clinical trials with 2085 patients are mentioned however, the authors have mentioned that fourteen clinical trials were taken into consideration for meta- analysis in the discussion. Pl clarify.

Response: We deeply apologize for any misunderstandings caused by our expression. To comprehensively explore the correlation between PCT levels and mortality in AECOPD patients, we conducted this meta-analysis and included 14 high-quality studies.

Of these, 8 studies explored the potential relationship between PCT levels and mortality risk in AECOPD patients by multivariate analysis, such as age, gender, underlying disease, etc. The results of these studies are usually presented in the form of risk ratio (HR) or odds ratio (OR).

Five studies conducted comparative studies by PCT level grouping. In these studies, patients were divided into PCT positive and negative groups, and the mortality rates between the two groups were compared. This method can directly observe the impact of PCT levels on the survival status of patients.

In addition, four studies directly compared the PCT levels of deceased patients and survivors. These studies revealed the potential relationship between PCT levels and patient survival status by collecting and analyzing PCT data from two groups of patients.

In the results section of the meta-analysis, we analyzed different situations mentioned above. For the convenience of reading and understanding, we have made revisions in the results section. Special thanks to you for your good comments.

3.	The discussion is not lucid as the authors have not clearly explained the results. The discussion meanders a bit and should be a bit more elaborate.

Response: We have made correction according to the Reviewer’s comments.

4. The authors have contradicted themselves. In the results, it is mentioned that no significant association between PCT levels and mortality was found. However, in the conclusion the authors have mentioned that PCT at the time of admission positively corelated with mortality. Please explain and clarify.

Response: We have re-written the result and discussion sections according to the Reviewer’s suggestion. In our meta-analysis, the results showed that mortality is significantly higher in PCT positive group, and the PCT levels of deceased patients are significantly higher than those of survivors.

However, the results determined that PCT levels in patients admitted to the hospital for AECOPD are not associated with increased risk of mortality(RR 1.03, 95%CI 0.99-1.08). Subgroup analysis showed PCT levels are associated with an increased risk of mortality in mild to moderate AECOPD(RR 1.74, 95%CI 1.11-2.73).

Therefore, we have changed the conclusion section to”Results from this meta-analysis suggest that procalcitonin at the time of hospital admission for mild to moderate AECOPD is positively correlated with mortality”.

5.	The Forest plots have not been labelled.

Response: We have bolded the font in the citation section, and inserted figure captions in the text of the manuscript, immediately following the paragraph in which the figure is first cited.

6.	The authors have neither mentioned about the findings of sub-group analysis (as shown in the Forest plot) in the results nor have they discussed them in the discussion section.

Response: It is really true as Reviewer suggested that the absence of subgroup analysis in our study is a notable limitation. Therefore, we have conducted subgroup analysis based on severity of the condition in our revised manuscript.

We tried our best to improve the manuscript and made some changes in the manuscript. These changes will not influence the content and framework of the paper. And here we did not list the changes but marked in red in revised paper.

We appreciate for Editors/Reviewers’ warm work earnestly, and hope that the correction will meet with approval.

Once again, thank you very much for your comments and suggestions.
