# Supplementary material for: Prognostic value of procalcitonin in acute exacerbation of chronic obstructive pulmonary disease: A systematic review and meta-analysis
Source: PLoS One. 2024 Dec 30;19(12):e0312099. doi: 10.1371/journal.pone.0312099 (PMC11684632; doi:10.1371/journal.pone.0312099)
Supplement: Attachment — Submitted filename: Response to Reviewers.docx [file pone.0312099.s009.docx]

Dear Editors and Reviewers:

We quite appreciate your favorite consideration and the reviewer’s insightful comments concerning our manuscript entitled “Prognostic Value of Procalcitonin in Acute Exacerbation of Chronic Obstructive Pulmonary Disease: A Systematic Review and Meta-analysis” (ID: PONE-D-24-13109R1). Now We have revised the manuscript exactly according to the reviewer’s comments, and found these comments are very helpful. We hope this revision can make my paper more acceptable. The revisions were addressed point by point below.

Responds to the reviewer’s comments:

1. Line 81-82, mention “ the type of 82 study design was observational research”, table mention study design retrospective, prospective cohort or cohort study. Line 131 – 132 mention “Eight clinical trials [14-21] with a total of 2085 patients reported the level of PCT predicting mortality for patients with AECOPD”

Response: We are very sorry for our lack of rigor. And we have made modifications in the manuscript.

2.	Please clarify and modify in table whether clinical setting “Hospital” represent out patient department or hospitalized or both? Mild exacerbations usually donot require hospitalization. Hence, it will be good to include more clear information where the study was conducted – out patient department or hospitalized or both. This is essential, as authors concluded that PCT was correlated with increased mortality in mild to moderate.

Response: We deeply apologize for such a misunderstanding. All articles in our current meta-analysis included patients hospitalized for AECOPD. For the convenience of reading and understanding, we have made revisions in the table.

3. Authors need to review for typo-errors

Response: We have made revisions to the entire text according to the Reviewer’s comments.

In all, we found the reviewer’s comments are quite helpful, and revised my paper point-by-point. Thank you and the review again for your help!
