# Supplementary material for: Prognostic value of procalcitonin in acute exacerbation of chronic obstructive pulmonary disease: A systematic review and meta-analysis
Source: PLoS One. 2024 Dec 30;19(12):e0312099. doi: 10.1371/journal.pone.0312099 (PMC11684632; doi:10.1371/journal.pone.0312099)
Supplement: Attachment — Submitted filename: Response to Reviewers.docx [file pone.0312099.s010.docx]

Dear Editors and Reviewers:

We quite appreciate your favorite consideration and the reviewer’s insightful comments concerning our manuscript entitled “Prognostic Value of Procalcitonin in Acute Exacerbation of Chronic Obstructive Pulmonary Disease: A Systematic Review and Meta-analysis” (ID: PONE-D-24-13109R1). Now We have revised the manuscript exactly according to the reviewer’s comments, and found these comments are very helpful. We hope this revision can make my paper more acceptable. The revisions were addressed point by point below.

Responds to the reviewer’s comments:

1.line 33 (abstract) And deceased patients had 1-" remove And before the sentence,"line 50 pulmonary disease (AECOPD) play a singnificant role in the management of COPD, due to their " replace "their" with "its","line 167 (dicussion) please mention p-value when word "significant" is used

Response: We are very sorry for our lack of rigor. And we have made modifications in the manuscript.

2. MOOSE is a checklist for reporting meta analyses. The authors need to assess the risk of bias in the studies using an appropriate tool (see Cochrane ROBINS for example). they also say "The reviewers assessed the quality of the included studies according to Modifying the MOOSE item list." this needs to be corrected for grammar and also they need to explain why they had to use a modified version and who modified it.

Response: Our meta-analysis was performed in accordance with the Preferred Reporting Items for Systematic Reviews and Meta-analyses(PRISMA) statement. According to your comments, we have added the MOOSE checklist.

3.In the statitical analyses section, list all the outcomes, primary and secondary, for clarity. Also are all outcomes reported in exactly the same scale, so there is no need for meta analysing as SMDs? please state that clearly as well. UPDATE: I see in the results SMDs are reported, that needs to be explained in the methods section.

Response: We have made correction according to the Reviewer’s comments, including the “Study selection” and “Statistical analysis” sections.

4.Publication bias is one of the biggest risks in conducting meta analyses and it seems to be ignored. Publication bias tests and plots only relevant if you have >10 studies otherwise underpowered to detect much and tend to lead to conclusions that are not justified http://www.ncbi.nlm.nih.gov/pubmed/11106885. If you don’t have enough studies to assess you should discuss this as a major limitation. Even with 10 or 20 studies it is very difficult to visually assess. If you have 20 or more studies it is a considerable strength.

Response: Thank you for your precious opinions, we have removed the funnel plot and discussed the publication bias.

5.Avoid fixed effect models since they under-perform in the presence of ANY heterogeneity. Random-effects (RE) models are more conservative and provide better estimates with wider confidence intervals. What does the arbitrary 50% cut-off point add? Why is 49% fine and 51% an issue? My point is that a RE model will work better, in the presence of 5% heterogeneity, compared to a FE model!

Response: We have made correction according to the Reviewer’s comments. However, we still choose the fixed effects model when there is no heterogeneity present(I^2^=0).(**Figure 4**)

6.Report the confidence intervals for I^2^, since it is an estimate. A simple formula exists in the seminal 2002 Higgins paper that proposed I^2^.

Response: We have reported the confidence intervals for I^2^ according to the Reviewer’s comments.

7. "Subgroup analysis was performed based on the severity of disease" please expand to clarify, what were the grouping for example.

Response: We have modified and clarified in “Statistical analysis” section. “Subgroup analysis was performed based on the severity of disease. Severe cases are defined as requiring intubation or mechanical ventilation, and others as mild to moderate.”

8. Year may be worth considering in bias assessment, especially if you don't have enough studies for a formal test. With newer studies we would be more confident.

Response: Thanks for your comments, we also agree that year is worth considering in bias assessment. However, there has been no significant change in the diagnostic criteria for COPD, and PCT and death are objective indicators. We believe that it is not a determining factor in bias assessment.

9. How was the random-effect model implemented, i.e. how was heterogeneity estimated? There are numerous ways to do so. Did they use the standard DerSimonian-Laird method? If so, please state so.

Response: We conducted this meta-analysis using Review Manager Software 5.3. RevMan implements a version of random-effects meta-analysis that is described by DerSimonian and Laird method.

We appreciate for Editors/Reviewers’ warm work earnestly, and hope that the correction will meet with approval.

Once again, thank you very much for your comments and suggestions.
