# Supplementary material for: Prognostic value of procalcitonin in acute exacerbation of chronic obstructive pulmonary disease: A systematic review and meta-analysis
Source: PLoS One. 2024 Dec 30;19(12):e0312099. doi: 10.1371/journal.pone.0312099 (PMC11684632; doi:10.1371/journal.pone.0312099)
Supplement: Attachment — Submitted filename: Reviewer comments- PCT in AECOPD.docx [file pone.0312099.s007.docx]

The Article is a systematic review and meta- analysis of prognostic value of Procalcitonin in acute exacerbation of COPD.

The authors have highlighted the role of PCT at time of admission in predicting the mortality in patients with AECOPD. However, the following suggestions can be incorporated.

1.	In the introduction (Line 51), grammatical errors are there. ‘Negatively impacts on health status’. It should be ‘negative impact on health’.

2.	In the results, 8 clinical trials with 2085 patients are mentioned however, the authors have mentioned that fourteen clinical trials were taken into consideration for meta- analysis in the discussion. Pl clarify.

3.	The discussion is not lucid as the authors have not clearly explained the results. The discussion meanders a bit and should be a bit more elaborate.

4.	The authors have contradicted themselves. In the results, it is mentioned that no significant association between PCT levels and mortality was found. However, in the conclusion the authors have mentioned that PCT at the time of admission positively corelated with mortality. Please explain and clarify.

5.	The Forest plots have not been labelled.

6.	The authors have neither mentioned about the findings of sub- group analysis (as shown in the Forest plot) in the results nor have they discussed them in the discussion section.
